# A retro Baeyer–Villiger reaction: electrochemical reduction of [60]fullerene-fused lactones to [60]fullerene-fused ketones†
†Electronic supplementary information (ESI) available: Detailed experimental procedures and characterization data, the NMR spectra, and CVs of **2a–i** and **3a** (PDF). X-ray crystallographic data for **2f** (CIF). CCDC 1856172. For ESI and crystallographic data in CIF or other electronic format see DOI: 10.1039/c8sc05089a


**DOI:** 10.1039/c8sc05089a

**Published:** 2019-01-16

**Authors:** Chuang Niu, Dian-Bing Zhou, Yong Yang, Zheng-Chun Yin, Guan-Wu Wang

**Affiliations:** a Hefei National Laboratory for Physical Sciences at Microscale , CAS Key Laboratory of Soft Matter Chemistry , iChEM (Collaborative Innovation Center of Chemistry for Energy Materials) , Center for Excellence in Molecular Synthesis of CAS , Department of Chemistry , University of Science and Technology of China , Hefei , Anhui 230026 , P. R. China . Email: gwang@ustc.edu.cn; b State Key Laboratory of Applied Organic Chemistry , Lanzhou University , Lanzhou , Gansu 730000 , P. R. China

## Abstract

A highly efficient electrochemical reduction of [60]fullerene-fused lactones to [60]fullerene-fused ketones, a formal process of retro Baeyer–Villiger reaction, has been achieved for the first time. The electrochemically generated dianionic [60]fullerene-fused lactones can be transformed into [60]fullerene-fused ketones in the presence of acetic acid in 85–91% yields. Control experiments have been performed to elucidate the reaction mechanism. The products have been characterized with spectroscopic data and single-crystal X-ray analysis. Moreover, the electrochemical properties have also been investigated.

## Abstract 2

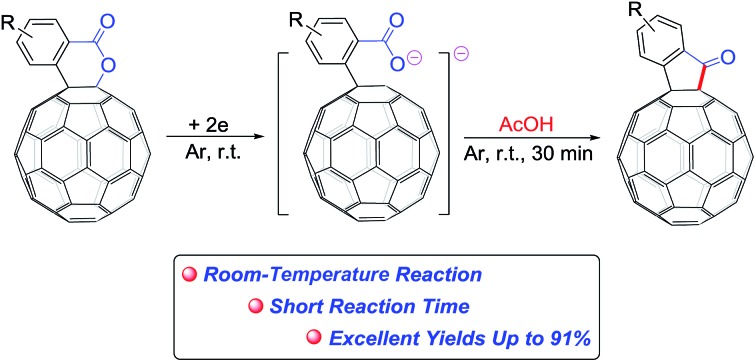
An unprecedented retro Baeyer–Villiger reaction has been achieved by the electrochemical reduction of [60]fullerene-fused lactones in the presence of acetic acid at room temperature, affording [60]fullerene-fused ketones in excellent yields within a short time.

## Introduction

**Scheme 1:**
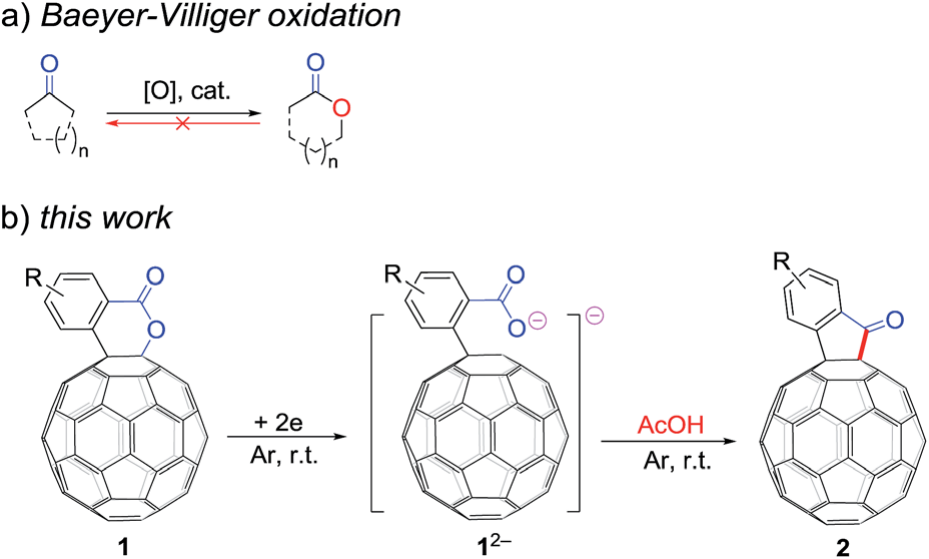
(a) Baeyer–Villiger oxidation. (b) Retro Baeyer–Villiger reaction of C_60_-fused lactones.

The Baeyer–Villiger oxidation is one of the most important transformations in organic synthesis, because valuable esters and lactones can be obtained directly from the corresponding ketones (Scheme 1a).^1^ However, to the best of our knowledge, the retro Baeyer–Villiger reaction, that is, the direct reduction of esters/lactones to ketones accompanied by the elimination of only one oxygen atom *via* either a deoxygenative (–O) or dehydrative (–H_2_O) pathway, has never been reported and remains a challenging task.^2^,^3^

Over the past few decades, fullerene derivatives have attracted much attention due to their potential for application in the fields of biomedical and materials science.^4^ Therefore, a great diversity of synthetic protocols for functionalizing fullerenes have been developed by chemists.^5^,^6^ Among the numerous methods, electrosynthesis has been demonstrated as a novel and efficient strategy due to its mild reaction conditions, good regioselectivity, and relatively high yields.^6^

It has been shown that electrochemically generated fullerene anions, especially singly bonded fullerene dianions, can be readily prepared and used as building blocks in the regioselective synthesis of fullerene derivatives with novel addition patterns.^6^ In an attempt to protonate dianionic [60]fullerene (C_60_)-fused lactones with acetic acid (AcOH), C_60_-fused ketones **2**,^7^ rather than the expected tetrahydrofullerenes,6*i* can be surprisingly obtained in high yields (Scheme 1b). This is the first time the direct reduction of lactones to ketones, which is a formal retro reaction of Baeyer–Villiger oxidation, has been realized. Herein, we report this unprecedented retro Baeyer–Villiger reaction of C_60_-fused lactones by the electrochemical approach.

## Results and discussion

**Fig. 1:**
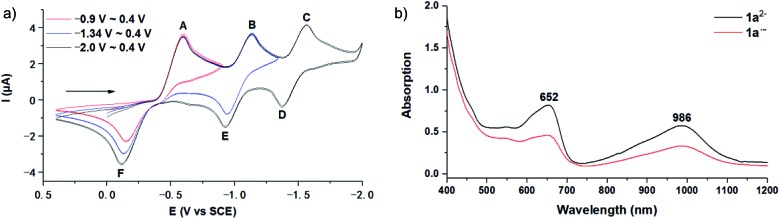
(a) Cyclic voltammograms of compound **1a** (1.0 mM) shown within different potential windows. The CVs recorded in ODCB containing 0.1 M TBAP starting from 0.0 V toward the negative potential with a scan rate of 50 mV s^–1^. The arrows indicate the scan direction for the cyclic voltammetric measurements. (b) Vis/NIR spectra of **1a˙^–^** (red) and **1a^2–^** (black) in ODCB (0.25 mM).

The employed C_60_-fused lactone **1a** was synthesized according to our previous procedure.^8^ Cyclic voltammetry (CV) of **1a** in *o*-dichlorobenzene (ODCB) containing 0.1 M tetra-*n*-butylammonium perchlorate (TBAP) showed that the first redox was an irreversible one-electron transfer process with an *E*_pc_ of –0.60 V (A) *versus* a saturated calomel electrode (SCE), and the second redox was chemically quasi-reversible on the CV timescale with *E*_pc_ at –1.14 V (B) (Fig. 1a), indicating that the compound underwent a chemical reaction process after receiving one electron. The heterolytic cleavage of the C_60_–O bond occurred to provide the ring-opened radical anion **1a˙^–^**, in which the negative charge and unpaired electron were distributed on the fullerene skeleton and/or the carbonyl group, respectively (*vide infra*), once **1a** acquired one electron. Upon acceptance of the second electron, a singly bonded dianionic species **1a^2–^**, in which one negative charge was located at the carboxylate group and another one was distributed on the fullerene cage, was formed.^9^ These ring-opened structures were further confirmed by the visible/near-infrared (Vis/NIR) study of **1a˙^–^** and **1a^2–^**, which were obtained by controlled potential electrolysis (CPE) at –0.90 V and –1.34 V, respectively. The Vis/NIR spectra of **1a˙^–^** and **1a^2–^** (Fig. 1b) showed strong absorption bands at *λ* = 986 and 652 nm, which were in excellent agreement with those of the singly bonded anions of a C_60_-fused oxazoline (*λ* = 963, and 645 nm),9*c* a C_60_-fused sultone (*λ* = 983 and 648 nm),9*d* and a C_60_-fused indoline (*λ* = 966 and 648 nm).9*e*

Controlled potential electrolysis of **1a** (0.015 mmol) in 15.0 mL of anhydrous ODCB solution containing 0.1 M TBAP was carried out at –1.34 V to obtain **1a^2–^** under an argon atmosphere at ambient temperature (∼25 °C). With an aim to protonate **1a^2–^**, AcOH (10 equiv.) was added, and the reaction mixture was stirred at room temperature for 30 min. To our surprise, an intriguing product, C_60_-fused ketone **2a**, was obtained in 91% yield. Importantly, this unexpected dehydrative retro Baeyer–Villiger reaction could be extended to other C_60_-fused lactones, and the results are summarized in Table 1. C_60_-fused lactones with electron-donating groups including the methyl and methoxy groups as well as electron-withdrawing groups such as the chloro and carbonyl groups at different positions of the aromatic ring afforded **2a–g** in excellent yields of 86–91%. Detailed comparisons of these results showed that the electronic properties (entries 1–4 *vs.* entries 5–7) and locations (entry 1 *vs.* entry 2, entry 3 *vs.* entry 4, and entry 5 *vs.* entry 6) of the substituents on the phenyl ring had little effect on the product yields, indicating that the ring-closure of **1a–g^2–^** to afford **2a–g** was a highly efficient process. In addition, when the di-substituted substrate with two methoxy groups was employed, the corresponding product **2h** could also be obtained smoothly in 85% yield. Finally, C_60_-fused lactone **1i** with no substituent on the phenyl ring gave the simplest C_60_-fused ketone **2i** in 90% yield.

**Table 1:** Results for the reaction of dianionic **1a–i^2–^** with AcOH^*a*^

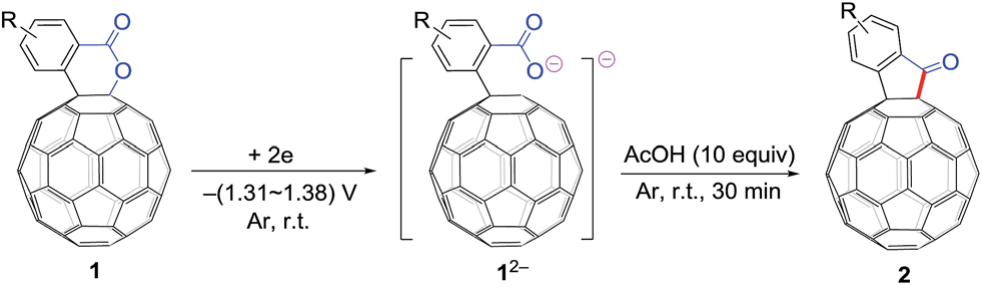									
Entry	C_60_-fused lactone **1**	Potential (V)	Product **2**	Yield^*b*^ (%)	Entry	C_60_-fused lactone **1**	Potential (V)	Product **2**	Yield^*b*^ (%)
1	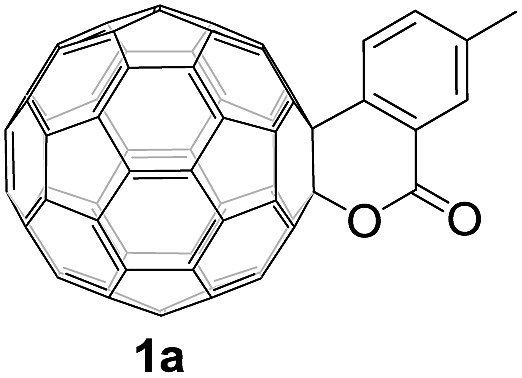	–1.34	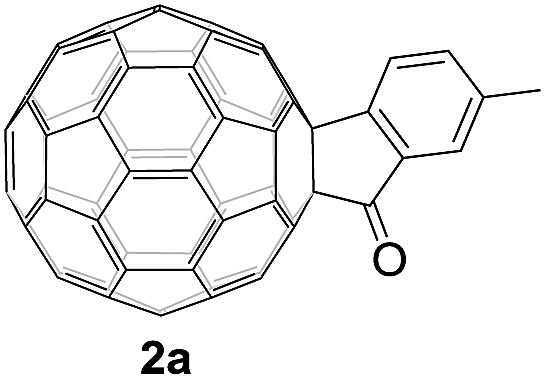	91	6	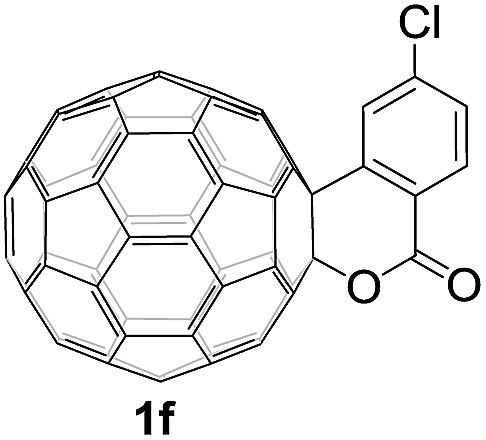	–1.31	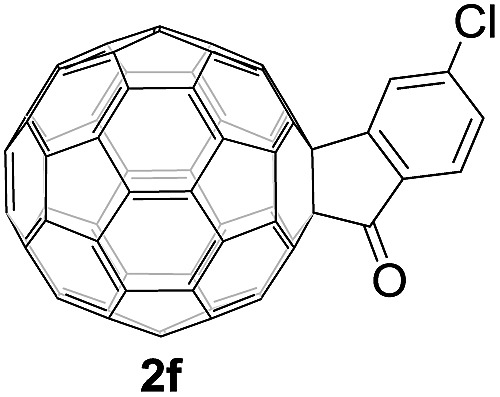	90
2	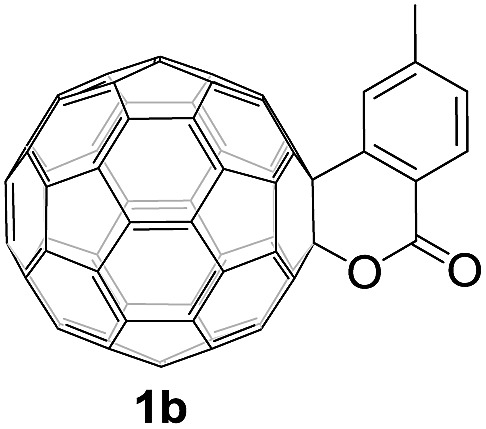	–1.38	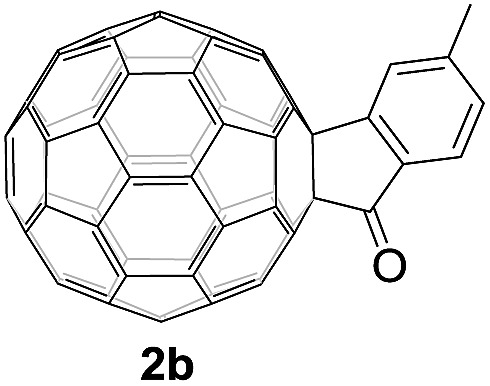	90	7	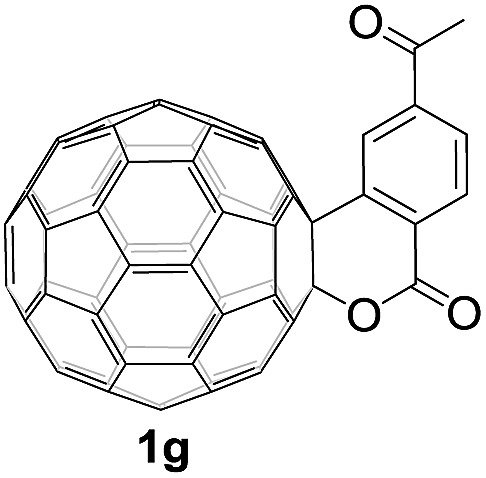	–1.38	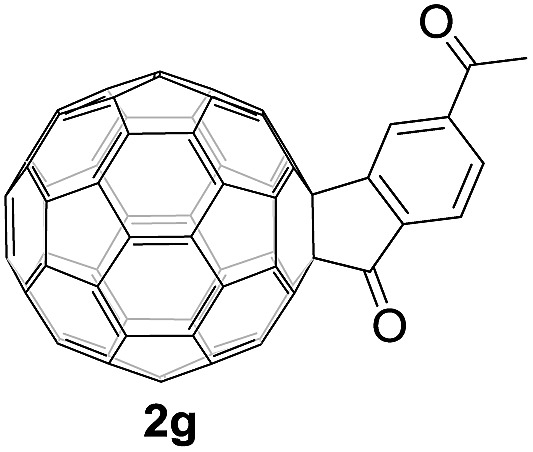	88
3	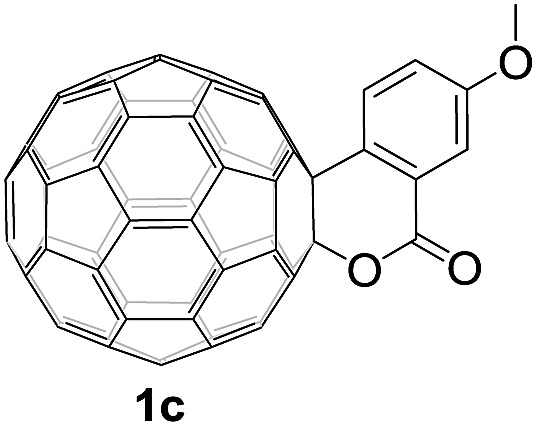	–1.38	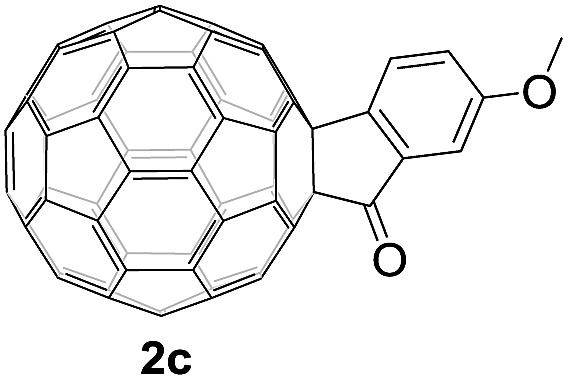	89	8	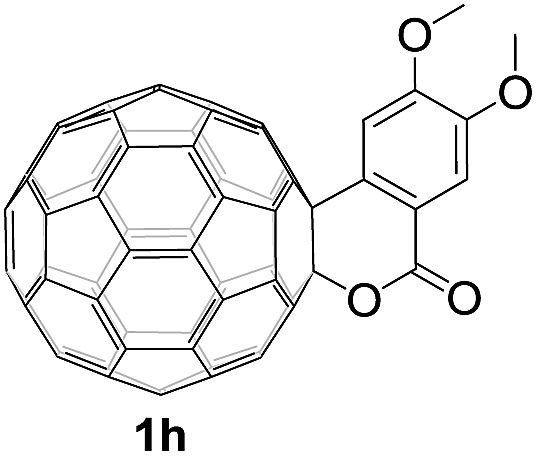	–1.36	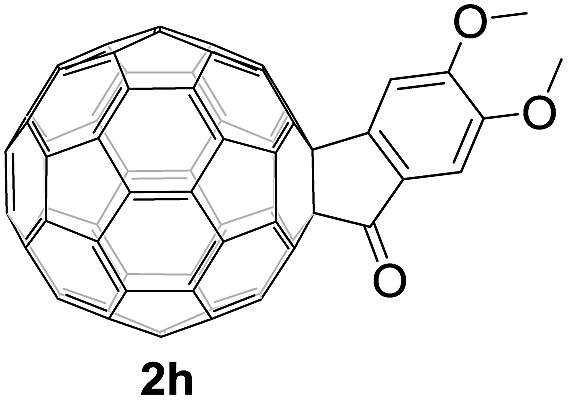	85
4	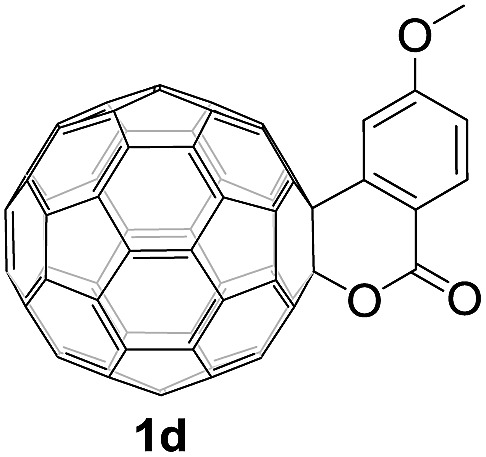	–1.38	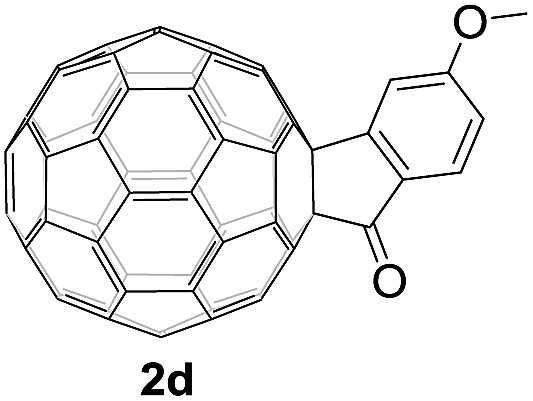	91	9	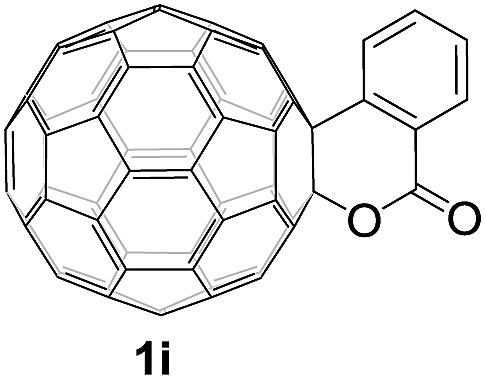	–1.36	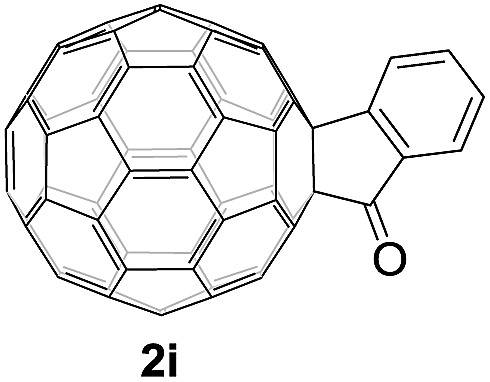	90
5	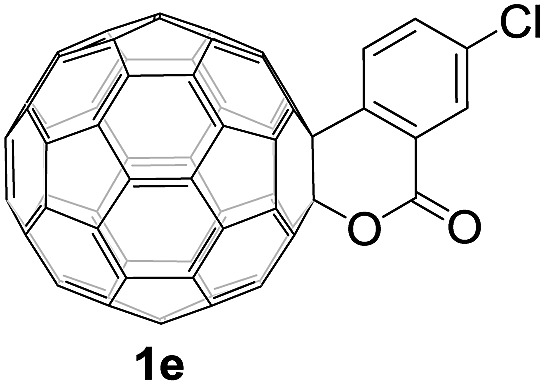	–1.38	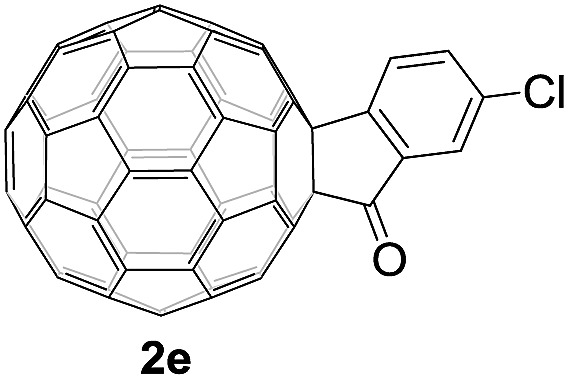	86					

^*a*^All the reactions were performed with 0.015 mmol of **1a–i^2–^** and 0.150 mmol of acetic acid at room temperature (∼25 °C) for 30 min under an argon atmosphere.

^*b*^Isolated yield.

**Fig. 2:**
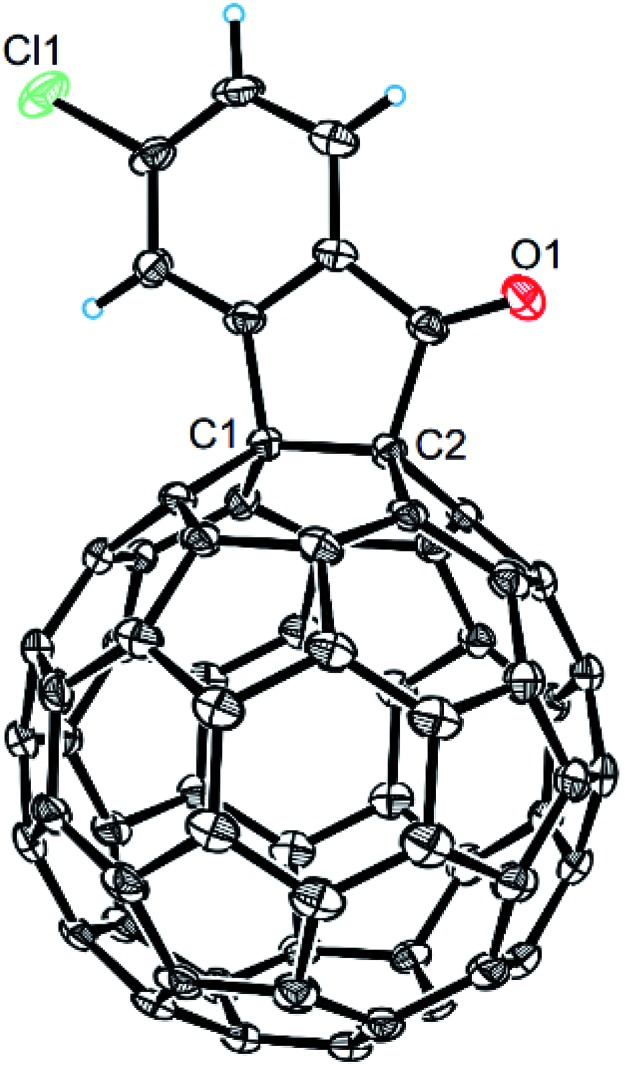
ORTEP diagram for one enantiomer of **2f** with thermal ellipsoids shown at 50% probability. The toluene molecule is omitted for clarity.

The structures of products **2a–i** were unambiguously characterized by MALDI-TOF MS, ^1^H NMR, ^13^C NMR, FT-IR, and UV-vis spectrometry. All mass spectra of these products exhibited the correct [M]^+^ peaks. Their ^1^H NMR spectra displayed the expected chemical shifts as well as the splitting patterns for all protons. The ^13^C NMR spectra of **2a–i** exhibited no more than 30 peaks in the range of 135–159 ppm for the 58 sp^2^-carbons of the fullerene cage and two peaks at 70–80 ppm for the two sp^3^-carbons of the fullerene skeleton, consistent with the *C*_s_ symmetry of their molecular structures. Their UV-vis spectra exhibited a peak at 430–432 nm, which corresponds to the diagnostic absorption of 1,2-adducts of C_60_ at the [6,6]-junction. The structures of products were unambiguously confirmed by the single-crystal X-ray diffraction analysis of **2f** as an example (Fig. 2).^10^

**Scheme 2:**
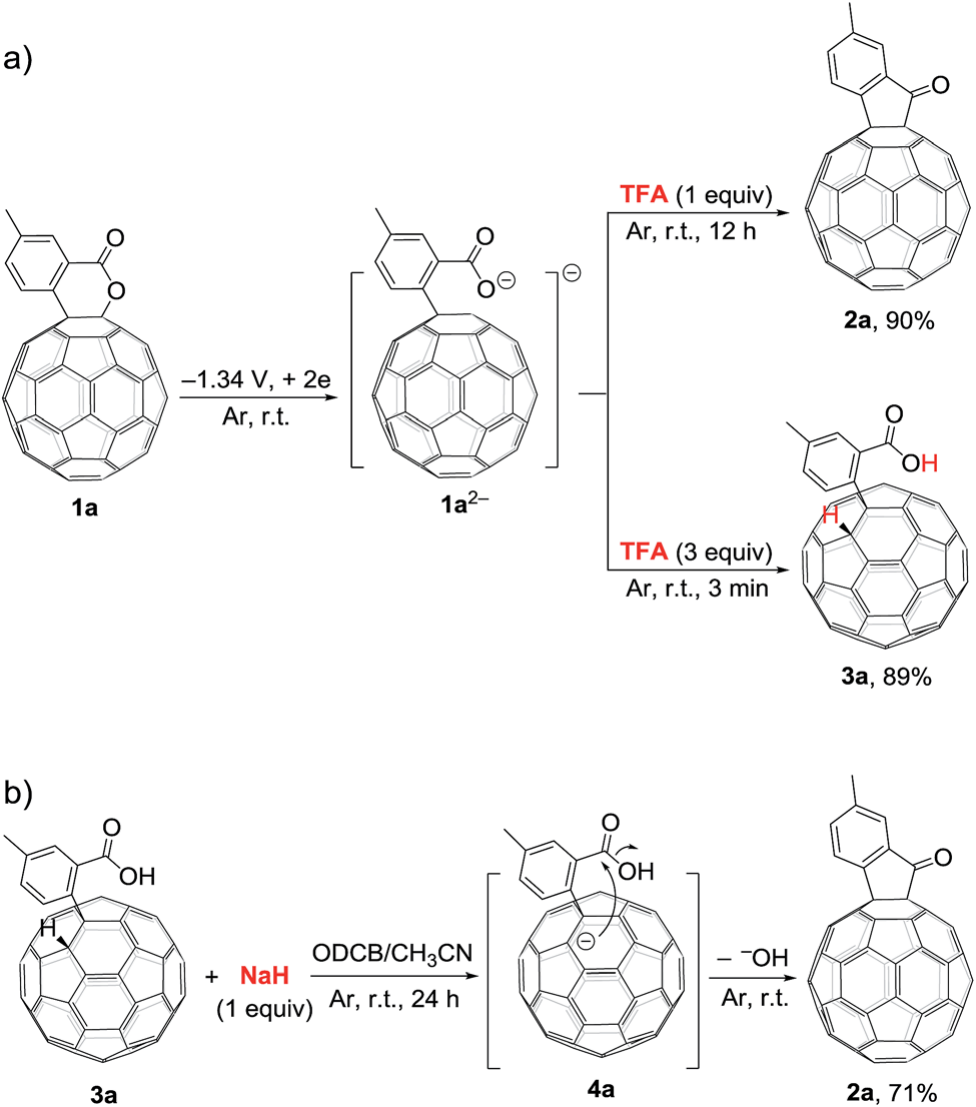
Control experiments.

During the screening of the added acids, it was intriguingly found that different amounts of trifluoroacetic acid (TFA) afforded different products. When **1a^2–^** was treated with 1 equiv. of TFA, **2a** could also be obtained in 90% yield, but required a long reaction time of 12 h. However, when **1a^2–^** was reacted with 3 equiv. of TFA for only 3 min, hydrofullerene **3a** was obtained in 89% yield (Scheme 2a). The structure of **3a** was established by its spectral data, particularly the singlet at *δ*_H_ = 6.89 ppm for the diagnostic fullerenyl proton in its ^1^H NMR spectrum.6f,i,j,9a,e,^11^ Additional control experiments showed that treatment of **3a** with 1 equiv. of sodium hydride (NaH) in a mixture of ODCB and CH_3_CN (4 : 1) at room temperature under an argon atmosphere provided **2a** in 71% yield (Scheme 2b). The reported p*K*_a_ values of TFA, *t*-BuC_60_H, PhCO_2_H, and AcOH in DMSO were 3.45, 5.7, 11.1, and 12.3, respectively.^12^ Although their corresponding p*K*_a_ values in ODCB or a mixture of ODCB and CH_3_CN are unavailable, it is reasonable to assume that the relative p*K*_a_ values of the same order are retained in these solvent systems. Therefore, it is expected that TFA would first protonate the carboxylate anion and then the fullerenyl anion. When only 1 equiv. of TFA was added, the carboxylate anion of **1a^2–^** would be preferably protonated, and subsequent intramolecular cyclization by the attack of the fullerenyl anion to the formed carboxyl group afforded C_60_-fused ketone **2a**. In comparison, when excess amounts (3 equiv.) of TFA were added, both the carboxylate anion and the fullerenyl anion were protonated to give hydrofullerene **3a** as the most stable 1,2-adduct. On the other hand, 1 equiv. of NaH would selectively deprotonate the more acidic fullerenyl proton rather than the carboxyl group of **3a**, followed by a cyclization process to provide **2a**.

**Scheme 3:**
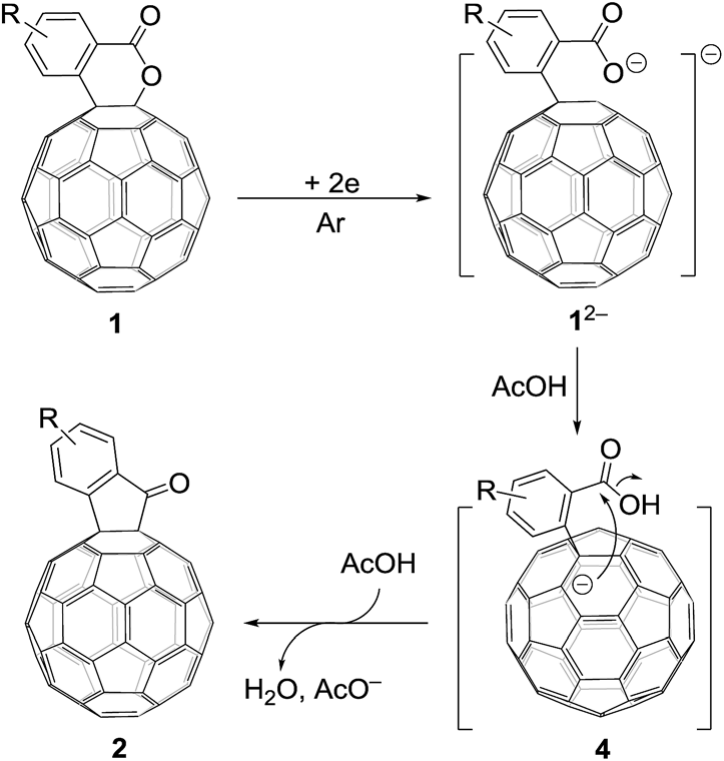
Proposed reaction mechanism for the formation of C_60_-fused ketones from C_60_-fused lactones.

Based on the above results and previous literature,^9^ a plausible reaction mechanism for the formation of **2** is depicted in Scheme 3. Firstly, C_60_-fused lactone **1** is electrochemically reduced with a cleavage of the C–O bond to generate ring-opened dianionic **1^2–^**. Since AcOH is the weakest acid in the order of the above-mentioned acids (TFA, *t*-BuC_60_H, PhCO_2_H and AcOH), only the carboxylate anion of dianion **1^2–^** seems to be protonated even in the presence of excess AcOH to give monoanion **4**. Finally, intermediate **4** undergoes intramolecular cyclization accompanied by the removal of the hydroxide ion, which is assisted by the neutralization with another molecule of AcOH, to provide product **2**.

**Scheme 4:**
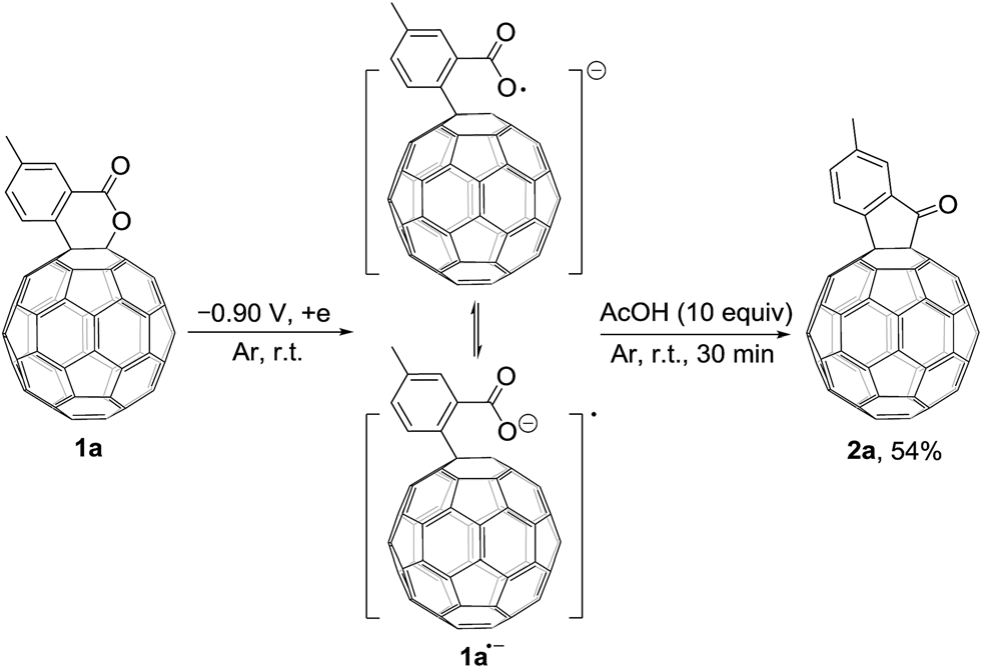
Synthesis of **2a** by the reaction of **1a˙^–^** with AcOH.

We also explored the possibility for the retro Baeyer–Villiger reaction of C_60_-fused lactones by utilization of their radical monoanions with **1a** as an example. The irreversible first redox process in the CV of **1a** (Fig. 1a) hinted that its lactone moiety would rupture to provide the ring-opened **1a˙^–^** after receiving one electron. The Vis/NIR spectrum of **1a˙^–^** showed significantly lower intensities at 986 and 652 nm than that of **1^2–^** at the same concentration (Fig. 1b), suggesting that only some of **1a˙^–^** had a ring-opened structure with the negative charge distributed on the fullerene skeleton. The synthesis of **2a** by the reaction of **1a˙^–^** with 10 equiv. of AcOH was attempted, yet **2a** could be obtained in only 54% yield (Scheme 4), much lower than that (91%) from the reaction of **1^2–^**. The exact reaction pathway leading to **2a** is not clear and currently under investigation. Therefore, the retro Baeyer–Villiger reaction of C_60_-fused lactones was much more efficiently achieved through their dianionic intermediates rather than with their radical monoanionic species.

The half-wave reduction potentials of C_60_-fused ketones **2a–i** and hydrofullerene **3a** along with those of C_60_ were investigated by CV and are summarized in Table 2. All of their electrochemical properties were quite similar and showed two reversible redox processes. As shown in Table 2, the first reduction potentials of products **2a–i** and **3a** were more negative than that of C_60_, indicating that they possess higher LUMO energy levels than C_60_ and may have potential for application in organic photovoltaic devices as acceptors.^13^

**Table 2:** Half-wave reduction potentials (V) of C_60_ and compounds **2a–f** and **3a**^*a*^

Compd	*E*_1_	*E*_2_
C_60_	–1.076	–1.460
**2a**	–1.121	–1.503
**2b**	–1.127	–1.522
**2c**	–1.125	–1.520
**2d**	–1.128	–1.522
**2e**	–1.106	–1.495
**2f**	–1.104	–1.500
**2g**	–1.103	–1.490
**2h**	–1.117	–1.499
**2i**	–1.111	–1.483
**3a**	–1.130	–1.518

^*a*^ *Versus* ferrocene/ferrocenium. Experimental conditions: 1.0 mM compound and 0.1 M TBAP in anhydrous ODCB; reference electrode: SCE; working electrode: Pt disc; auxiliary electrode: Pt wire; scan rate: 50 mV s^–1^.

## Conclusions

In summary, we have achieved a highly efficient synthesis of various C_60_-fused ketones from C_60_-fused lactones for the first time *via* electrochemical reduction, an unprecedented dehydrative retro Baeyer–Villiger reaction. The present protocol shows advantages of mild reaction conditions, a short reaction time, excellent product yields, and remarkable functional group tolerance. Moreover, control experiments have been performed to elucidate the plausible reaction mechanism for the formation of C_60_-fused ketones. The electrochemical properties of the synthesized C_60_-fused ketones have been characterized and may be utilized in solar cell devices.

## Conflicts of interest

The authors declare no conflict of interest.

## Supplementary Material

Supplementary information

Crystal structure data
