# Supplementary material for: A retro Baeyer–Villiger reaction: electrochemical reduction of [60]fullerene-fused lactones to [60]fullerene-fused ketones
Source: Chem Sci. 2019 Jan 16;10(10):3012–7. doi: 10.1039/c8sc05089a (PMC6427942; doi:10.1039/c8sc05089a)
Supplement: Supplementary file 1 [file SC-010-C8SC05089A-s001.pdf]

Electronic Supplementary Material (ESI) for Chemical Science.
This journal is © The Royal Society of Chemistry 2019

Electronic Supplementary Information

Retro Baeyer–Villiger Reaction: Electrochemical Reduction of [60]Fullerene-Fused Lactones to [60]Fullerene-Fused Ketones

Chuang Niu,[a] Dian-Bing Zhou,[a] Yong Yang,[a] Zheng-Chun Yin,[a] and Guan-Wu Wang*,[a,b]

[a] *Hefei National Laboratory for Physical Sciences at Microscale, CAS Key Laboratory of Soft Matter Chemistry, iChEM (Collaborative Innovation Center of Chemistry for Energy Materials), Center for Excellence in Molecular Synthesis of CAS, and Department of Chemistry, University of Science and Technology of China, Hefei, Anhui 230026, P. R. China E-mail: gwang@ustc.edu.cn; Fax: +86 551 3607864; Tel: +86 551 3607864*

[b] *State Key Laboratory of Applied Organic Chemistry, Lanzhou University, Lanzhou, Gansu 730000, P. R. China*

Table of Contents

1. Experimental Procedures and Spectral Data of **2a**–**2i** S2
2. X-Ray Data of Compound **2f** S9
3. Mechanism Studies S10
4. NMR Spectra of Compounds **2a**–**i** and **3a** S13
5. CVs of Compounds **1a**–**i**, **2a**-**i**, **3a** along with C_{60} S31
6. Reference S41

1. Experimental Procedures and Spectral Data of 2a–2i

1.1. General Methods

All electrochemical reactions were performed under argon atmosphere at room temperature (~25 °C) using a SHANGHAI CHENHUA CHI630D workstation. Tetra-*n*-butylammonium perchlorate (TBAP) was recrystallized from absolute ethanol and dried in a vacuum at 313 K prior to use. A conventional three-electrode cell was used for CV measurements and consisted of a 2 mm diameter platinum disc working electrode, a platinum wire auxiliary electrode, and a saturated calomel reference electrode (SCE). Controlled potential electrolysis (CPE) was carried out on a potentiostat/galvanostat using an "H" type cell consisting of two platinum gauze electrodes (serving as working and counter electrodes, respectively) and a SCE. The SCE was separated from the bulk of the solution by a fritted-glass bridge of low porosity, which contained the solvent/supporting electrolyte mixture. Compounds **1a–i** were synthesized according to the procedure developed by our group.[1] Other chemicals were obtained commercially and used without further purification.

1.2. Cyclic Voltammetry of 1a

Compound **1a** (1.0 mM) dissolved in anhydrous *o*-dichlorobenzene (ODCB) containing 0.1 M TBAP was added into an electrochemical cell under an argon atmosphere at room temperature. CV measurement was then undertaken at a scan rate of 50 mV s^{-1}.

1.3. Vis/NIR Spectral Measurement of the Dianion of 1a

3.2 mg (37.5 μmol) of **1a** was electroreduced by CPE at –1.34 V vs SCE in 15.0 mL of anhydrous ODCB solution containing 0.1 M TBAP under an argon atmosphere. The electrolysis was terminated when the theoretical number of coulombs required for a full conversion of **1a** to **1a**$^{2-}$ was reached. A green-colored solution was obtained after the electrolysis, which is the typical color for C_{60} anionic species bonded with only one addend. The resulted solution was transferred to a 10-mm quartz cuvette under argon and sealed with a rubber septum and Parafilm for the Vis/NIR measurement.

1.4. Vis/NIR Spectral Measurement of the Radical Anion of 1a

3.2 mg (37.5 μmol) of **1a** was electroreduced by CPE at –0.90 V vs SCE in 15.0 mL of anhydrous ODCB solution containing 0.1 M TBAP under an argon atmosphere. The electrolysis was terminated when the theoretical number of coulombs required for a full conversion of **1a** to **1a**$^{\cdot-}$ was reached. A light green-colored solution was obtained after the electrolysis, which is the typical color for C_{60} anionic species bonded with only one addend. The resulted solution was transferred to a 10-mm quartz cuvette under argon and sealed with a rubber septum and Parafilm for the Vis/NIR measurement.

1.5. Preparation of the Dianions 1a–i^{2-}

0.015 mmol of **1a–i** was dissolved in 15.0 mL of anhydrous ODCB solution containing 0.1 M TBAP. Then the solution was electroreduced by CPE at –(1.31~1.38)

V vs SCE under an argon atmosphere at room temperature. When the theoretical number of coulombs required for a full conversion of **1a–i** to **1a–i** $^{2-}$ was reached, the electrolysis was terminated after about 1 h, and a dark-green solution of **1a–i** $^{2-}$ was obtained.

1.6. Preparation of the Radical Anion 1a$^{\cdot-}$

0.015 mmol of **1a** was dissolved in 15.0 mL of anhydrous ODCB solution containing 0.1 M TBAP. Then the solution was electroreduced by CPE at –0.90 V vs SCE under an argon atmosphere at room temperature. When the theoretical number of coulombs required for a full conversion of **1a** to **1a**$^{\cdot-}$ was reached, the electrolysis was terminated after about 40 min, and a green solution of **1a**$^{\cdot-}$ was obtained.

1.7. Synthesis of Compound 2a

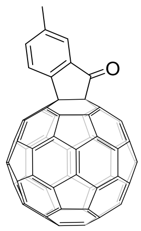

2a

Compound **1a** (12.8 mg, 0.015 mmol) was electroreduced by CPE at –1.34 V vs SCE, then the dianion **1a**$^{2-}$ reacted with acetic acid (AcOH) (8.6 μL, 0.15 mmol). After being stirred at room temperature for 0.5 h, the resulting mixture was directly filtered through a silica gel (200–300 mesh) plug with CS_2/CH_2Cl_2 (1:1 v/v) to remove the supporting electrolyte and insoluble materials, and then evaporated in vacuo to remove the solvent. Next, the residue was further separated on a silica gel column (300–400 mesh) with CS_2 as the eluent to afford product **2a** (11.5 mg, 91%) as an amorphous brown solid along. ^{1}H NMR (400 MHz, 1:1 $CS_2/CDCl_3$) δ 8.36 (d, J = 7.9 Hz, 1H), 8.20 (brs, 1H), 7.86 (dd, J = 7.9, 1.4 Hz, 1H), 2.72 (s, 3H); ^{13}C NMR (100 MHz, 1:1 $CS_2/CDCl_3$ with $Cr(acac)_3$ as relaxation reagent) (all 2C unless indicated) δ 197.88 (1C, *C*=*O*), 153.85, 153.73, 153.48, 147.56, 147.26 (1C), 146.99 (1C), 146.21 (4C), 145.98, 145.92, 145.71, 145.49, 145.39, 145.34, 145.22 (4C), 144.50, 144.14, 142.99, 142.61, 142.55, 142.27, 142.05, 141.94 (4C), 141.74, 141.56, 140.47, 140.44 (1C, aryl *C*), 140.40, 138.21 (1C, aryl *C*), 135.32, 135.32 (1C, aryl *C*), 134.35 (1C, aryl *C*), 127.06 (1C, aryl *C*), 126.33 (1C, aryl *C*), 78.95 (1C, sp^{3}-*C* of C_{60}), 70.49 (1C, sp^{3}-*C* of C_{60}), 21.52 (1C); FT-IR ν/cm^{-1} (KBr) 2919, 2851, 1717, 1580, 1502, 1424, 1274, 1235, 1150, 1092, 1019, 958, 814, 774, 693, 583, 559, 526; UV-vis ($CHCl_3$) λ_{max}/nm (log ε) 268 (4.95), 302 (4.70), 431 (3.60), 698 (2.61); MALDI-TOF MS *m/z* calcd for $C_{68}H_6O$ $[M]^+$ 838.0413, found 838.0401.

1.8. Synthesis of Compound 2b

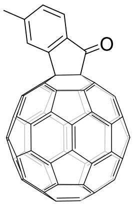

2b

Compound **1b** (12.8 mg, 0.015 mmol) was electroreduced by CPE at –1.38 V vs SCE, then the dianion $\mathbf{1b}^{2-}$ reacted with AcOH (8.6 μL, 0.15 mmol). After being stirred at room temperature for 0.5 h, the resulting mixture was directly filtered through a silica gel (200–300 mesh) plug with CS_2/CH_2Cl_2 (1:1 v/v) to remove the supporting electrolyte and insoluble materials, and then evaporated in vacuo to remove the solvent. Next, the residue was further separated on a silica gel column (300–400 mesh) with CS_2 as the eluent to afford product **2b** (11.3 mg, 90%) as an amorphous brown solid along. ^{1}H NMR (400 MHz, 1:1 $CS_2/CDCl_3$) δ 8.31 (d, J = 8.0 Hz, 1H), 8.30 (s, 1H), 7.65 (dd, J = 8.0, 0.7 Hz, 1H), 2.72 (s, 3H); ^{13}C NMR (100 MHz, 1:1 $CS_2/CDCl_3$ with $Cr(acac)_3$ as relaxation reagent) (all 2C unless indicated) δ 197.16 (1C, *C=O*), 156.47, 153.66, 153.32, 148.52 (1C, aryl *C*), 147.49, 147.16 (1C), 146.87 (1C), 146.10, 146.08, 145.87, 145.80, 145.64, 145.39, 145.27, 145.21, 145.12, 145.10, 144.39, 144.01, 142.87, 142.49, 142.44, 142.15, 141.91, 141.84, 141.82, 141.61, 141.47, 140.38, 140.26, 135.25, 135.13 (1C, aryl *C*), 131.57 (1C, aryl *C*), 131.34 (1C, aryl *C*), 126.99 (1C, aryl *C*), 126.86 (1C, aryl *C*), 78.83 (1C, sp^3-*C* of C_{60}), 70.52 (1C, sp^3-*C* of C_{60}), 22.38 (1C); FT-IR ν/cm^{-1} (KBr) 2920, 1714, 1594, 1503, 1424, 1263, 1220, 1182, 1134, 1082, 1005, 948, 835, 815, 731, 693, 580, 560, 524; UV-vis ($CHCl_3$) λ_{max}/nm (log ε) 267 (4.98), 304 (4.76), 431 (3.65), 698 (2.65); MALDI-TOF MS *m/z* calcd for $C_{68}H_6O$ [M^+] 838.0413, found 838.0425.

1.9. Synthesis of Compound 2c

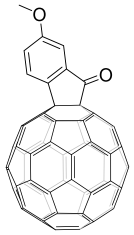

2c

Compound **1c** (13.1 mg, 0.015 mmol) was electroreduced by CPE at –1.38 V vs SCE, then the dianion $\mathbf{1c}^{2-}$ reacted with AcOH (8.6 μL, 0.15 mmol). After being stirred at room temperature for 0.5 h, the resulting mixture was directly filtered through a silica gel (200–300 mesh) plug with CS_2/CH_2Cl_2 (1:1 v/v) to remove the supporting electrolyte and insoluble materials, and then evaporated in vacuo to remove the solvent. Next, the residue was further separated on a silica gel column (300–400 mesh) with CS_2/CH_2Cl_2 (4:1 v/v) as the eluent to afford product **2c** (11.5 mg, 89%) as an amorphous brown solid along. ^{1}H NMR (400 MHz, 1:1 $CS_2/CDCl_3$) δ 8.34 (d, J = 8.5 Hz, 1H), 7.78 (d, J = 2.6 Hz, 1H), 7.61 (dd, J = 8.5, 2.6 Hz, 1H), 4.09 (s, 3H); ^{13}C NMR (100 MHz,

1:1 CS_2/$CDCl_3$ with Cr(acac)$_3$ as relaxation reagent) (all 2C unless indicated) δ 198.24 (1C, *C*=*O*), 161.25 (1C, aryl *C*), 154.04, 153.51, 149.18, 147.50, 147.30 (1C), 147.04 (1C), 146.25, 146.22, 146.01, 145.95, 145.63, 145.50, 145.41, 145.36, 145.25 (4C), 144.52, 144.19, 143.00, 142.64, 142.57, 142.29, 142.11, 141.96 (4C), 141.77, 141.55, 140.45, 140.42, 135.62 (1C, aryl *C*), 135.48 (1C, aryl *C*), 135.34, 127.26 (1C, aryl *C*), 126.62 (1C, aryl *C*), 107.55 (1C, aryl *C*), 79.27 (1C, sp^3-*C* of C_{60}), 70.24 (1C, sp^3-*C* of C_{60}), 55.85 (1C); FT-IR ν/cm^{-1} (KBr) 2935, 2836, 1713, 1597, 1497, 1426, 1334, 1274, 1239, 1176, 1096, 1015, 959, 874, 830, 772, 693, 586, 558, 519; UV-vis ($CHCl_3$) λ_{max}/nm (log ε) 261 (4.94), 304 (4.66), 431 (3.54), 699 (2.52); MALDI-TOF MS *m/z* calcd for $C_{68}H_6O_2$ $[M]^+$ 854.0362, found 854.0379.

1.10. Synthesis of Compound 2d

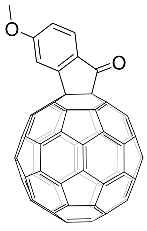

2d

Compound **1d** (13.1 mg, 0.015 mmol) was electroreduced by CPE at –1.38 V vs SCE, then the dianion **1d**$^{2-}$ reacted with AcOH (8.6 μL, 0.15 mmol). After being stirred at room temperature for 0.5 h, the resulting mixture was directly filtered through a silica gel (200–300 mesh) plug with CS_2/CH_2Cl_2 (1:1 v/v) to remove the supporting electrolyte and insoluble materials, and then evaporated in vacuo to remove the solvent. Next, the residue was further separated on a silica gel column (300–400 mesh) with CS_2/CH_2Cl_2 (4:1 v/v) as the eluent to afford product **2d** (11.7 mg, 91%) as an amorphous brown solid along. ^{1}H NMR (400 MHz, 1:1 CS_2/$CDCl_3$) δ 8.32 (d, *J* = 8.6 Hz, 1H), 7.86 (d, *J* = 2.2 Hz, 1H), 7.35 (dd, *J* = 8.6, 2.2 Hz, 1H), 4.08 (s, 3H); ^{13}C NMR (100 MHz, 1:1 CS_2/$CDCl_3$ with Cr(acac)$_3$ as relaxation reagent) (all 2C unless indicated) δ 195.56 (1C, *C*=*O*), 166.86 (1C, aryl *C*), 158.83, 153.39, 153.05, 147.45, 147.07 (1C), 146.75 (1C), 146.01, 145.96, 145.76, 145.68, 145.51, 145.30, 145.18, 145.09, 145.02, 144.99, 144.31, 143.87, 142.77, 142.40, 142.34, 142.07, 141.79, 141.72, 141.70, 141.49, 141.40, 140.33, 140.16, 135.29, 134.93 (1C, aryl *C*), 128.62 (1C, aryl *C*), 126.25 (1C, aryl *C*), 118.49 (1C, aryl *C*), 109.30 (1C, aryl *C*), 78.90 (1C, sp^3-*C* of C_{60}), 70.32 (1C, sp^3-*C* of C_{60}), 55.82 (1C); FT-IR ν/cm^{-1} (KBr) 2931, 2837, 1709, 1589, 1493, 1428, 1336, 1266, 1225, 1182, 1139, 1081, 1008, 944, 857, 807, 717, 691, 579, 559, 517; UV-vis ($CHCl_3$) λ_{max}/nm (log ε) 268 (4.96), 301 (4.60), 431 (3.63), 700 (2.61); MALDI-TOF MS *m/z* calcd for $C_{68}H_6O_2$ $[M^+]$ 854.0362, found 854.0389.

1.11. Synthesis of Compound 2e

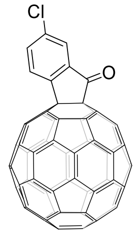

2e

Compound **1e** (13.1 mg, 0.015 mmol) was electroreduced by CPE at –1.38 V vs SCE, then the dianion $\mathbf{1e}^{2-}$ reacted with AcOH (8.6 μL, 0.15 mmol). After being stirred at room temperature for 0.5 h, the resulting mixture was directly filtered through a silica gel (200–300 mesh) plug with CS_2/CH_2Cl_2 (1:1 v/v) to remove the supporting electrolyte and insoluble materials, and then evaporated in vacuo to remove the solvent. Next, the residue was further separated on a silica gel column (300–400 mesh) with CS_2 as the eluent to afford product **2e** (11.1 mg, 86%) as an amorphous brown solid along. ^{1}H NMR (400 MHz, 1:1 $CS_2/CDCl_3$) δ 8.43 (d, J = 8.2 Hz, 1H), 8.36 (d, J = 2.0 Hz, 1H), 8.00 (dd, J = 8.2, 2.0 Hz, 1H); ^{13}C NMR (100 MHz, 1:1 $CS_2/CDCl_3$ with $Cr(acac)_3$ as relaxation reagent) (all 2C unless indicated) δ 197.18 (1C, *C*=*O*), 154.35, 153.22, 152.64, 147.26 (1C), 147.22, 146.99 (1C), 146.20 (4C), 145.97, 145.91, 145.42, 145.38, 145.36, 145.34, 145.23, 145.20, 144.43, 144.06, 142.95, 142.60, 142.53, 142.23, 141.89 (6C), 141.70, 141.43, 140.46, 140.40, 136.99 (1C, aryl *C*), 136.66 (1C, aryl *C*), 135.77 (1C, aryl *C*), 135.40 (1C, aryl *C*), 135.26, 127.78 (1C, aryl *C*), 126.59 (1C, aryl *C*), 78.64 (1C, sp^3-*C* of C_{60}), 70.15 (1C, sp^3-*C* of C_{60}); FT-IR ν/cm^{-1} (KBr) 2921, 2854, 1721, 1583, 1460, 1420, 1306, 1217, 1181, 1082, 1013, 895, 832, 739, 693, 630, 585, 558, 529; UV-vis ($CHCl_3$) λ_{max}/nm (log ε) 257 (4.96), 307 (4.63), 430 (3.51), 694 (2.51); MALDI-TOF MS *m/z* calcd for $C_{67}H_3{}^{35}ClO$ $[M]^+$ 857.9867, found 857.9833.

1.12. Synthesis of Compound 2f

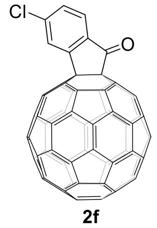

2f

Compound **1f** (13.1 mg, 0.015 mmol) was electroreduced by CPE at –1.31 V vs SCE, then the dianion $\mathbf{1f}^{2-}$ reacted with AcOH (8.6 μL, 0.15 mmol). After being stirred at room temperature for 0.5 h, the resulting mixture was directly filtered through a silica gel (200–300 mesh) plug with CS_2/CH_2Cl_2 (1:1 v/v) to remove the supporting electrolyte and insoluble materials, and then evaporated in vacuo to remove the solvent. Next, the residue was further separated on a silica gel column (300–400 mesh) with CS_2 as the eluent to afford product **2f** (11.6 mg, 90%) as an amorphous brown solid along. ^{1}H NMR (400 MHz, 1:1 $CS_2/CDCl_3$) δ 8.48 (d, J = 1.7 Hz, 1H), 8.36 (d, J = 8.2 Hz, 1H), 7.82 (dd, J = 8.2, 1.7 Hz, 1H); ^{13}C NMR (100 MHz, 1:1 $CS_2/CDCl_3$ with $Cr(acac)_3$ as relaxation reagent) (all 2C unless indicated) δ 197.02 (1C, *C*=*O*), 157.72,

153.22, 152.57, 147.41, 147.39 (1C), 147.13 (1C), 146.36, 146.33, 146.10, 146.05, 145.61, 145.52 (4C), 145.46, 145.38, 145.33, 144.56, 144.20, 144.15 (1C, aryl *C*), 143.08, 142.73, 142.67, 142.36, 142.06, 142.03, 141.98, 141.87, 141.60, 140.63, 140.59, 135.61 (1C, aryl *C*), 135.34, 132.53 (1C, aryl *C*), 131.04 (1C, aryl *C*), 128.22 (1C, aryl *C*), 127.12 (1C, aryl *C*), 78.65 (1C, sp^3-*C* of C_{60}), 70.29 (1C, sp^3-*C* of C_{60}); FT-IR ν/cm^{-1} (KBr) 2921, 2849, 1720, 1582, 1506, 1415, 1312, 1260, 1220, 1185, 1133, 1065, 1007, 955, 836, 814, 767, 676, 559, 519; UV-vis ($CHCl_3$) λ_{max}/nm (log ε) 266 (4.96), 307 (4.71), 430 (3.58), 698 (2.54); MALDI-TOF MS *m/z* calcd for $C_{67}H_3O^{35}Cl$ [M^+] 857.9867, found 857.9876.

1.13. Synthesis of Compound 2g

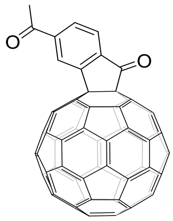

2g

Compound **1g** (13.2 mg, 0.015 mmol) was electroreduced by CPE at –1.38 V vs SCE, then the dianion $\mathbf{1g}^{2-}$ reacted with AcOH (8.6 μL, 0.15 mmol). After being stirred at room temperature for 0.5 h, the resulting mixture was directly filtered through a silica gel (200–300 mesh) plug with CS_2/ethyl acetate (EA) (5:1 v/v) to remove the supporting electrolyte and insoluble materials, and then evaporated in vacuo to remove the solvent. Next, the residue was further separated on a silica gel column (300–400 mesh) with CS_2/CH_2Cl_2 (2:1 v/v) as the eluent to afford product **2g** (11.4 mg, 88%) as an amorphous brown solid along. ^{1}H NMR (400 MHz, 1:1 $CS_2/CDCl_3$) δ 9.02 (brs, 1H), 8.55 (d, J = 8.0 Hz, 1H), 8.40 (dd, J = 8.0, 1.3 Hz, 1H), 2.84 (s, 3H); ^{13}C NMR (100 MHz, 1:1 $CS_2/CDCl_3$ with $Cr(acac)_3$ as relaxation reagent) (all 2C unless indicated) δ 198.13 (1C, *C=O*), 195.81 (1C, *C=O*), 156.68, 153.38, 152.78, 147.41 (1C), 147.36, 147.17 (1C), 146.38 (4C), 146.12, 146.09, 145.65 (4C), 145.55, 145.50, 145.40, 145.37, 144.57, 144.25, 143.87 (1C, aryl *C*), 143.11, 142.76, 142.71, 142.38, 142.09, 142.05, 142.01, 141.92, 141.59, 140.63, 140.62, 137.39 (1C, aryl *C*), 135.53 (1C, aryl *C*), 135.41, 129.81 (1C, aryl *C*), 127.54 (1C, aryl *C*), 126.37 (1C, aryl *C*), 78.81 (1C, sp^3-*C* of C_{60}), 70.70 (1C, sp^3-*C* of C_{60}), 27.05 (1C); FT-IR ν/cm^{-1} (KBr) 2922, 2856, 1720, 1689, 1592, 1503, 1413, 1353, 1264, 1209, 1134, 1092, 1010, 950, 906, 837, 818, 687, 635, 557, 518; UV-vis ($CHCl_3$) λ_{max}/nm (log ε) 259 (4.93), 309 (4.53), 431 (3.46), 696 (2.34); MALDI-TOF MS *m/z* calcd for $C_{69}H_6O_2$ $[M]^+$ 866.0362, found 866.0392.

1.14. Synthesis of Compound 2h

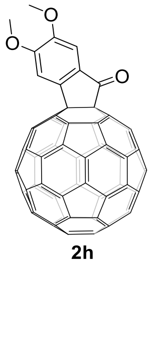

2h

Compound **1h** (13.5 mg, 0.015 mmol) was electroreduced by CPE at –1.36 V vs SCE, then the dianion $\mathbf{1h}^{2-}$ reacted with AcOH (8.6 μL, 0.15 mmol). After being stirred at room temperature for 0.5 h, the resulting mixture was directly filtered through a silica gel (200–300 mesh) plug with CS_2/EA (5:1 v/v) to remove the supporting electrolyte and insoluble materials, and then evaporated in vacuo to remove the solvent. Next, the residue was further separated on a silica gel column (300–400 mesh) with CS_2/CH_2Cl_2 (1:1 v/v) as the eluent to afford product **2h** (11.3 mg, 85%) as an amorphous brown solid along. ^{1}H NMR (400 MHz, 1:1 $CS_2/CDCl_3$) δ 7.79 (s, 1H), 7.73 (s, 1H), 4.15 (s, 3H), 4.13 (s, 3H); ^{13}C NMR spectrum with good signal/noise ratio could not be obtained due to its limited solubility; FT-IR ν/cm^{-1} (KBr) 2924, 1710, 1589, 1498, 1439, 1292, 1224, 1187, 1142, 1110, 1017, 861, 586, 558, 524; UV-vis ($CHCl_3$) λ_{max}/nm (log ε) 256 (5.02), 308 (4.62), 432 (3.42), 699 (2.22); MALDI-TOF MS *m/z* calcd for $C_{69}H_8O_3$ $[M]^+$ 884.0468, found 884.0466.

1.15. Synthesis of Compound 2i

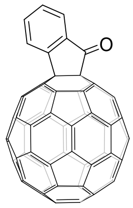

2i

Compound **1i** (12.6 mg, 0.015 mmol) was electroreduced by CPE at –1.36 V vs SCE, then the dianion $\mathbf{1i}^{2-}$ reacted with AcOH (8.6 μL, 0.15 mmol). After being stirred at room temperature for 0.5 h, the resulting mixture was directly filtered through a silica gel (200–300 mesh) plug with CS_2/CH_2Cl_2 (1:1 v/v) to remove the supporting electrolyte and insoluble materials, and then evaporated in vacuo to remove the solvent. Next, the residue was further separated on a silica gel column (300–400 mesh) with CS_2 as the eluent to afford product **2i** (11.1 mg, 90%) as an amorphous brown solid along. ^{1}H NMR (400 MHz, 1:1 $CS_2/CDCl_3$) δ 8.50 (d, J = 7.8 Hz, 1H), 8.43 (d, J = 7.7 Hz, 1H), 8.07 (td, J = 7.7, 1.2 Hz, 1H), 7.86 (td, J = 7.5, 0.7 Hz, 1H); ^{13}C NMR (100 MHz, 1:1 $CS_2/CDCl_3$ with $Cr(acac)_3$ as relaxation reagent) (all 2C unless indicated) δ 197.99 (1C, *C=O*), 156.11, 153.61, 153.22, 147.47, 147.22 (1C), 146.95 (1C), 146.17 (4C), 145.94, 145.87, 145.62, 145.44, 145.36, 145.29, 145.19, 145.17, 144.44, 144.07, 142.94, 142.56, 142.51, 142.21, 141.94, 141.90, 141.89, 141.69, 141.50, 140.44, 140.37, 136.90 (1C, aryl *C*), 135.33 (1C, aryl *C*), 135.25, 134.07 (1C, aryl *C*), 130.08 (1C, aryl *C*), 127.24 (1C, aryl *C*), 126.62 (1C, aryl *C*), 78.54 (1C, sp^3-*C* of C_{60}), 70.72 (1C, sp^3-*C* of C_{60}); FT-IR ν/cm^{-1} (KBr) 2918, 2849, 1721, 1589, 1456, 1426, 1276, 1225, 1182, 1004, 849, 763, 594, 572, 544, 525; UV-vis ($CHCl_3$) λ_{max}/nm (log ε) 263 (4.88), 305 (4.54), 431 (3.48), 699 (2.39); MALDI-TOF MS *m/z* calcd for $C_{67}H_4O$ $[M]^+$ 824.0257, found 824.0282.

2. Single-Crystal X-Ray Crystallography

Black block crystals of **2f** were obtained by slow evaporation of a saturated solution in toluene at room temperature. Single-crystal X-ray diffraction data were collected on a diffractometer (Gemini S Ultra, Agilent Technologies) equipped with a CCD area detector using graphite-monochromated Cu Kα radiation (λ = 1.54184 Å) in the scan range 8.90° < 2θ < 140.22°. The structure was solved with direct methods using SHELXS-97 and refined with full-matrix least-squares refinement using the SHELXL-97 program within OLEX2. Crystallographic data have been deposited in the Cambridge Crystallographic Data Centre as deposition number CCDC 1856172.

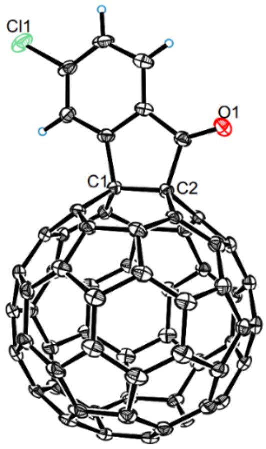

Figure S1. ORTEP diagram for one enantiomer of **2f** with thermal ellipsoids shown at 50% probability. The toluene molecule is omitted for clarity.

Table S1. Crystal Data and Structure Refinement for 2f

Identification code	CCDC 1856172
Empirical formula	$C_{67}H_3ClO$
Formula weight	859.14
Temperature/K	290(2)
Crystal system	monoclinic
Space group	$P2_1$
a/Å	10.1607(11)
b/Å	9.8856(11)
c/Å	16.4378(16)
α/°	90
β/°	102.188(10)
γ/°	90
Volume/Å^3	1613.9(3)
Z	2
ρ_{calc} g/cm^3	1.768
μ/mm^{-1}	1.558
F(000)	860.0
Crystal size/mm^3	0.350 × 0.310 × 0.200
Radiation	CuKα (λ = 1.54184)
2Θ range for data collection/°	8.904 to 140.22
Index ranges	$-11 \le h \le 12, -11 \le k \le 7, -19 \le l \le 18$
Reflections collected	6097
Independent reflections	4132 [R_{int} = 0.0375, R_{sigma} = 0.0743]
Data/restraints/parameters	4132/1/622
Goodness-of-fit on F^2	1.089
Final R indexes [I>=2σ (I)]	R_1 = 0.0714, wR_2 = 0.2020
Final R indexes [all data]	R_1 = 0.0880, wR_2 = 0.2230
Largest diff. peak/hole / e Å^{-3}	0.50/-0.37
Flack parameter	0.25(3)

3. Mechanism Studies

3.1. Electrochemical Reduction of 1a in the Presence of One Equiv of TFA

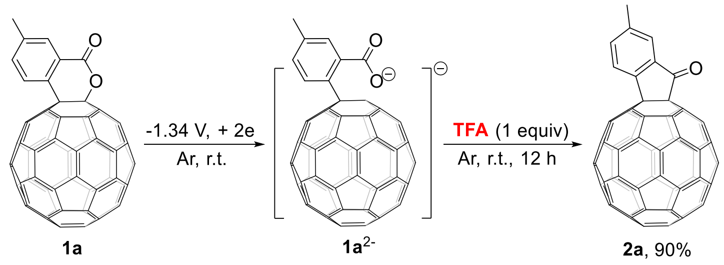

Compound **1a** (12.8 mg, 0.015 mmol) was electroreduced by CPE at –1.34 V vs SCE, then the dianion **1a**$^{2-}$ reacted with trifluoroacetic acid (TFA) (1.1 µL, 0.015 mmol). After being stirred at room temperature for 12 h, the resulting mixture was directly filtered through a silica gel (200–300 mesh) plug with CS_2/CH_2Cl_2 (1:1 v/v) to remove the supporting electrolyte and insoluble materials, and then evaporated in vacuo to remove the solvent. Next, the residue was further separated on a silica gel column (300–400 mesh) with CS_2 as the eluent to afford product **2a** (11.4 mg, 90%).

3.2. Electrochemical Reduction of 1a in the Presence of Three Equiv of TFA

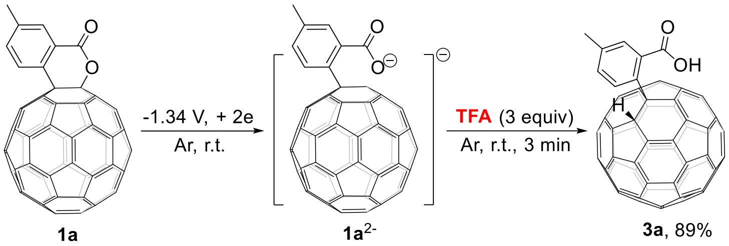

Compound **1a** (12.8 mg, 0.015 mmol) was electroreduced by CPE at –1.34 V vs SCE, then the dianion **1a**$^{2-}$ reacted with TFA (3.3 µL, 0.045 mmol). After being stirred at room temperature for 3 min, the resulting mixture was directly filtered through a neutral silica gel (200–300 mesh, pH: 6.5~7.5) plug with CS_2/EA (5:1 v/v) to remove the supporting electrolyte and insoluble materials, and then evaporated in vacuo to remove the solvent. Next, the residue was further separated on a neutral silica gel column (200–300 mesh, pH: 6.5~7.5) with CS_2/EA (5:1 v/v) as the eluent to afford product **3a** (11.4 mg, 89%) as an amorphous brown solid along.

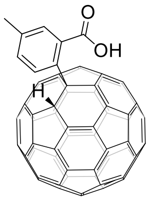

3a

Compound **3a**. ^{1}H NMR (400 MHz, 1:1 $CS_2/CDCl_3$) δ 8.50 (d, J = 8.1 Hz, 1H), 7.83 (s, 1H), 7.62 (d, J = 8.1 Hz, 1H), 6.89 (s, 1H), 2.56 (s, 3H); ^{13}C NMR (100 MHz, 1:1 $CS_2/CDCl_3$) (all 2C unless indicated) δ 174.74 (1C, *C*=*O*), 153.02 (4C), 147.46 (1C), 147.08 (1C), 146.87, 146.30, 146.23, 146.04 (6C), 145.70, 145.50, 145.33, 145.28, 145.22, 144.71, 144.34, 143.21, 142.47 (4C), 142.01, 141.98 (4C), 141.86, 141.56, 141.42, 140.93, 140.09, 139.70, 138.16, 136.71 (1C, aryl *C*), 132.95 (1C, aryl *C*), 131.84 (1C, aryl *C*), 131.75 (1C, aryl *C*), 131.48 (1C, aryl *C*), 126.33 (1C, aryl *C*), 67.29 (1C, sp^3-*C* of C_{60}), 61.84 (1C, sp^3-*C* of C_{60}), 20.97 (1C); FT-IR ν/cm^{-1} (KBr) 2916, 1696, 1509, 1420, 1256, 1189, 1074, 891, 822, 771, 657, 584, 524; UV-vis ($CHCl_3$) λ_{max}/nm (log ε) 257 (4.79), 313 (4.32), 432 (3.34), 697 (2.21). MALDI-TOF MS *m/z* calcd for $C_{68}H_8O_2$ [M]$^+$ 856.0519, found 856.0519.

3.3. Reaction of 3a with One Equiv of NaH

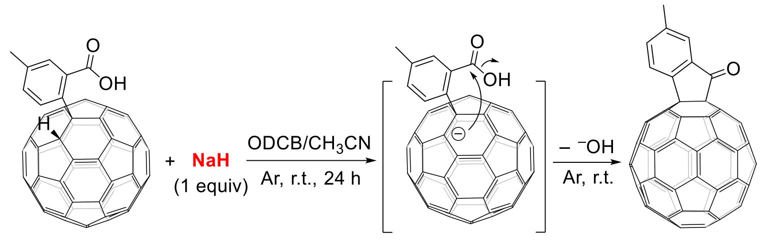

Product **3a** (6.8 mg, 0.008 mmol) and NaH (57–63% oil dispersion, 0.3 mg, 0.008 mmol) were added in a 15-mL reaction vessel containing a magnetic stirring bar. Then, the solid mixture was thoroughly degassed through five freeze-pump-thaw cycles, and subsequently filled with argon. Next, the degassed ODCB (4.0 mL) and CH_3CN (1 mL) were added into the vessel. The resulting mixture was stirred vigorously at room temperature under an argon atmosphere. As time went by, the color of the solution changed from brownish to dark green. Later, the resulting mixture turned into brownish from dark green. After 24 h, the solvent was directly removed under vacuum. The residue was further separated on a silica gel column (300–400 mesh) with CS_2 as the eluent to afford product **2a** (4.7 mg, 71%).

3.4. Synthesis of 2a by the Reaction of $1a^{\cdot-}$ with AcOH

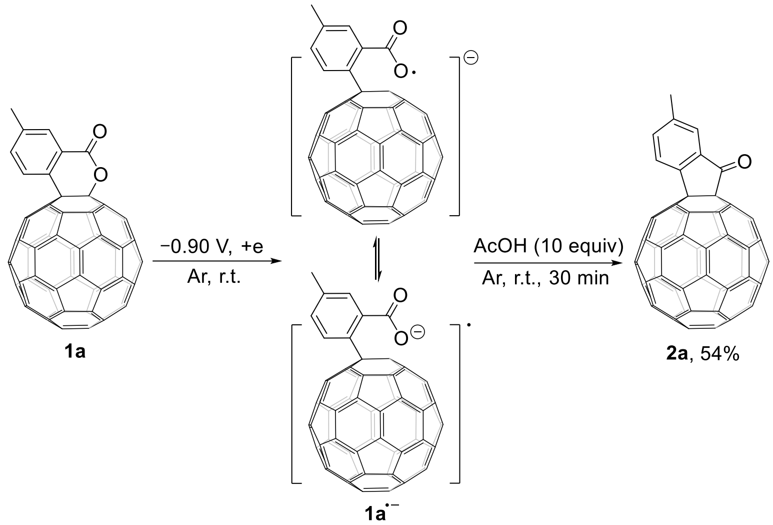

Compound **1a** (12.8 mg, 0.015 mmol) was electroreduced by CPE at –0.90 V vs SCE, then the radical anion **1a**$^{\cdot-}$ reacted with acetic acid (AcOH) (8.6 μL, 0.15 mmol). After being stirred at room temperature for 0.5 h, the resulting mixture was directly filtered through a silica gel (200–300 mesh) plug with CS_2/CH_2Cl_2 (1:1 v/v) to remove the supporting electrolyte and insoluble materials, and then evaporated in vacuo to remove the solvent. Next, the residue was further separated on a silica gel column (300–400 mesh) with CS_2 as the eluent to afford recovered **1a** (5.0 mg, 39%) and product **2a** (6.8 mg, 54%).

4. NMR Spectra of Compounds 2a–i and 3a

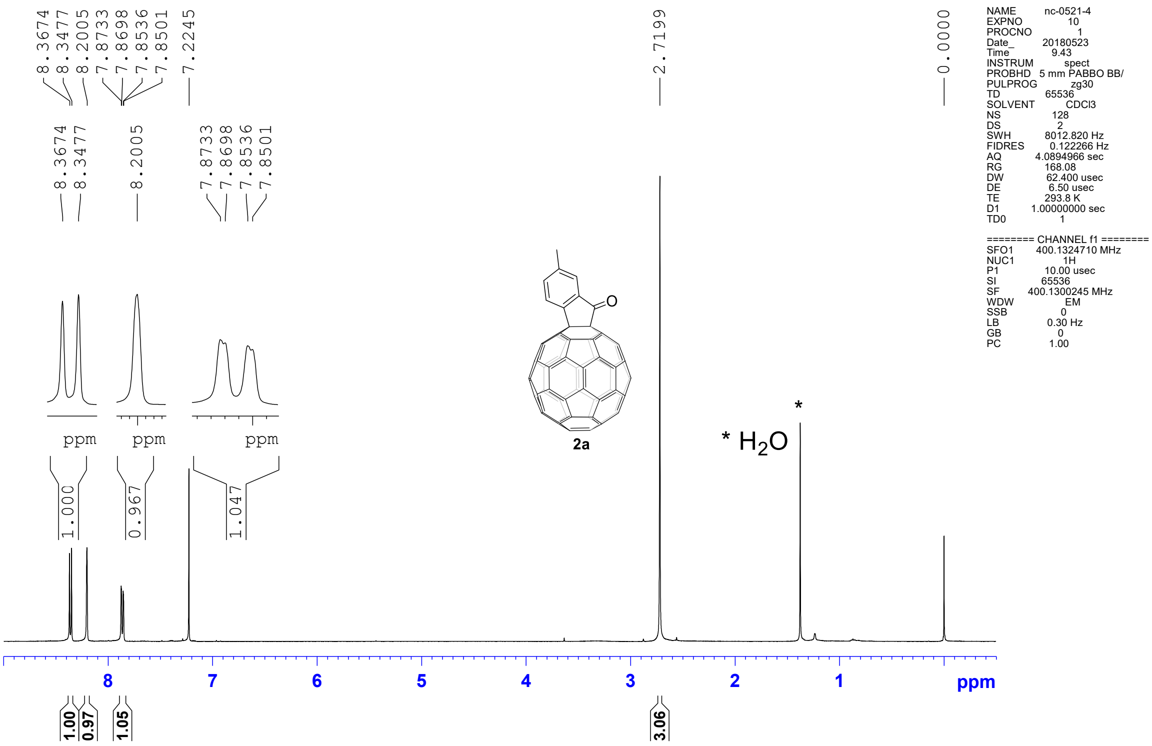

Figure S2. ^{1}H NMR (400 MHz, 1:1 $CS_2/CDCl_3$) of compound **2a**.

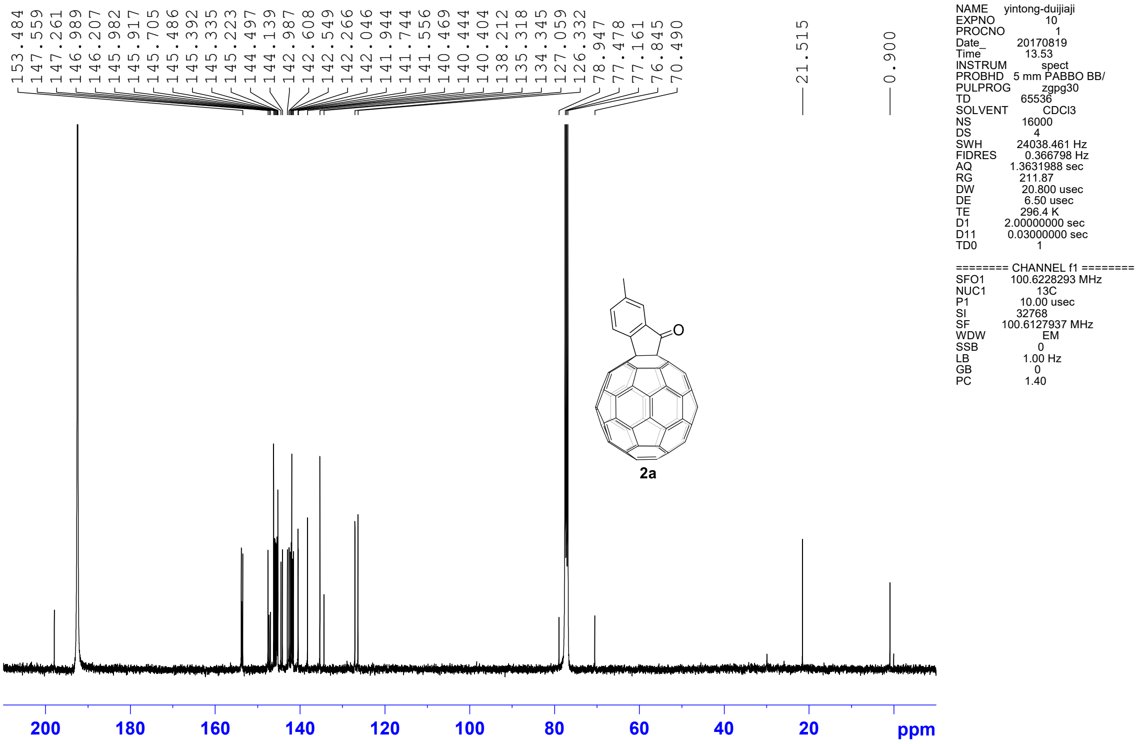

Figure S3. ^{13}C NMR (100 MHz, 1:1 $CS_2/CDCl_3$ with $Cr(acac)_3$ as a relaxation agent) of compound **2a**.

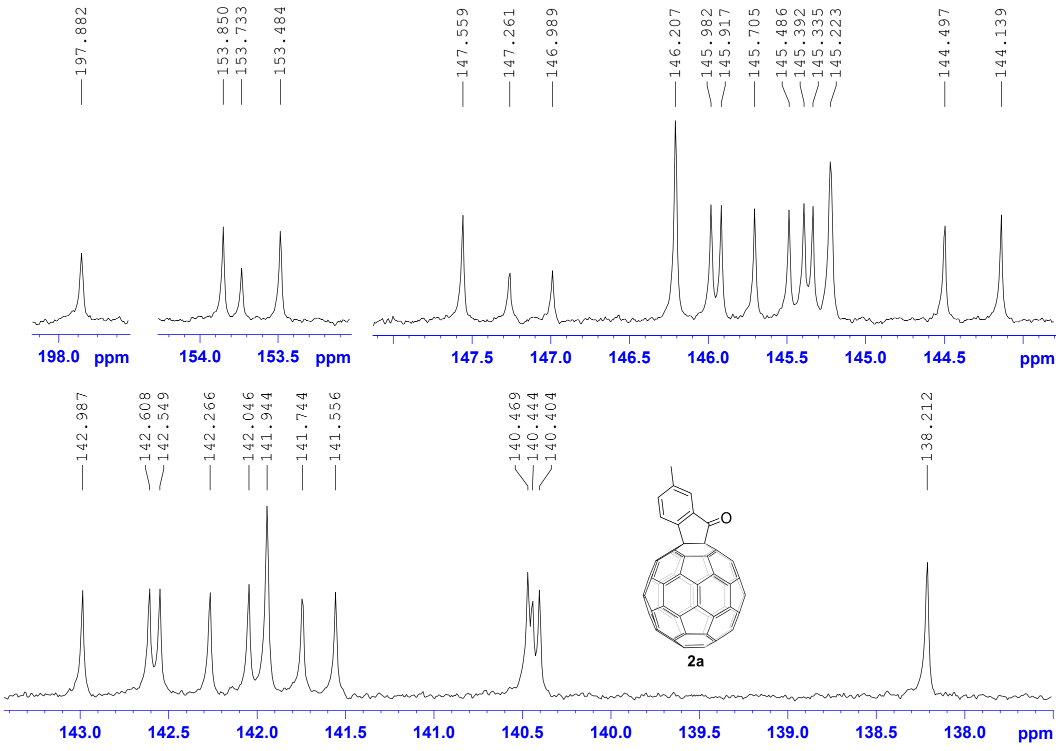

Figure S4. Expanded ^{13}C NMR (100 MHz, 1:1 $CS_2/CDCl_3$ with $Cr(acac)_3$ as a relaxation agent) of compound **2a**.

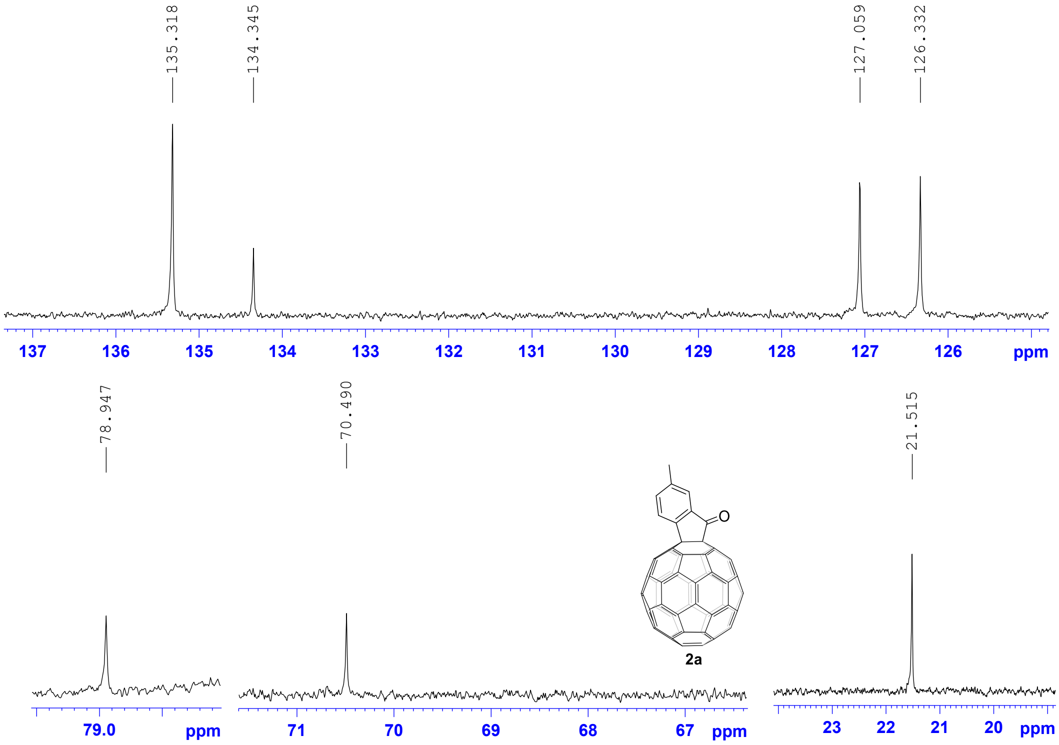

Figure S5. Expanded ^{13}C NMR (100 MHz, 1:1 $CS_2/CDCl_3$ with $Cr(acac)_3$ as a relaxation agent) of compound **2a**.

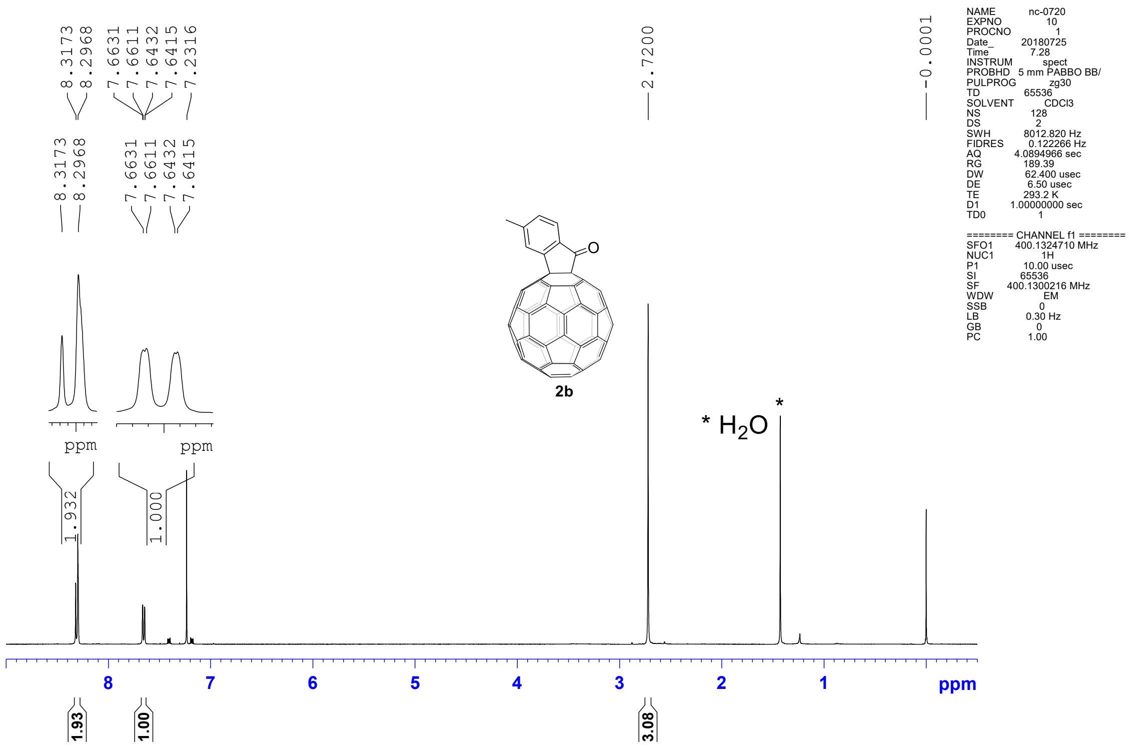

Figure S6. ^{1}H NMR (400 MHz, 1:1 $CS_2/CDCl_3$) of compound **2b**.

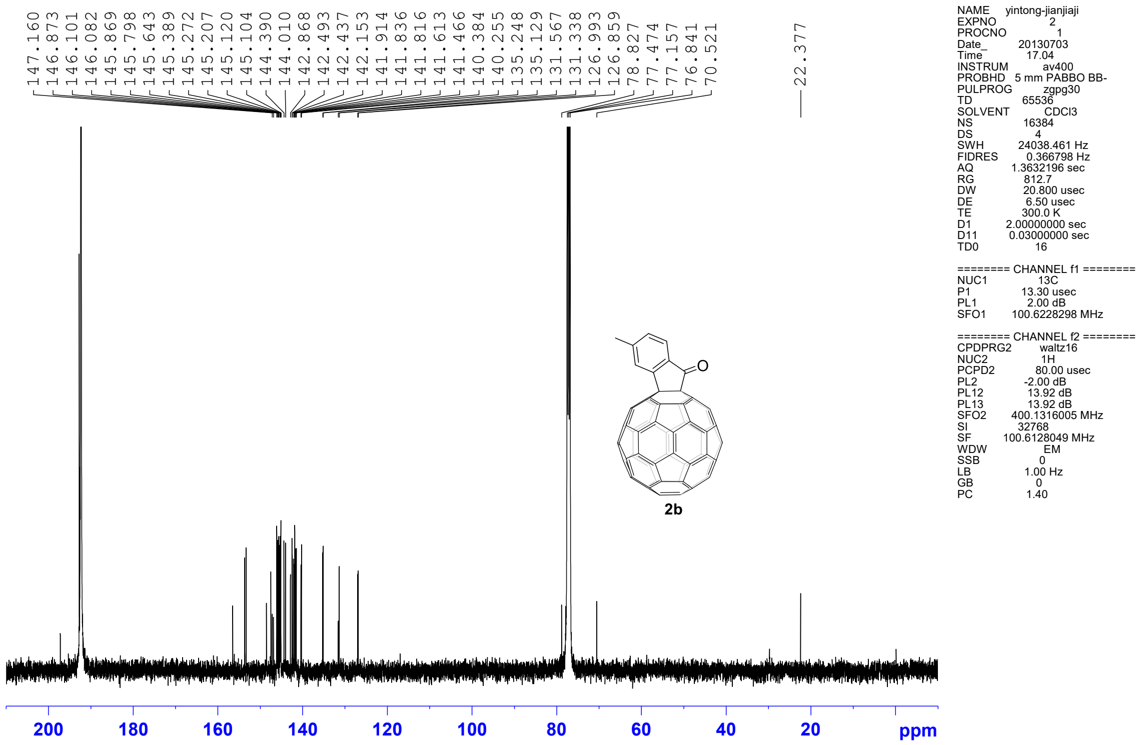

Figure S7. ^{13}C NMR (100 MHz, 1:1 $CS_2/CDCl_3$ with $Cr(acac)_3$ as a relaxation agent) of compound **2b**.

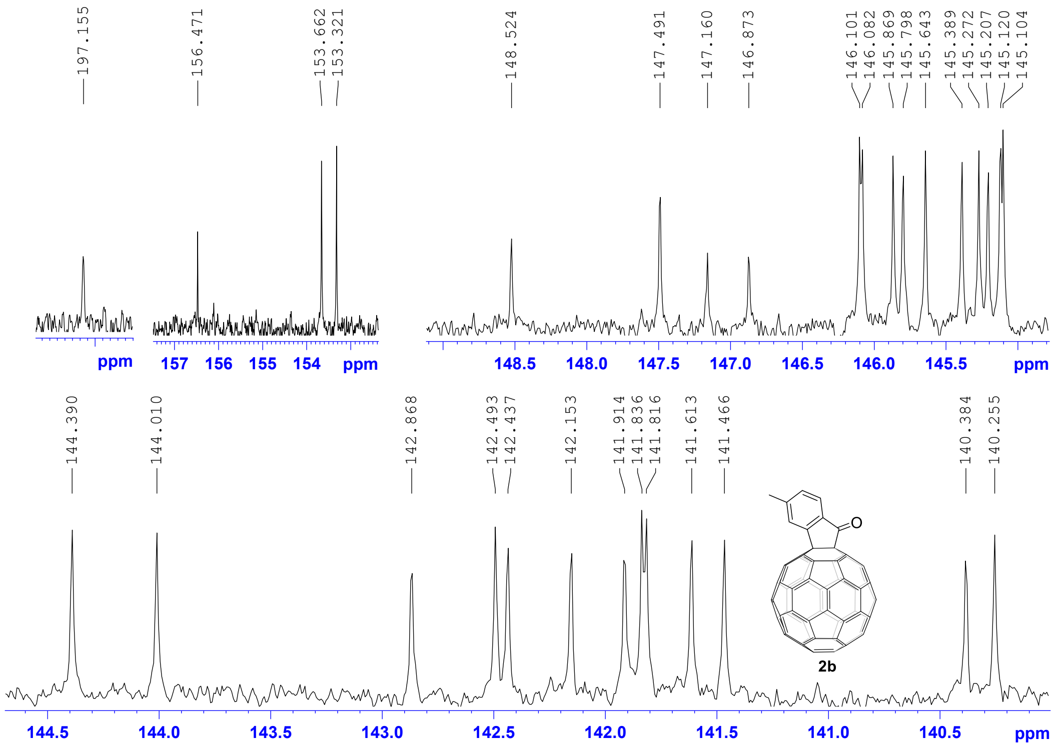

Figure S8. Expanded ^{13}C NMR (100 MHz, 1:1 $CS_2/CDCl_3$ with $Cr(acac)_3$ as a relaxation agent) of compound **2b**.

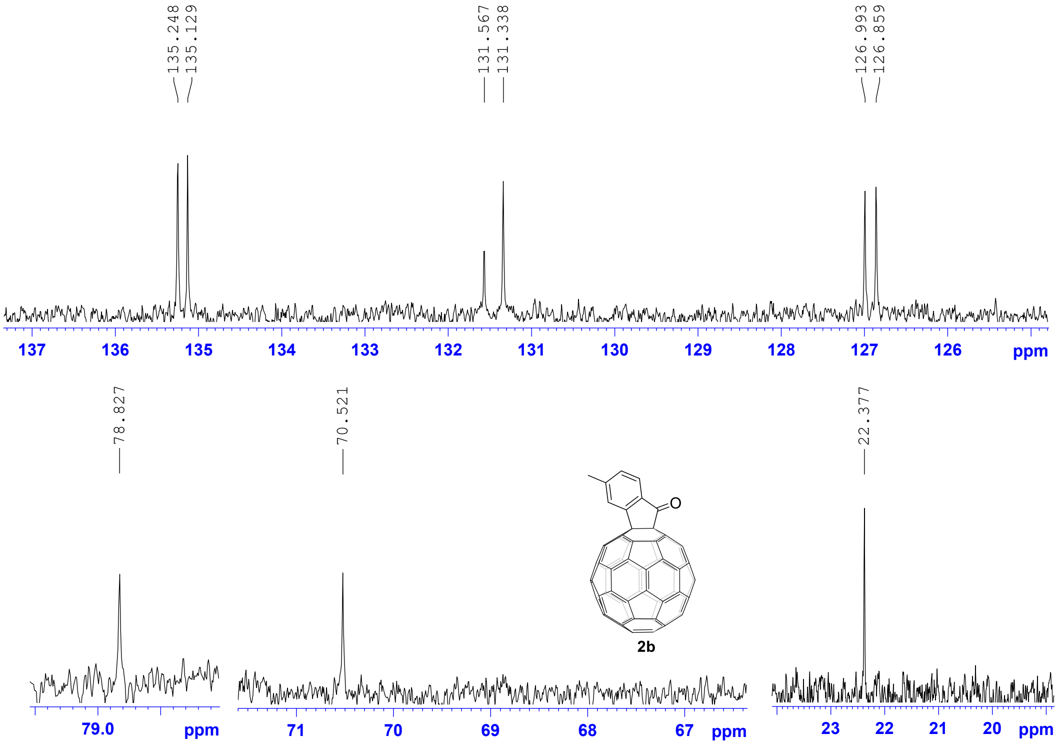

Figure S9. Expanded ^{13}C NMR (100 MHz, 1:1 $CS_2/CDCl_3$ with $Cr(acac)_3$ as a relaxation agent) of compound **2b**.

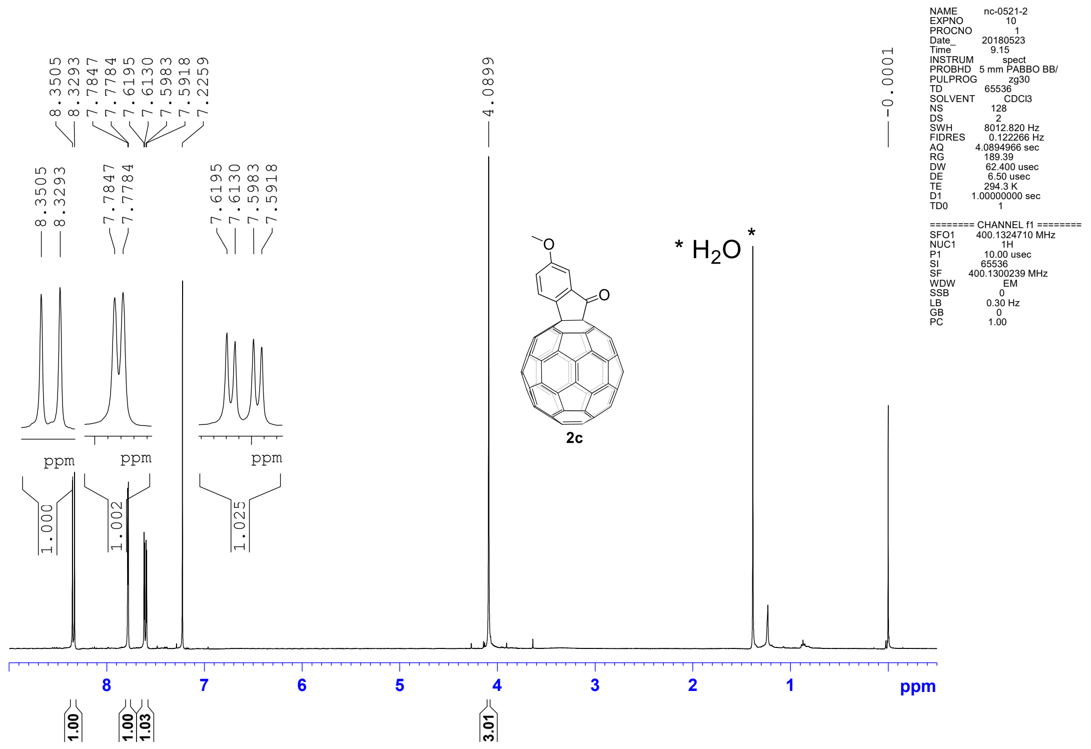

Figure S10. ^{1}H NMR (400 MHz, 1:1 CS_2/$CDCl_3$) of compound **2c**.

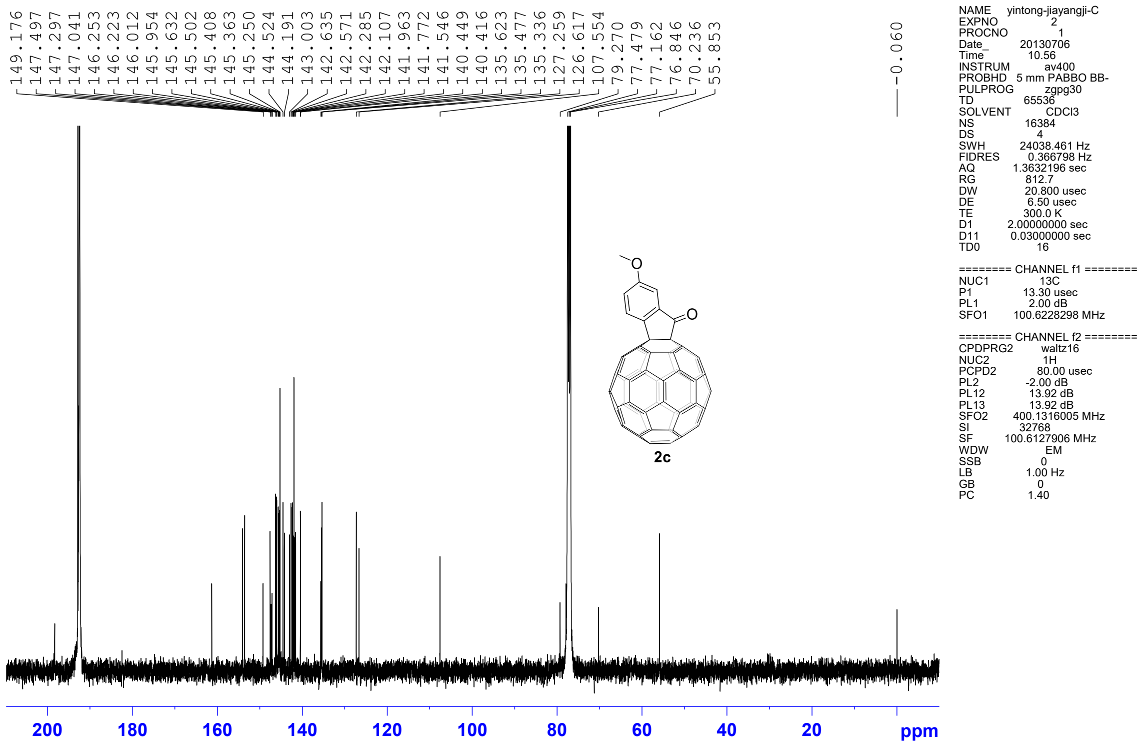

Figure S11. ^{13}C NMR (100 MHz, 1:1 CS_2/$CDCl_3$ with Cr(acac)$_3$ as a relaxation agent) of compound **2c**.

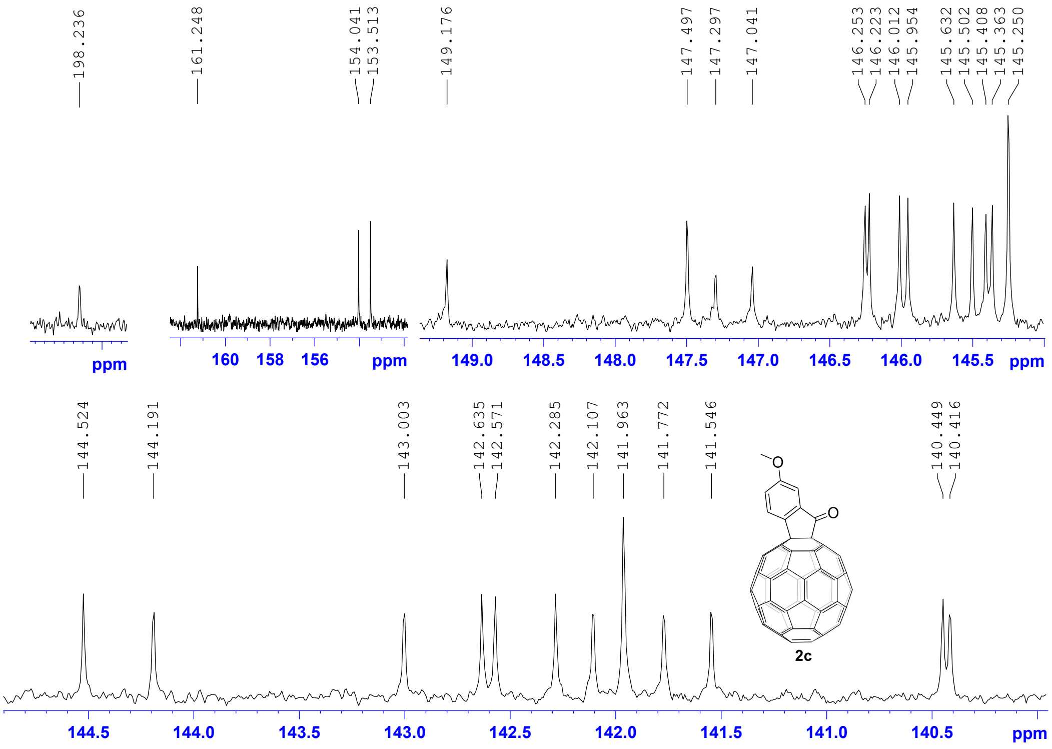

Figure S12. Expanded ^{13}C NMR (100 MHz, 1:1 $CS_2/CDCl_3$ with $Cr(acac)_3$ as a relaxation agent) of compound **2c**.

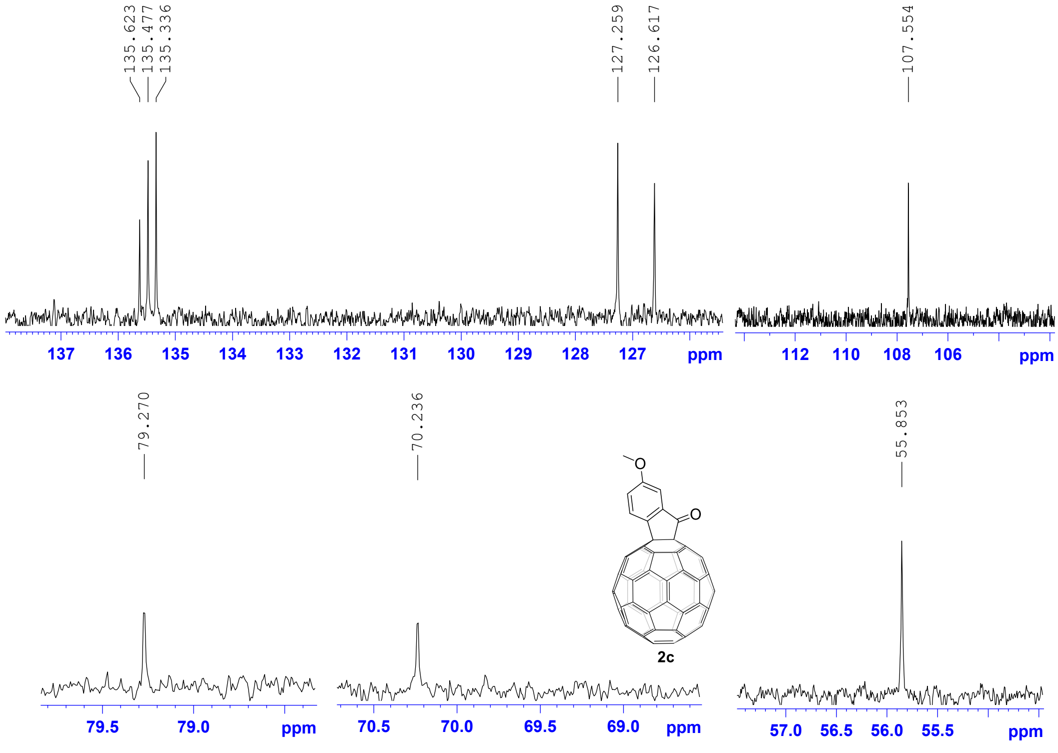

Figure S13. Expanded ^{13}C NMR (100 MHz, 1:1 $CS_2/CDCl_3$ with $Cr(acac)_3$ as a relaxation agent) of compound **2c**.

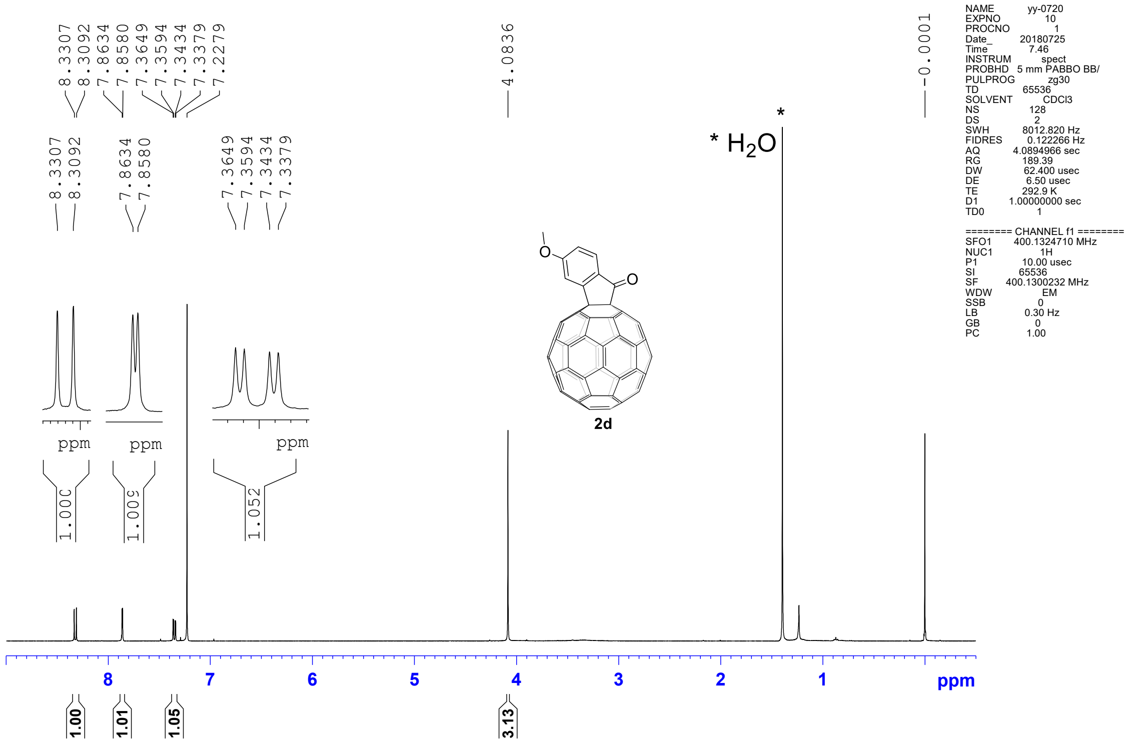

Figure S14. ^{1}H NMR (400 MHz, 1:1 $CS_2/CDCl_3$) of compound **2d**.

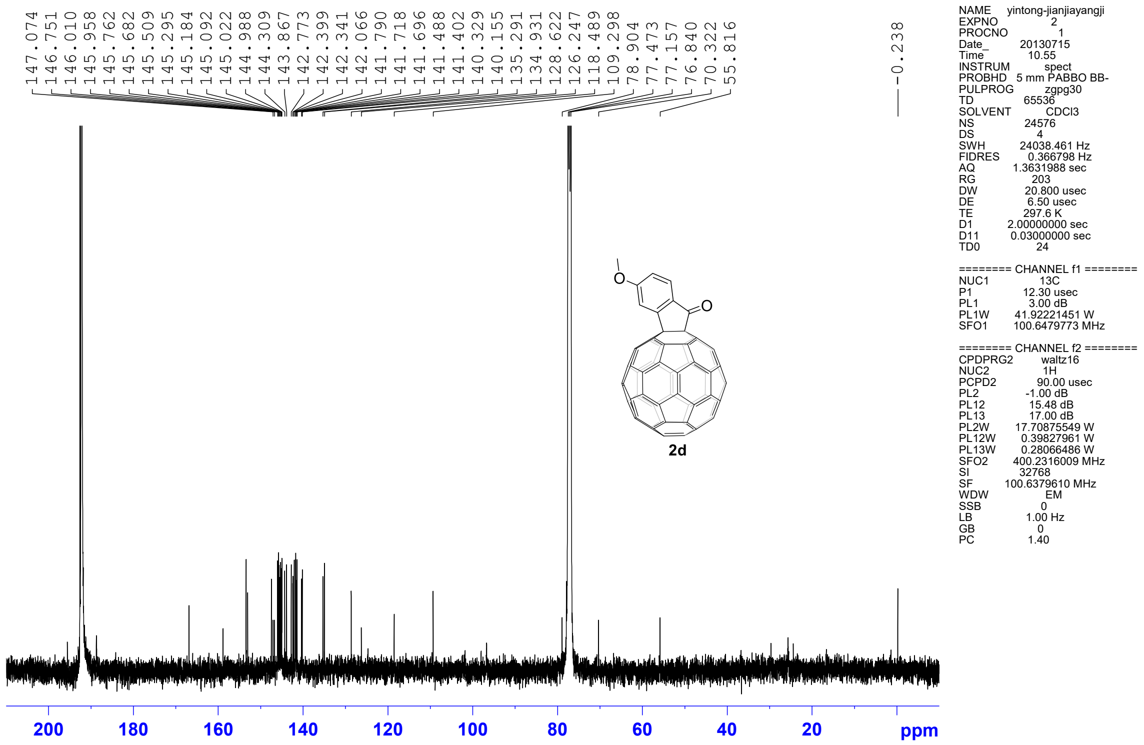

Figure S15. ^{13}C NMR (100 MHz, 1:1 $CS_2/CDCl_3$ with $Cr(acac)_3$ as a relaxation agent) of compound **2d**.

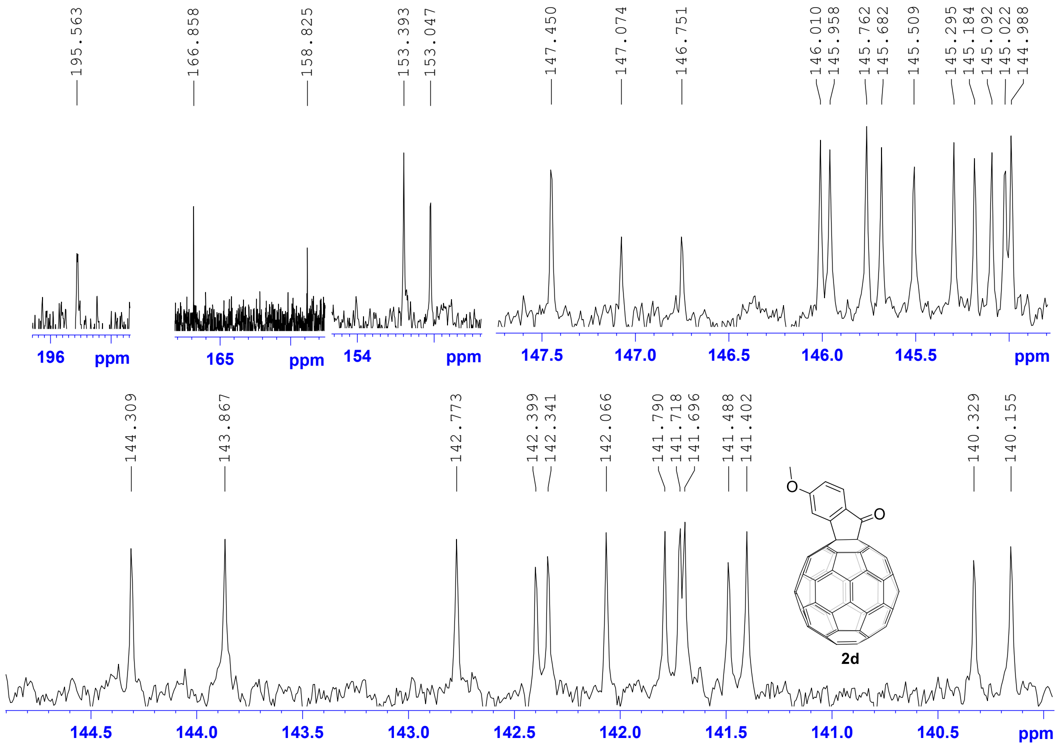

Figure S16. Expanded ^{13}C NMR (100 MHz, 1:1 $CS_2/CDCl_3$ with $Cr(acac)_3$ as a relaxation agent) of compound **2d**.

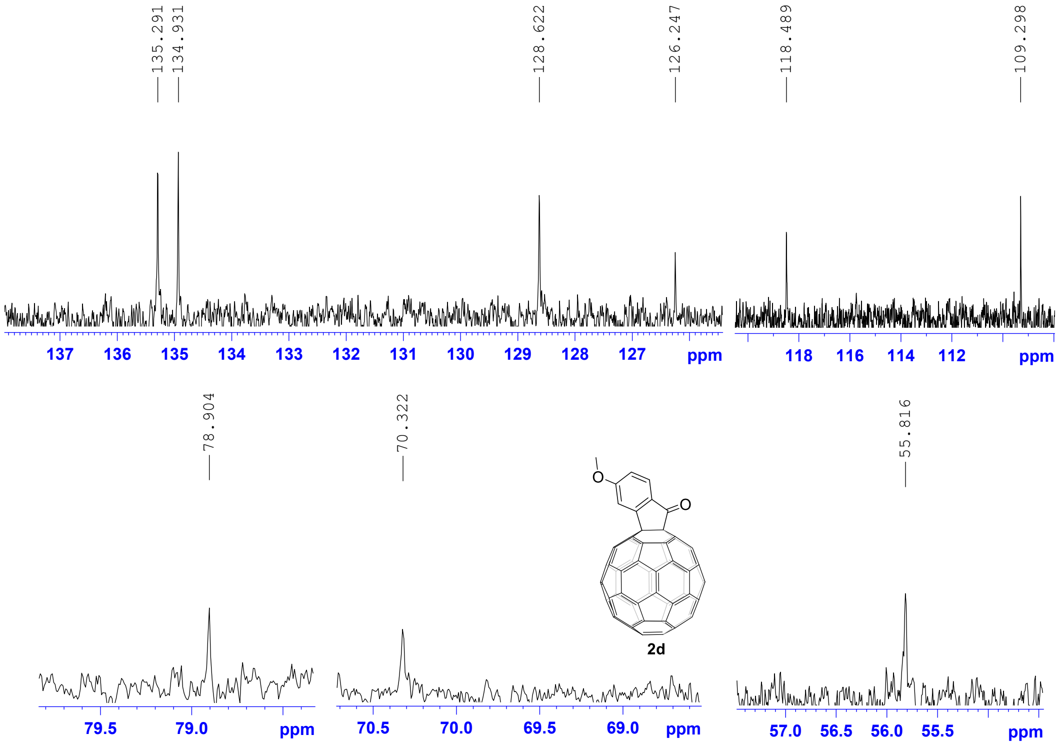

Figure S17. Expanded ^{13}C NMR (100 MHz, 1:1 $CS_2/CDCl_3$ with $Cr(acac)_3$ as a relaxation agent) of compound **2d**.

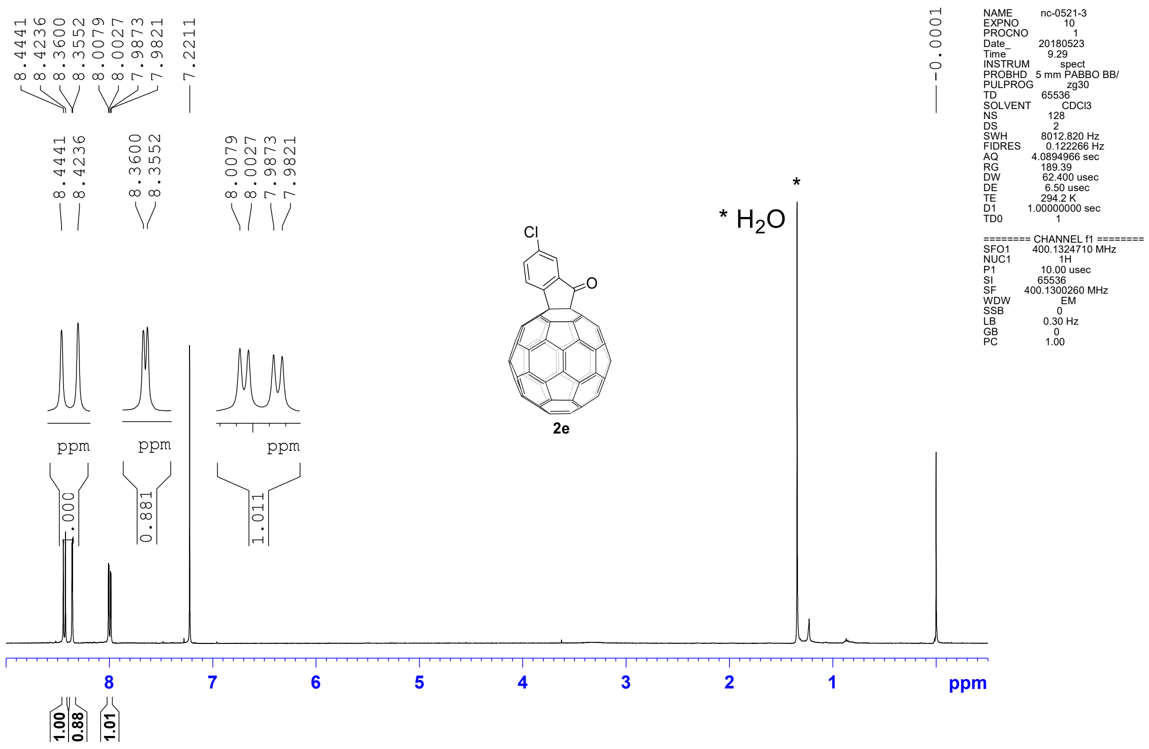

Figure S18. ^{1}H NMR (400 MHz, 1:1 CS_2/$CDCl_3$) of compound **2e**.

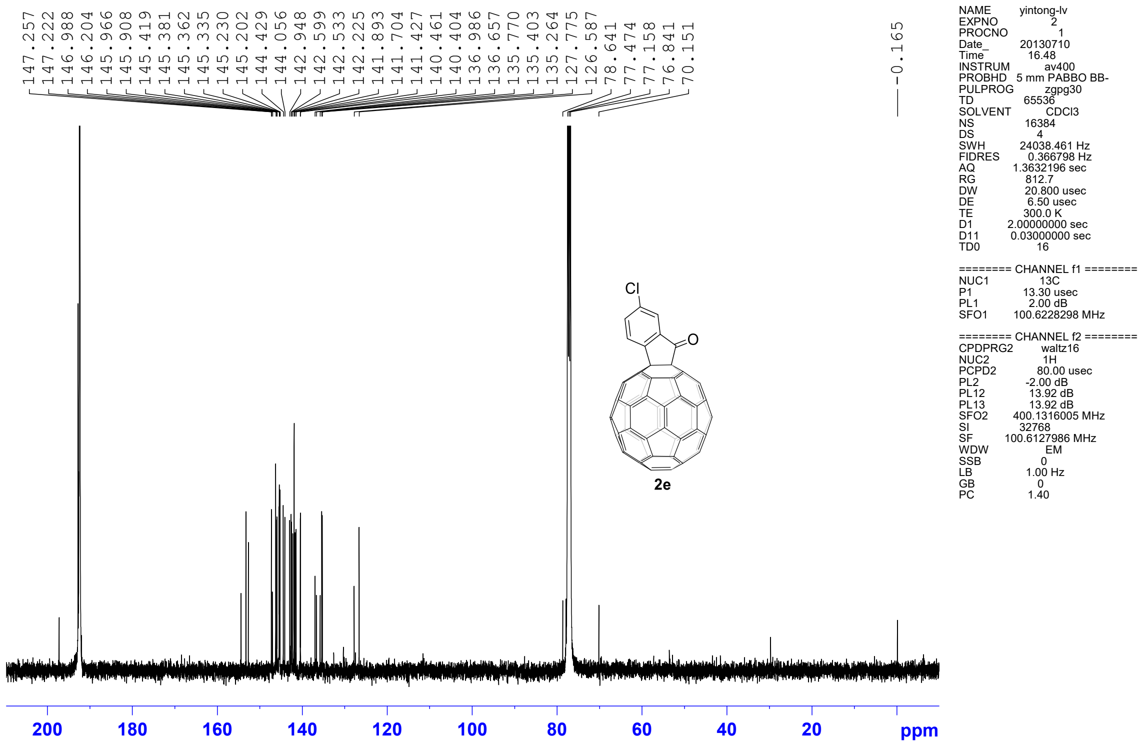

Figure S19. ^{13}C NMR (100 MHz, 1:1 CS_2/$CDCl_3$ with $Cr(acac)_3$ as a relaxation agent) of compound **2e**.

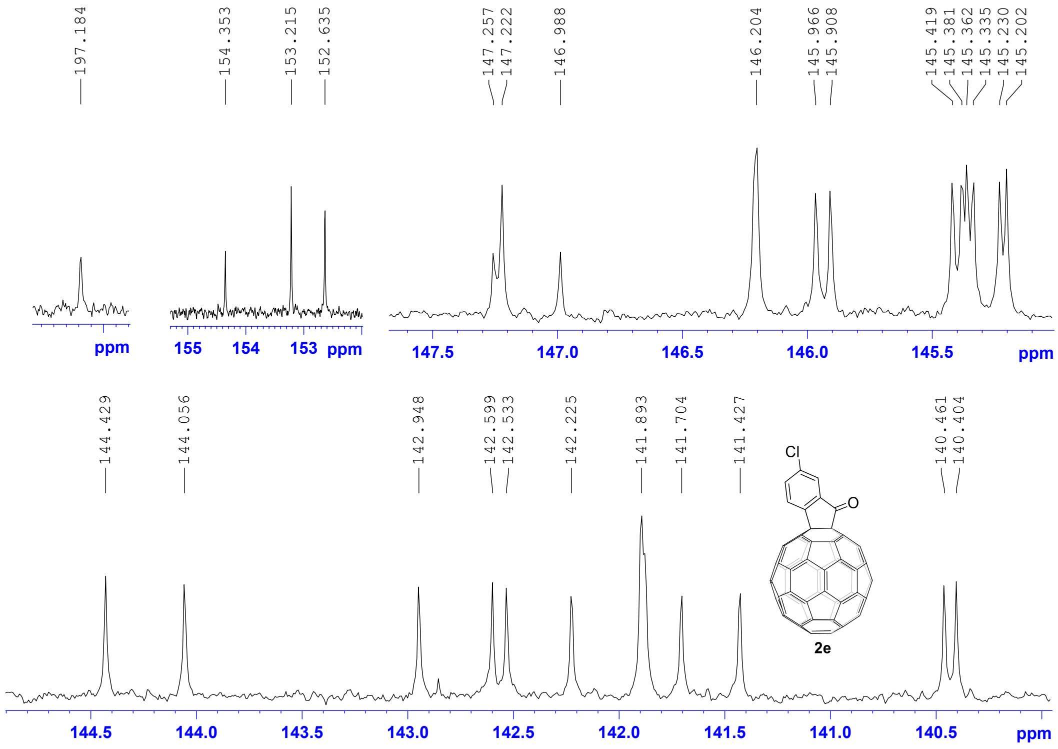

Figure S20. Expanded ^{13}C NMR (100 MHz, 1:1 $CS_2/CDCl_3$ with $Cr(acac)_3$ as a relaxation agent) of compound **2e**.

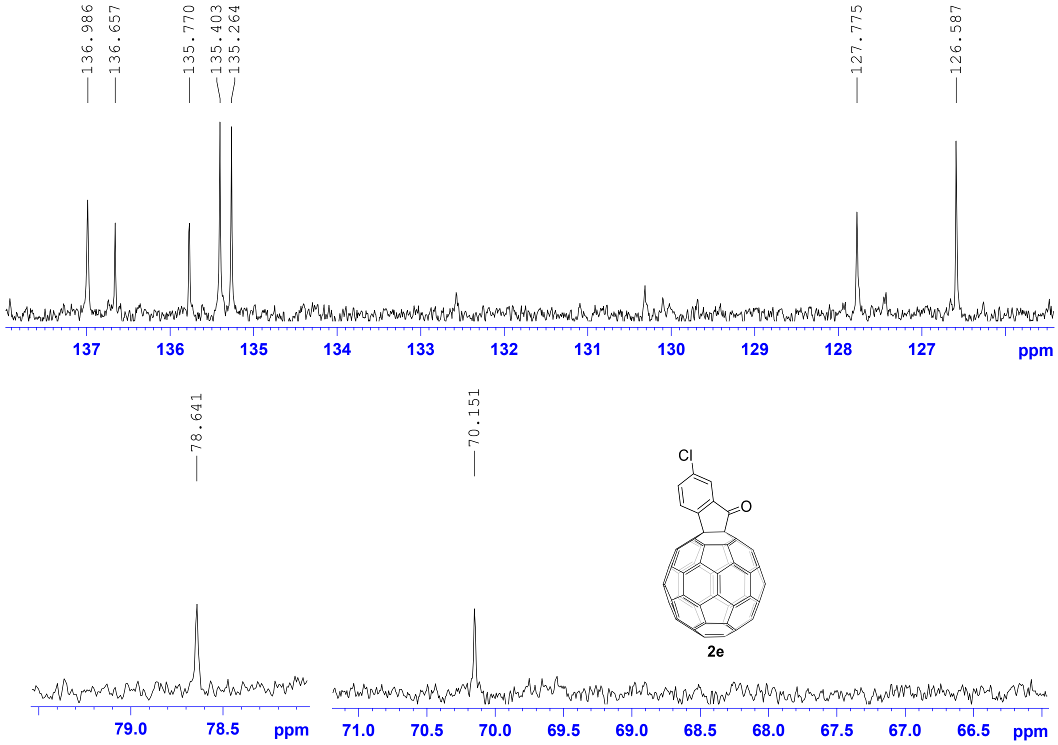

Figure S21. Expanded ^{13}C NMR (100 MHz, 1:1 $CS_2/CDCl_3$ with $Cr(acac)_3$ as a relaxation agent) of compound **2e**.

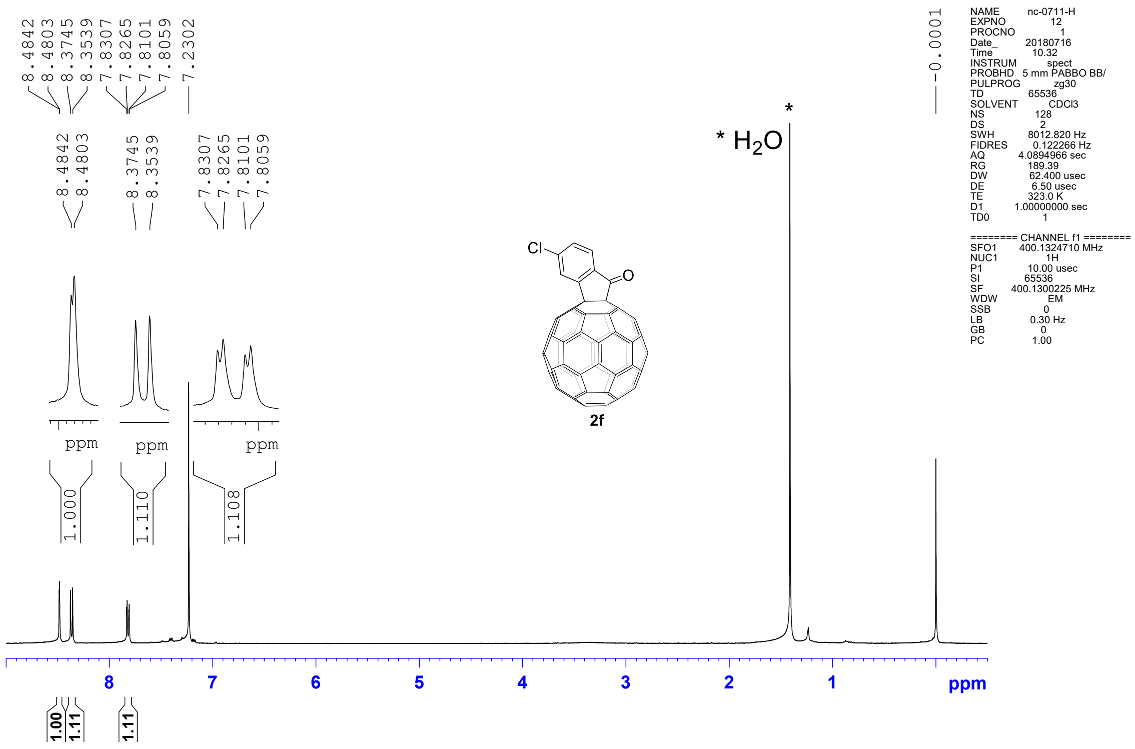

Figure S22. ^{1}H NMR (400 MHz, 1:1 CS_2/$CDCl_3$) of compound **2f**.

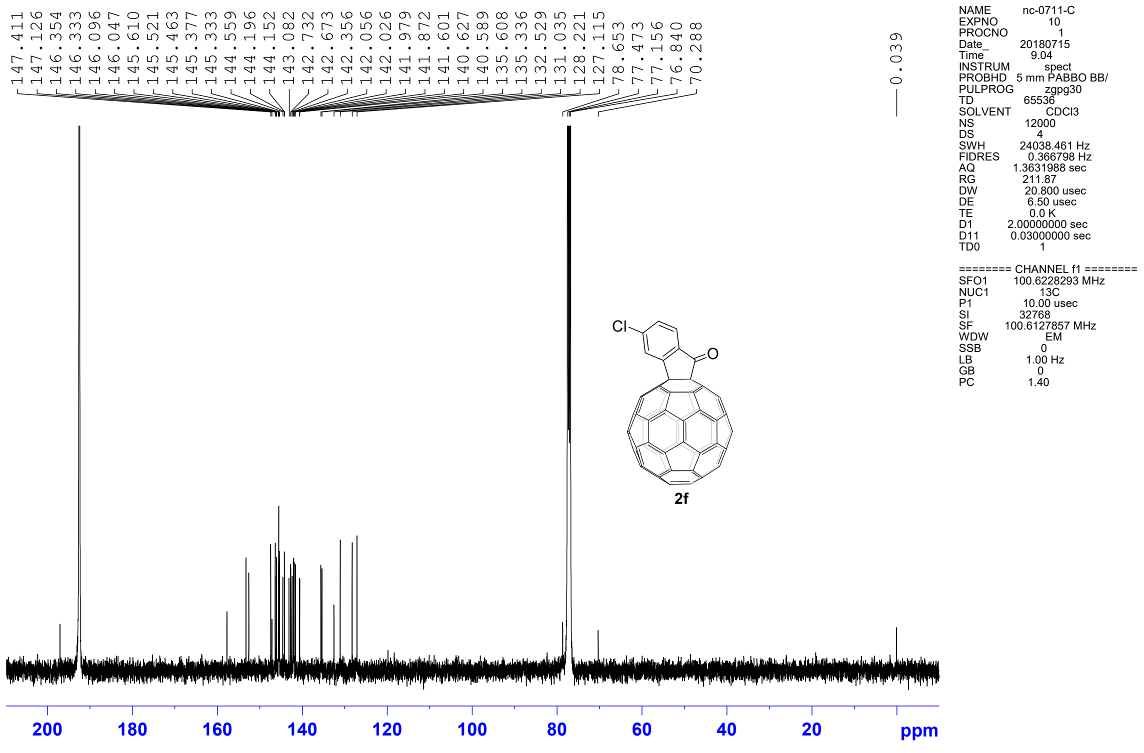

Figure S23. ^{13}C NMR (100 MHz, 1:1 CS_2/$CDCl_3$ with $Cr(acac)_3$ as a relaxation agent) of compound **2f**.

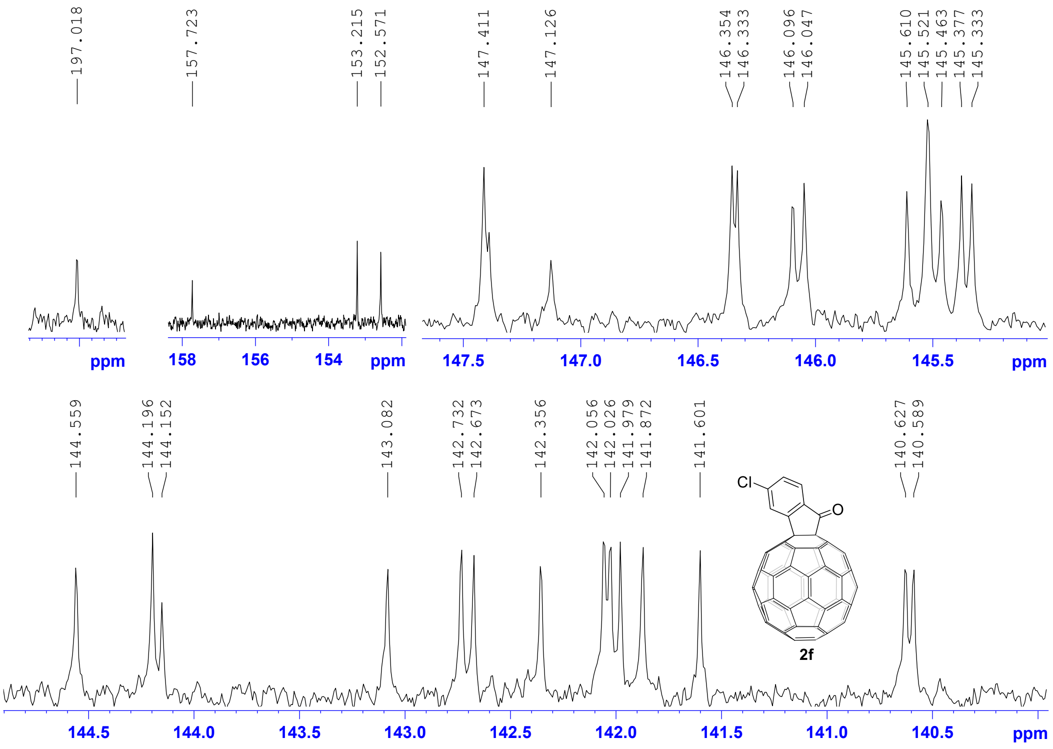

Figure S24. Expanded ^{13}C NMR (100 MHz, 1:1 $CS_2/CDCl_3$ with $Cr(acac)_3$ as a relaxation agent) of compound **2f**.

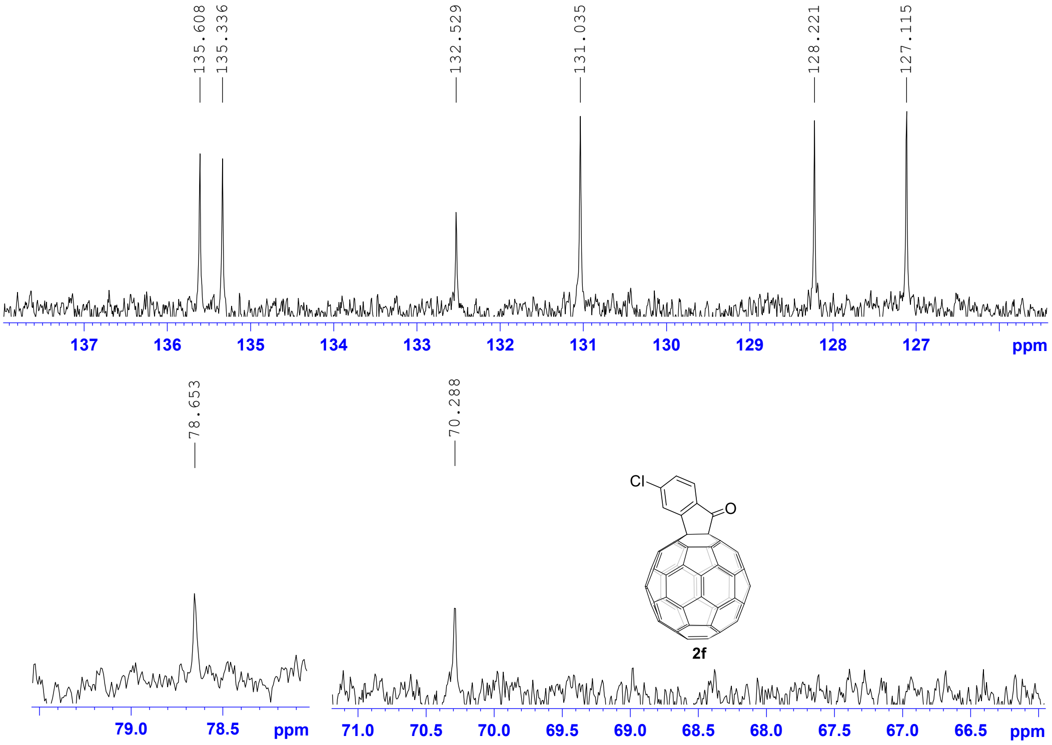

Figure S25. Expanded ^{13}C NMR (100 MHz, 1:1 $CS_2/CDCl_3$ with $Cr(acac)_3$ as a relaxation agent) of compound **2f**.

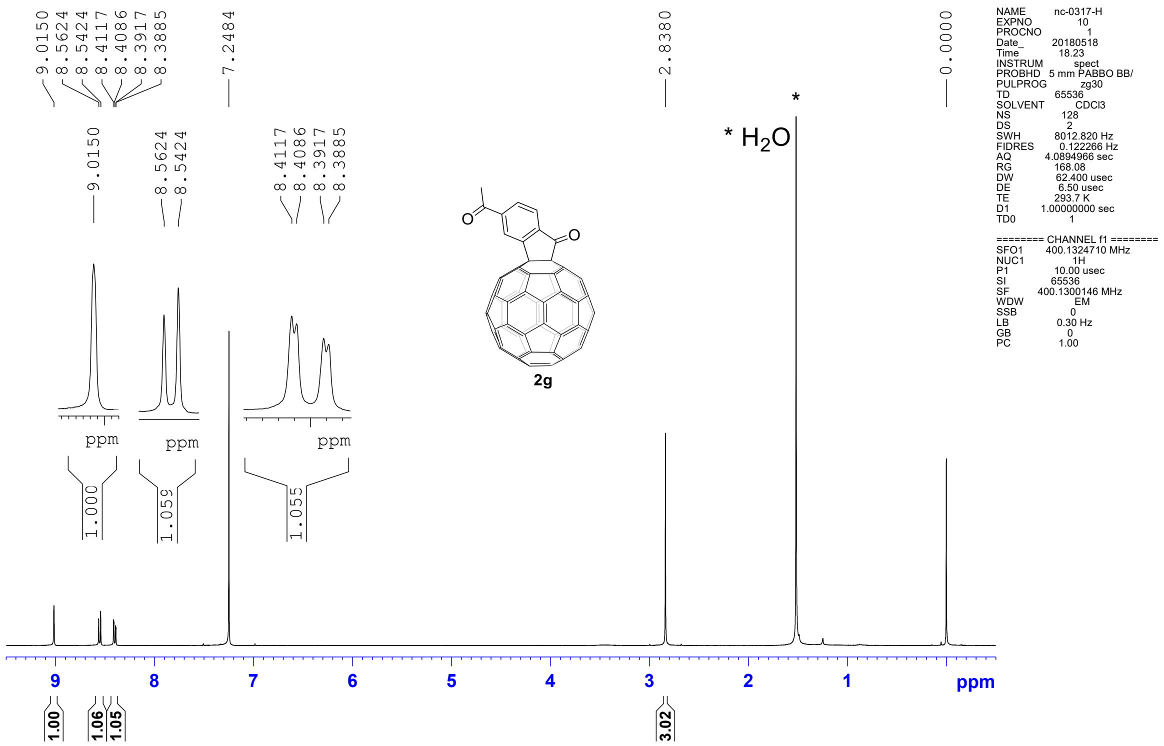

Figure S26. ^{1}H NMR (400 MHz, 1:1 CS_2/$CDCl_3$) of compound **2g**.

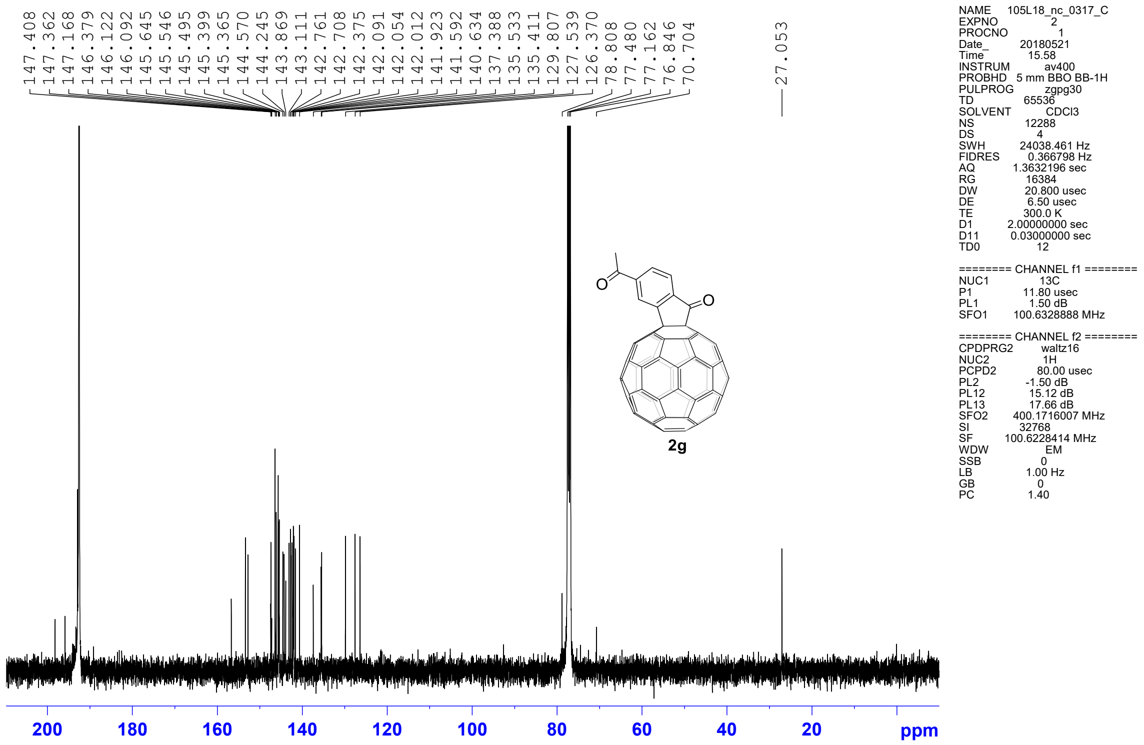

Figure S27. ^{13}C NMR (100 MHz, 1:1 CS_2/$CDCl_3$ with $Cr(acac)_3$ as a relaxation agent) of compound **2g**.

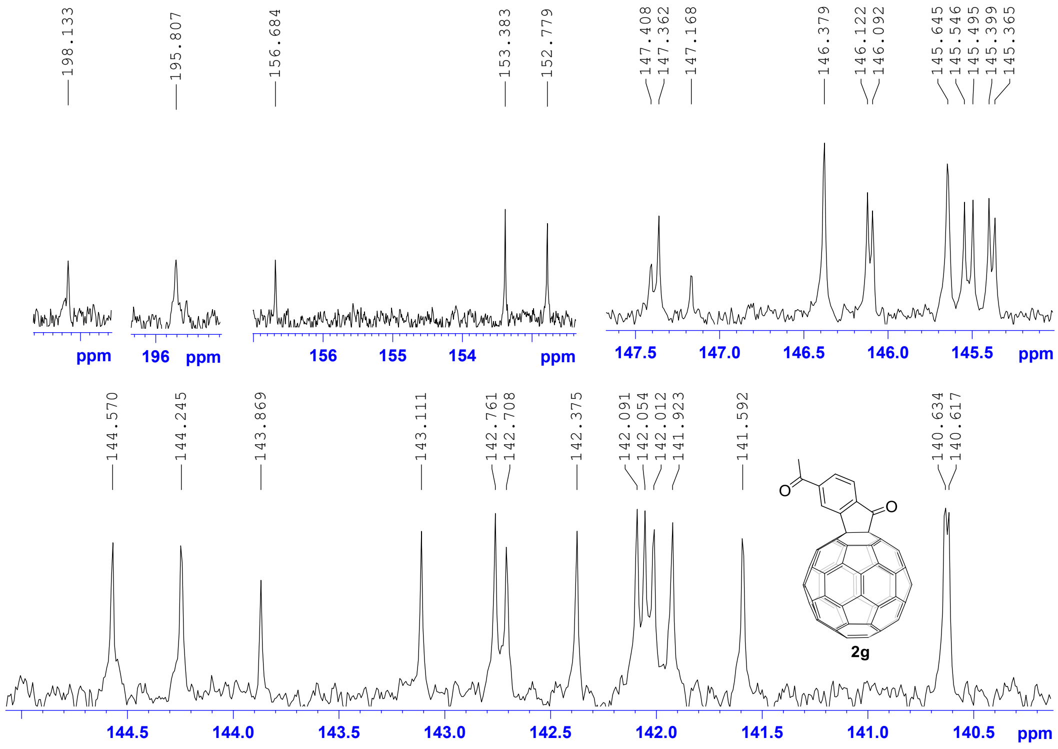

Figure S28. Expanded ^{13}C NMR (100 MHz, 1:1 CS_2/$CDCl_3$ with Cr(acac)$_3$ as a relaxation agent) of compound **2g**.

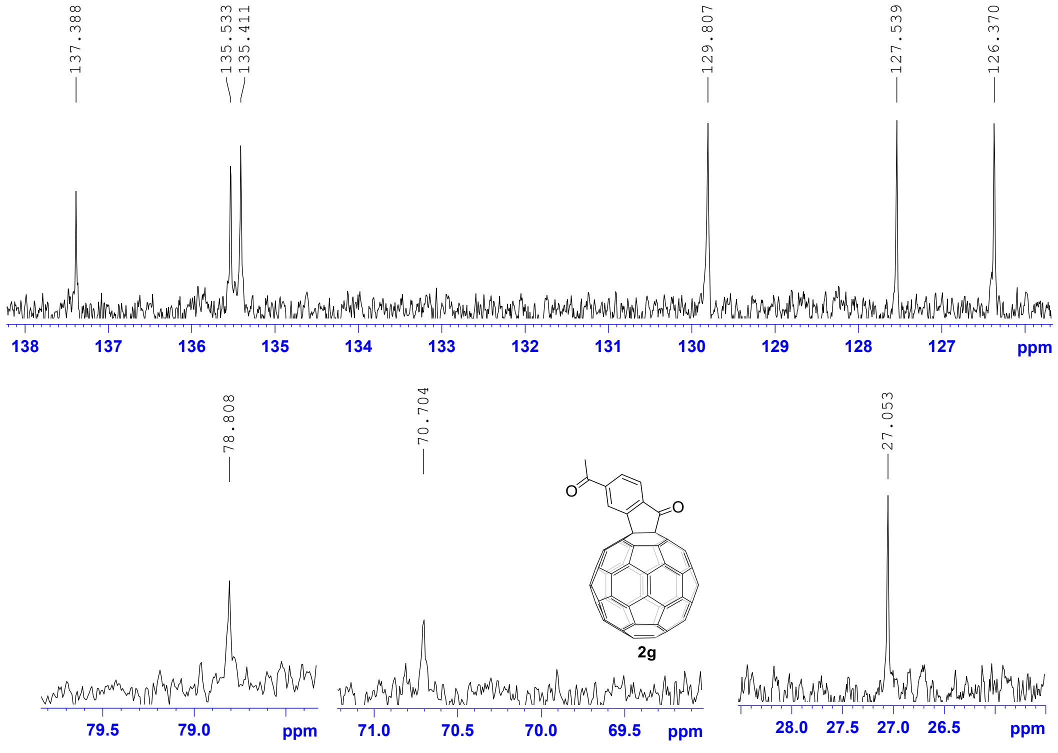

Figure S29. Expanded ^{13}C NMR (100 MHz, 1:1 CS_2/$CDCl_3$ with Cr(acac)$_3$ as a relaxation agent) of compound **2g**.

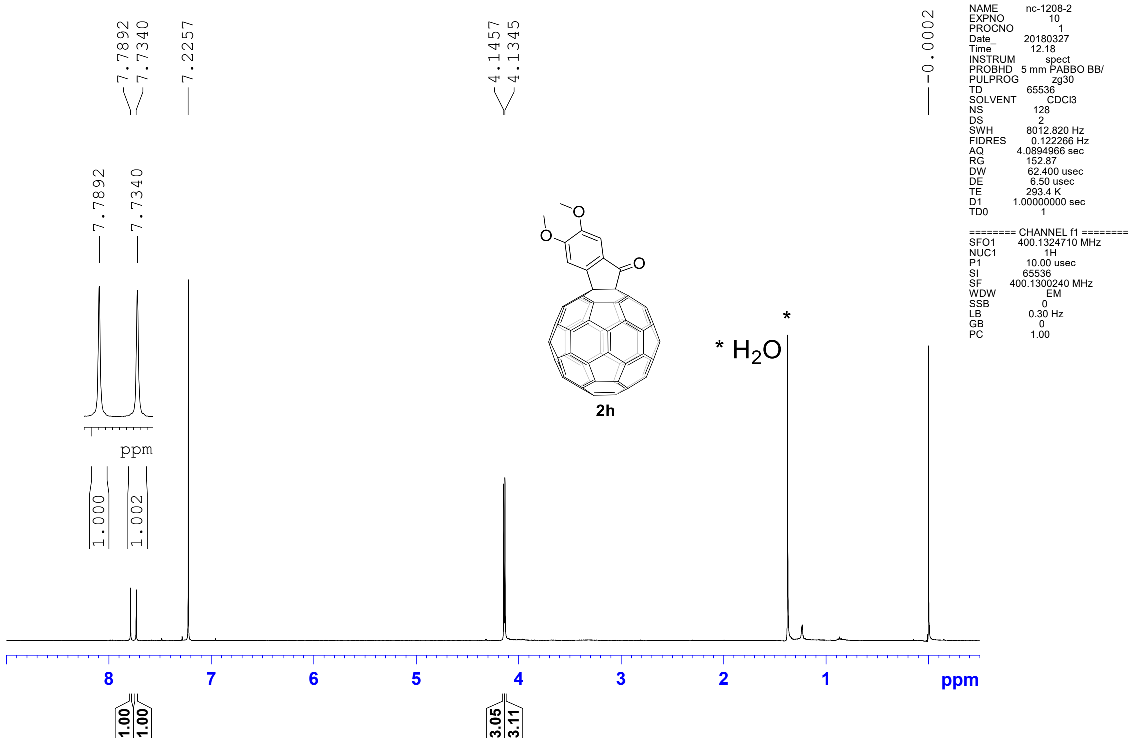

Figure S30. ^{1}H NMR (400 MHz, 1:1 $CS_2/CDCl_3$) of compound **2h**.

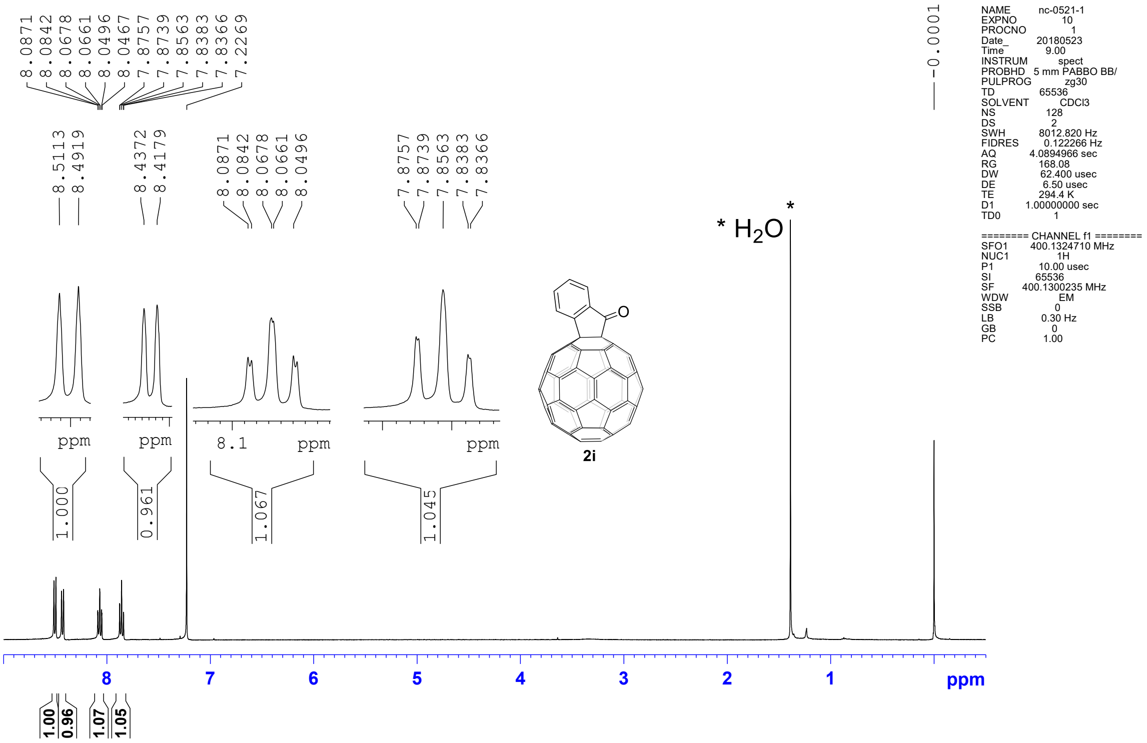

Figure S31. ^{1}H NMR (400 MHz, 1:1 $CS_2/CDCl_3$) of compound **2i**.

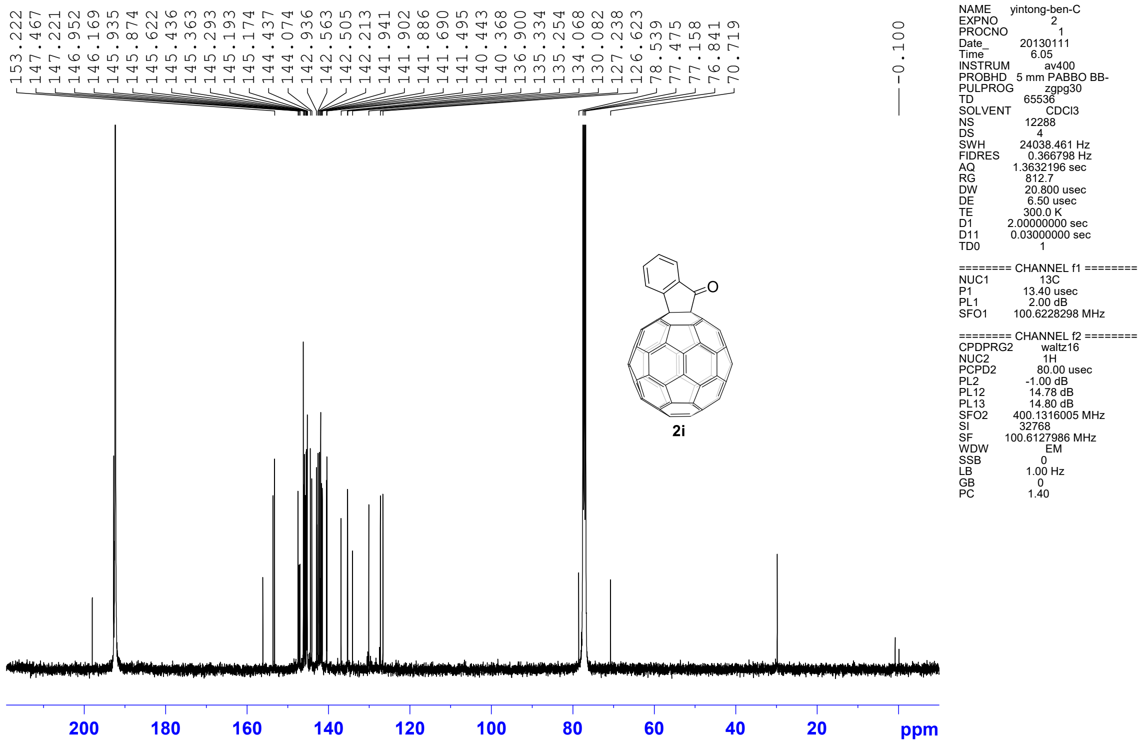

Figure S32. ^{13}C NMR (100 MHz, 1:1 $CS_2/CDCl_3$ with $Cr(acac)_3$ as a relaxation agent) of compound **2i**.

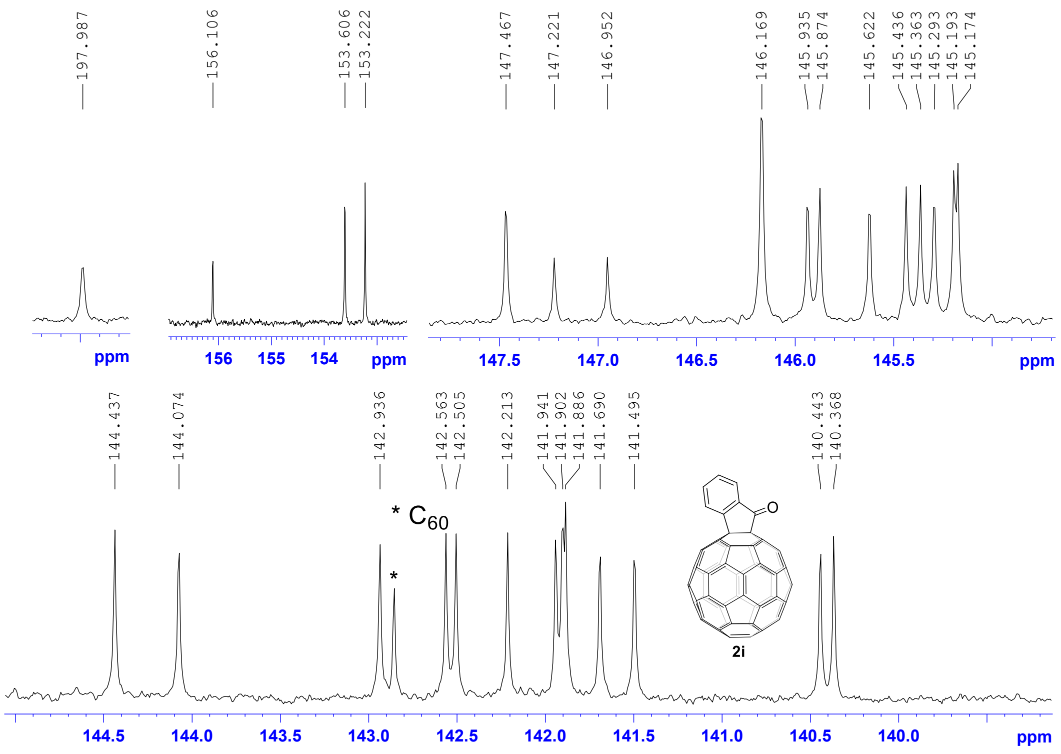

Figure S33. Expanded ^{13}C NMR (100 MHz, 1:1 $CS_2/CDCl_3$ with $Cr(acac)_3$ as a relaxation agent) of compound **2i**.

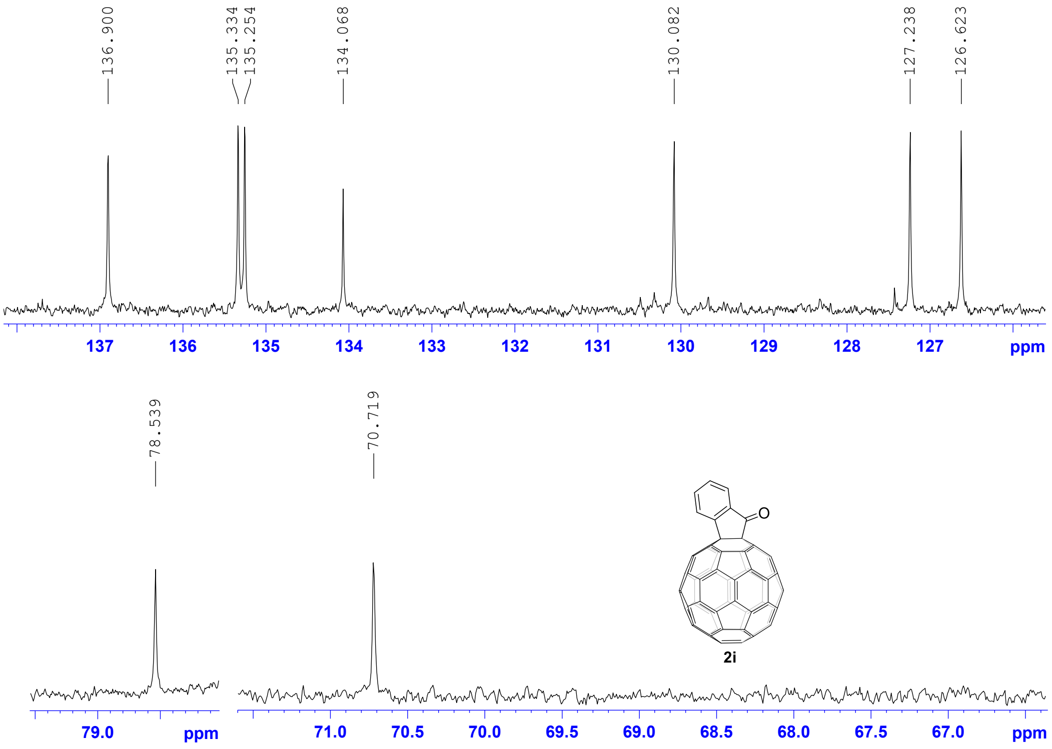

Figure S34. Expanded ^{13}C NMR (100 MHz, 1:1 CS_2/$CDCl_3$ with Cr(acac)$_3$ as a relaxation agent) of compound **2i**.

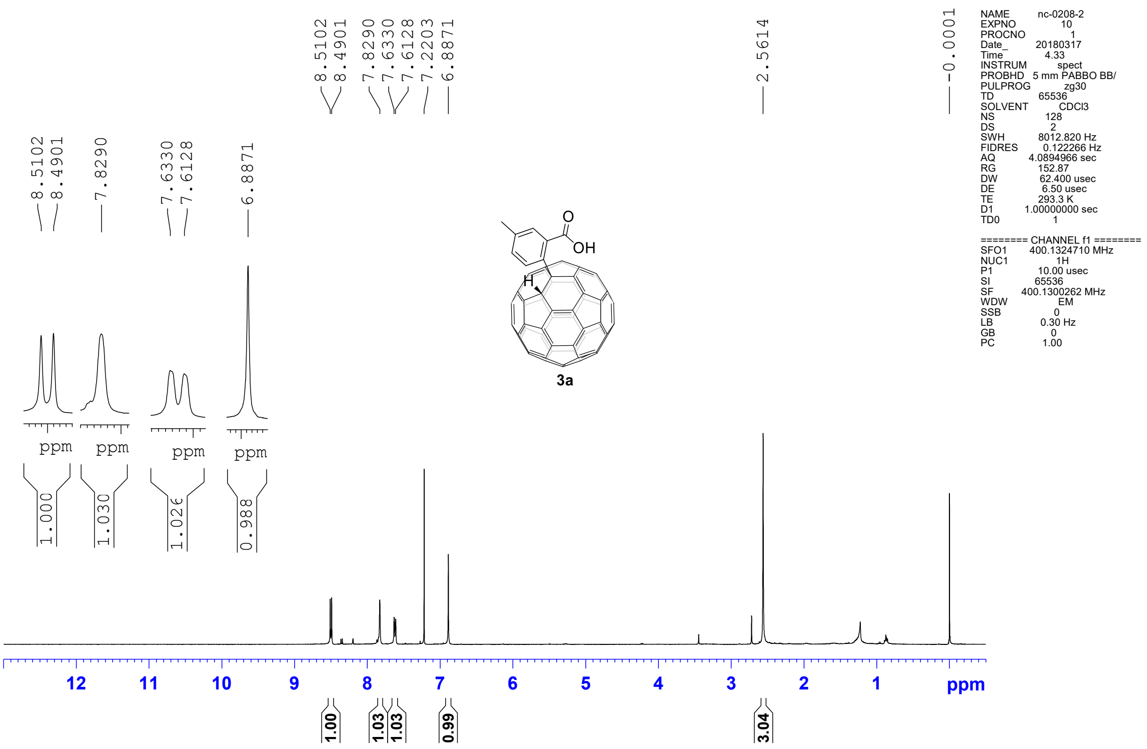

Figure S35. ^{1}H NMR (400 MHz, 1:1 CS_2/$CDCl_3$) of compound **3a**.

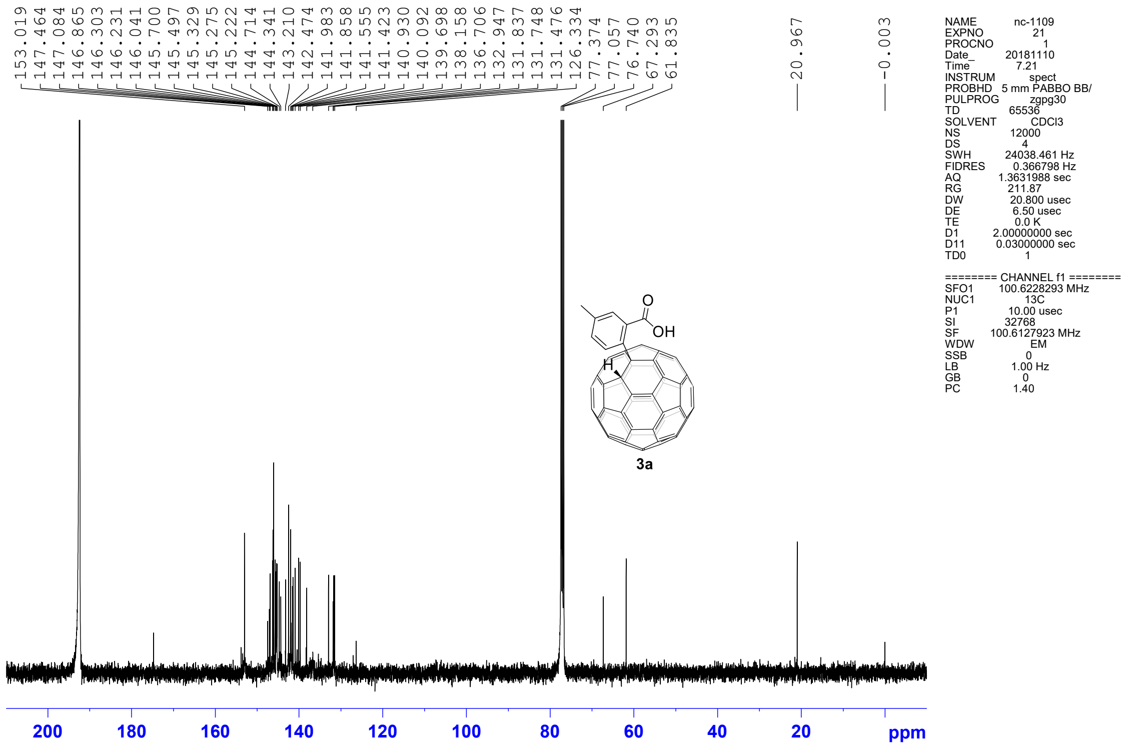

Figure S36. ^{13}C NMR (100 MHz, 1:1 $CS_2/CDCl_3$) of compound **3a**.

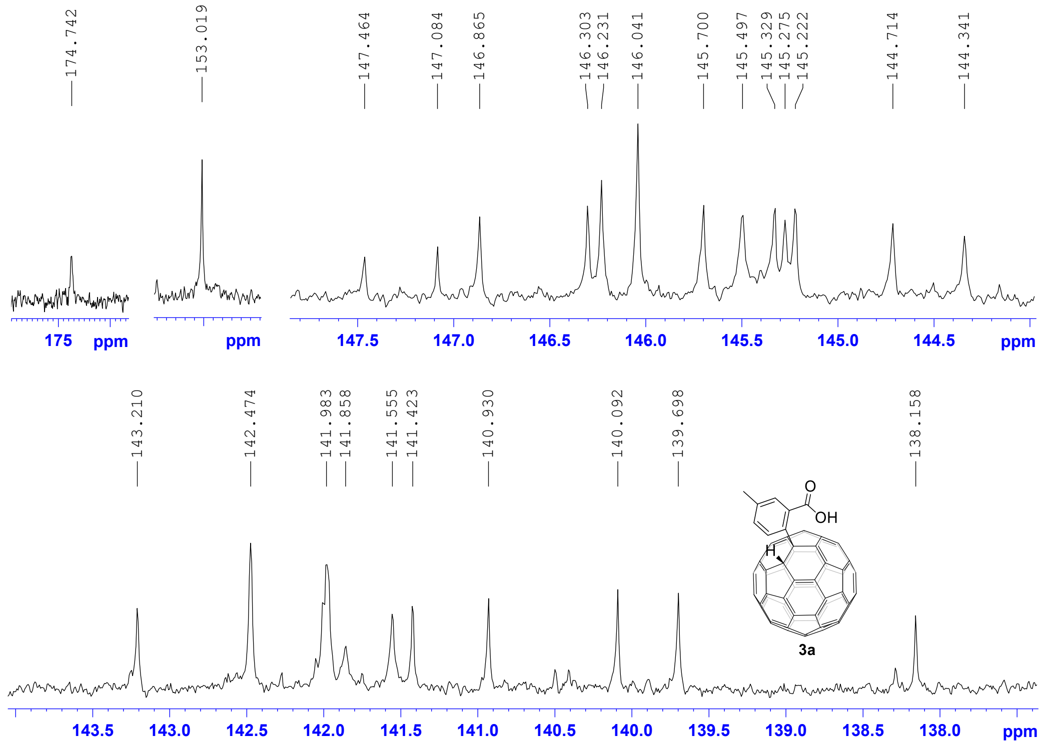

Figure S37. Expanded ^{13}C NMR (100 MHz, 1:1 $CS_2/CDCl_3$) of compound **3a**.

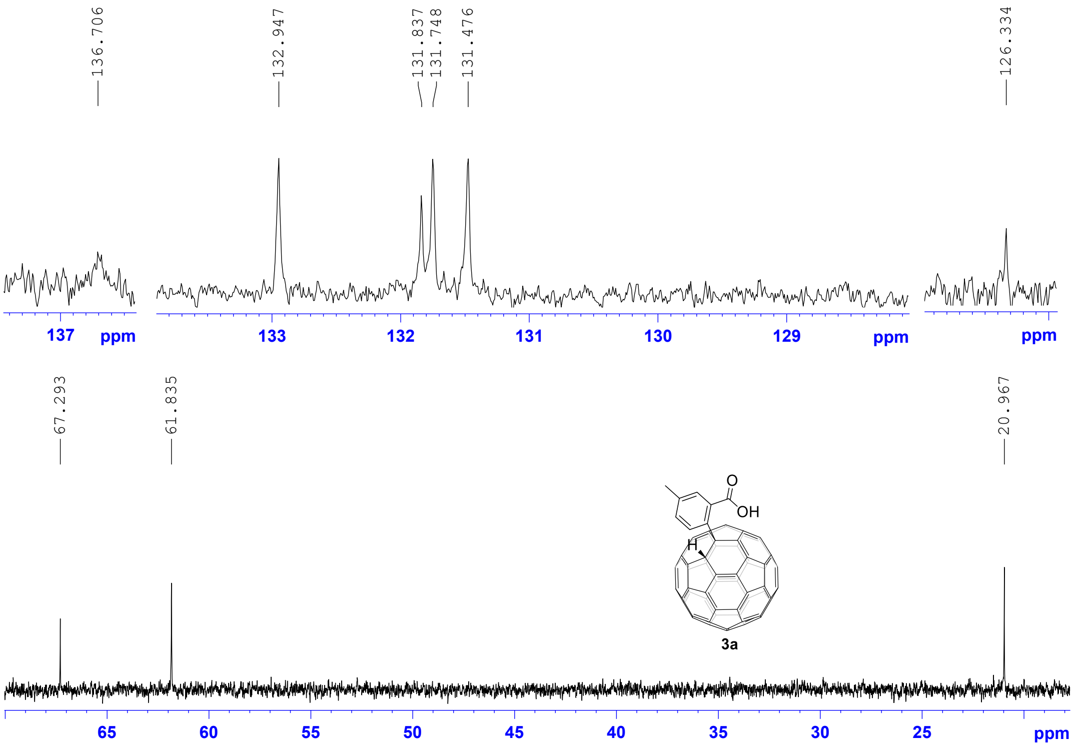

Figure S38. Expanded ^{13}C NMR (100 MHz, 1:1 $CS_2/CDCl_3$) of compound **3a**.

5. CVs of Compounds 1a–i, 2a-i, 3a along with C_{60}

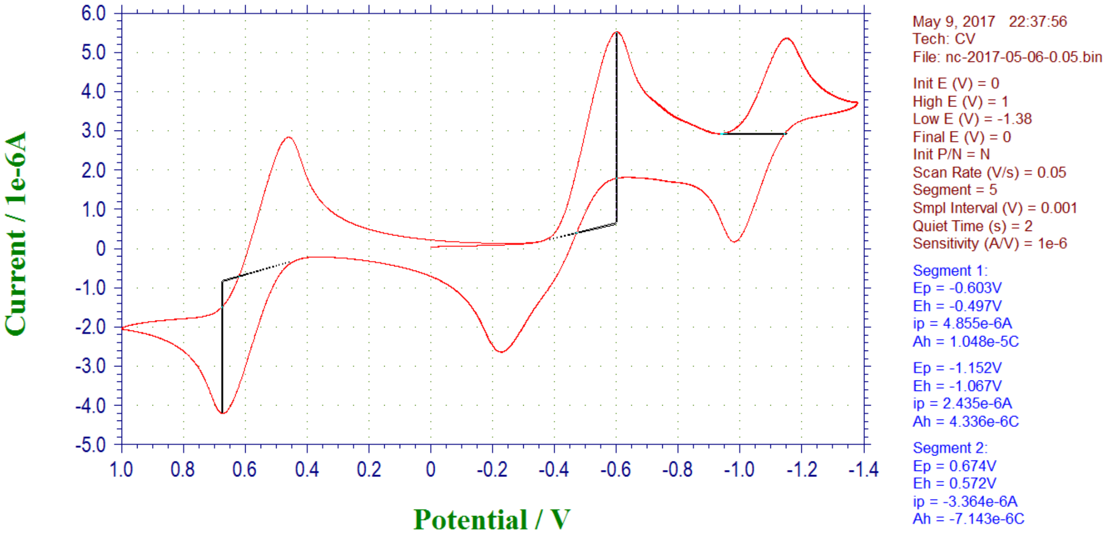

Figure S39. Cyclic voltammogram of compound **1a** (scanning rate: 50 mV s^{-1}).

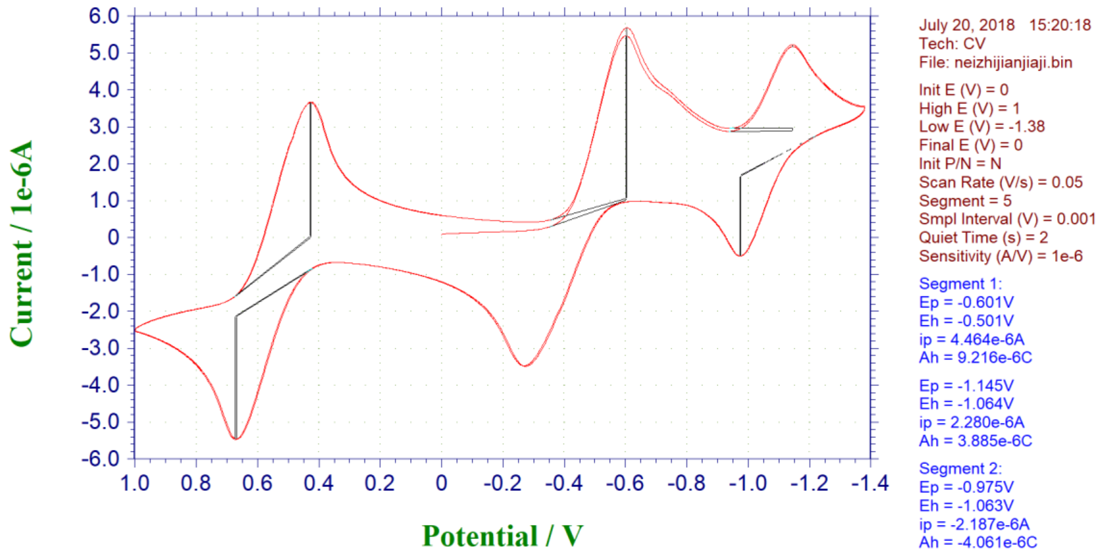

Figure S40. Cyclic voltammogram of compound **1b** (scanning rate: 50 mV s^{-1}).

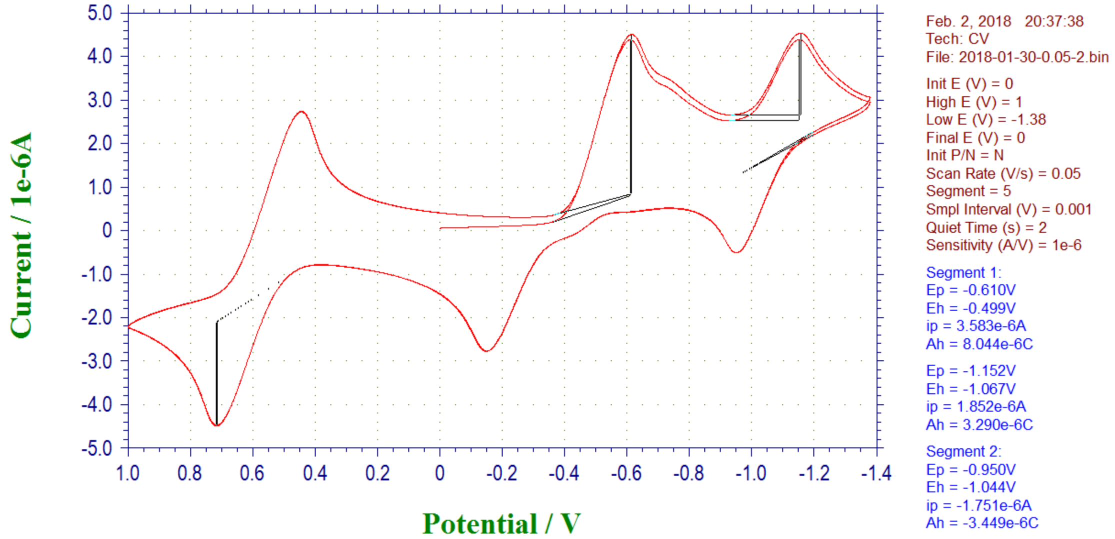

Figure S41. Cyclic voltammogram of compound **1c** (scanning rate: 50 mV s^{-1}).

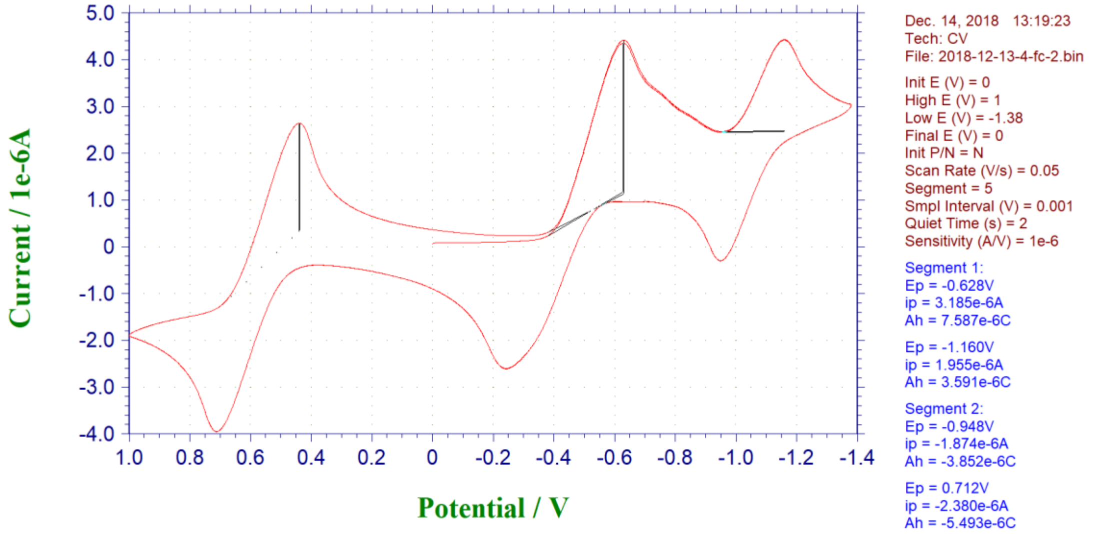

Figure S42. Cyclic voltammogram of compound **1d** (scanning rate: 50 mV s^{-1}).

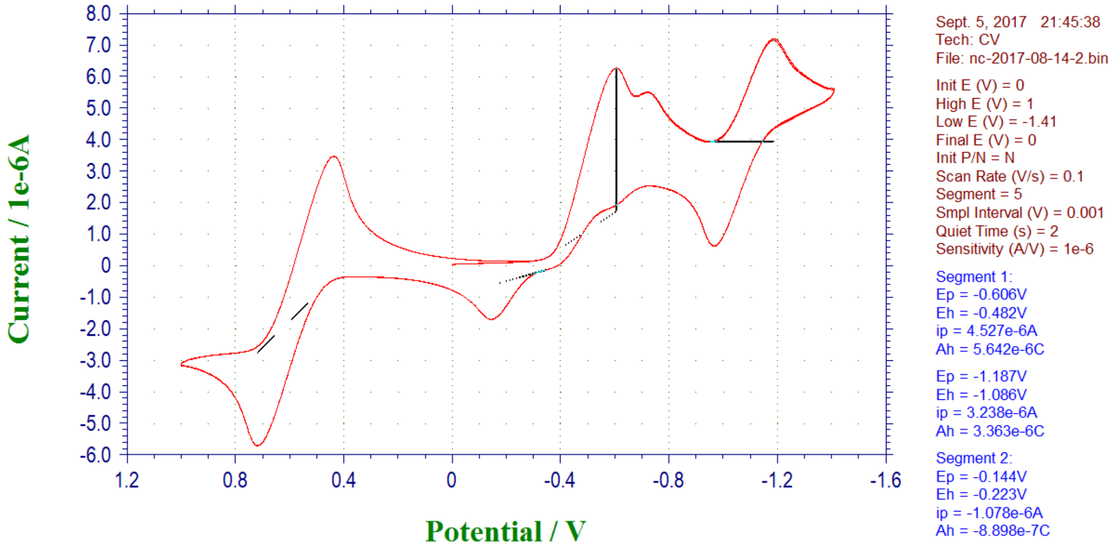

Figure S43. Cyclic voltammogram of compound **1e** (scanning rate: 50 mV s^{-1}).

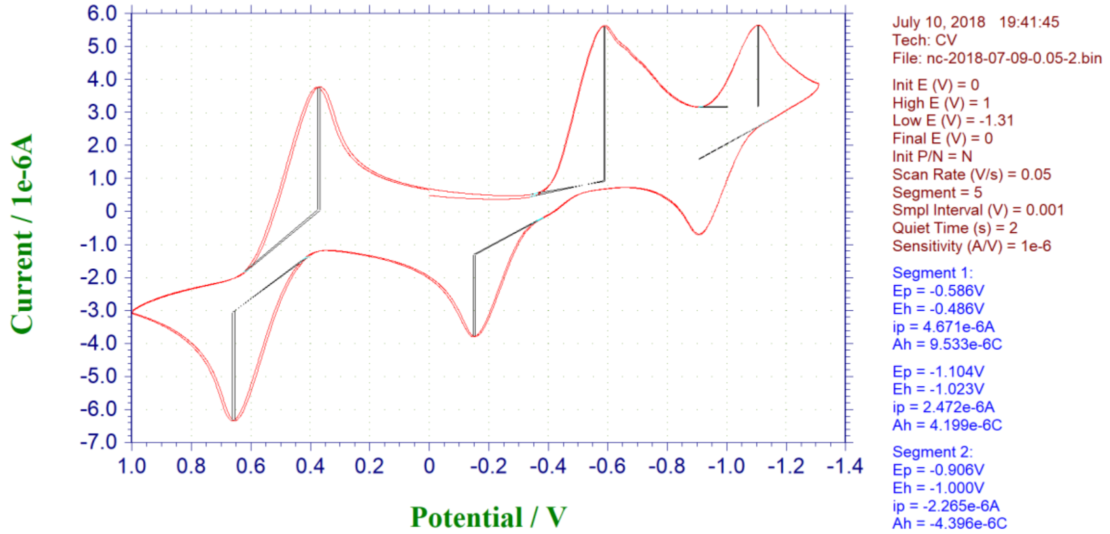

Figure S44. Cyclic voltammogram of compound **1f** (scanning rate: 50 mV s^{-1}).

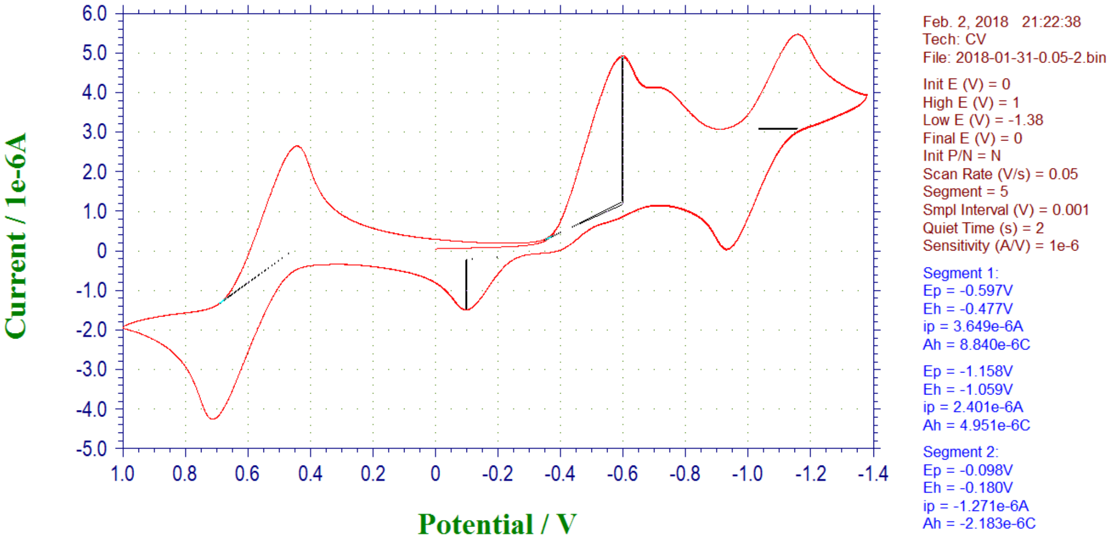

Figure S45. Cyclic voltammogram of compound **1g** (scanning rate: 50 mV s^{-1}).

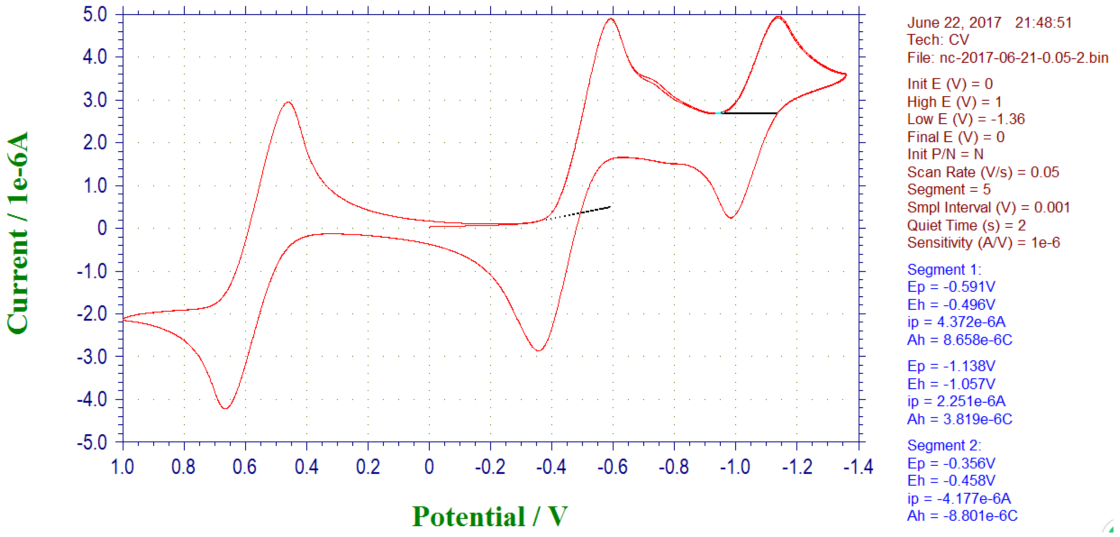

Figure S46. Cyclic voltammogram of compound **1h** (scanning rate: 50 mV s^{-1}).

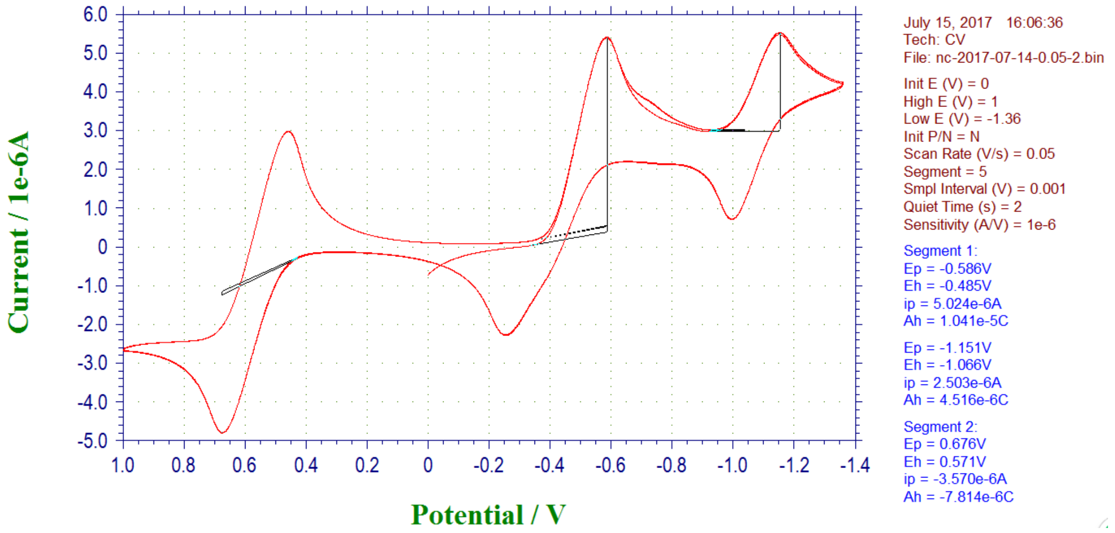

Figure S47. Cyclic voltammogram of compound **1i** (scanning rate: 50 mV s^{-1}).

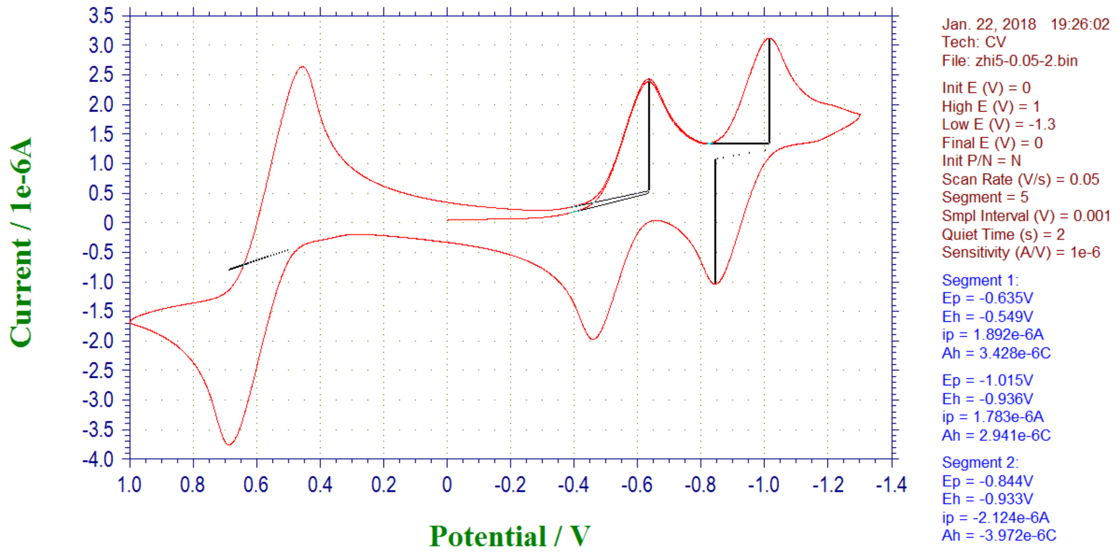

Figure S48. Cyclic voltammogram of compound **2a** (scanning rate: 50 mV s^{-1}).

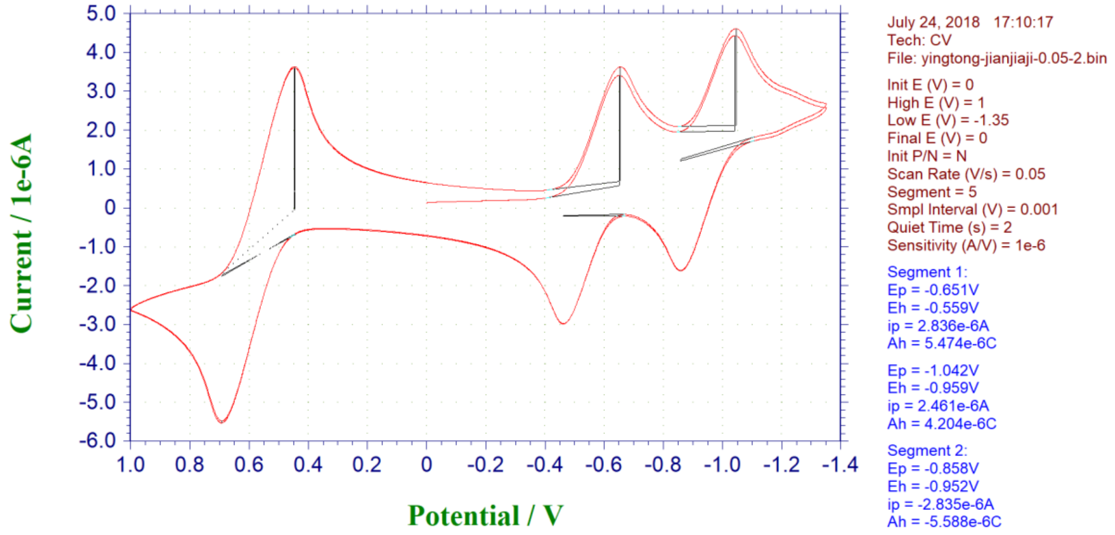

Figure S49. Cyclic voltammogram of compound **2b** (scanning rate: 50 mV s^{-1}).

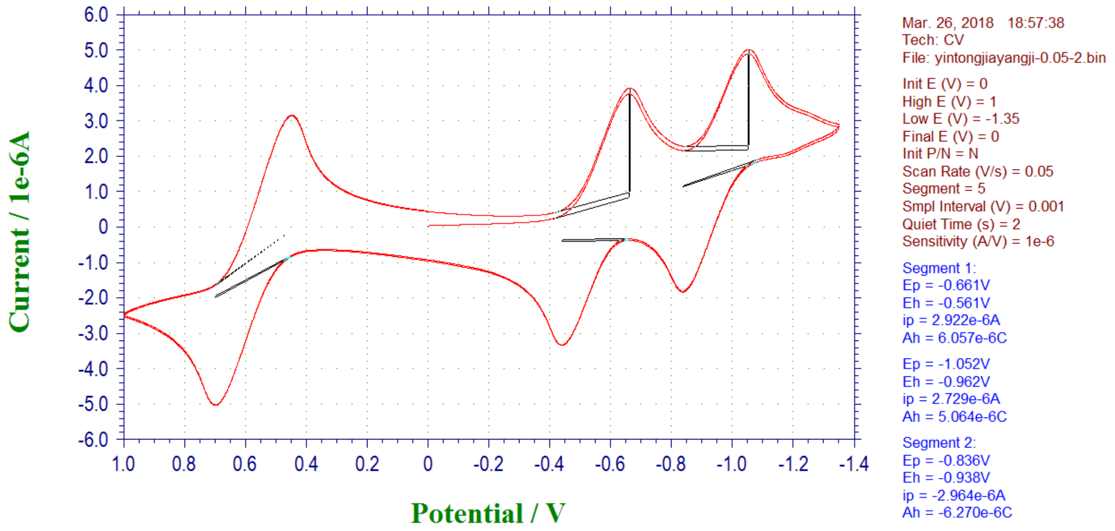

Figure S50. Cyclic voltammogram of compound **2c** (scanning rate: 50 mV s^{-1}).

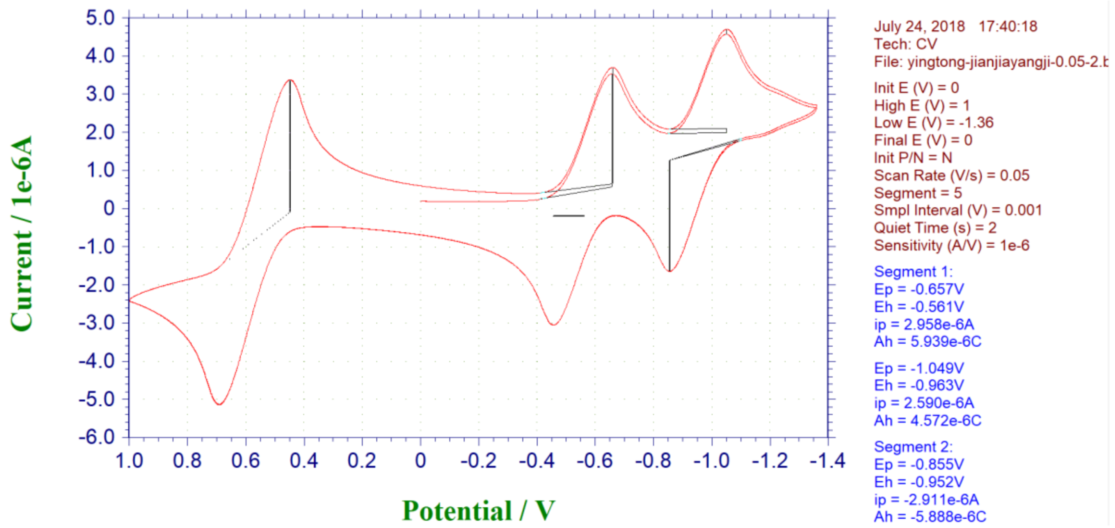

Figure S51. Cyclic voltammogram of compound **2d** (scanning rate: 50 mV s^{-1}).

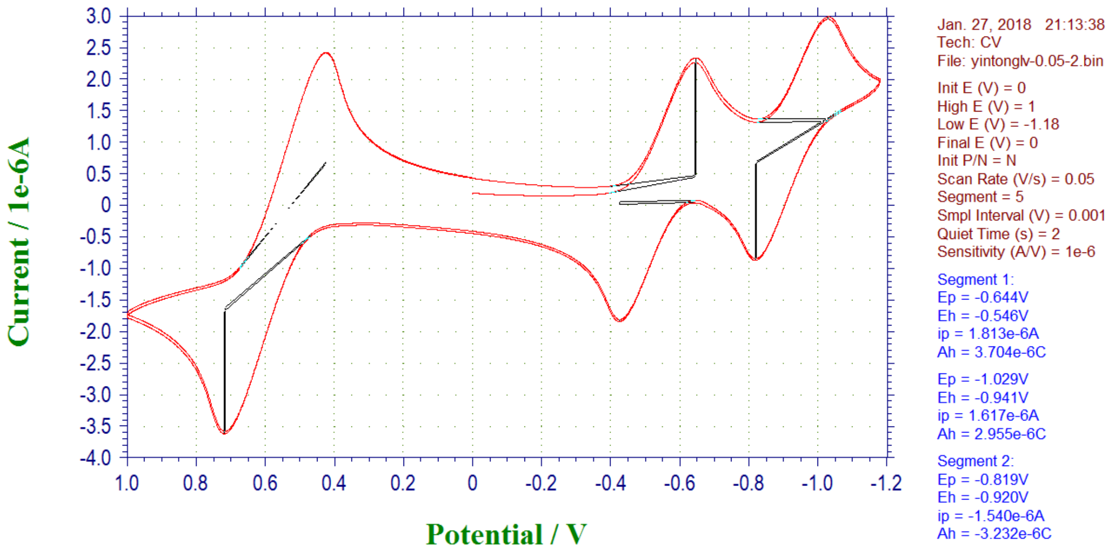

Figure S52. Cyclic voltammogram of compound **2e** (scanning rate: 50 mV s^{-1}).

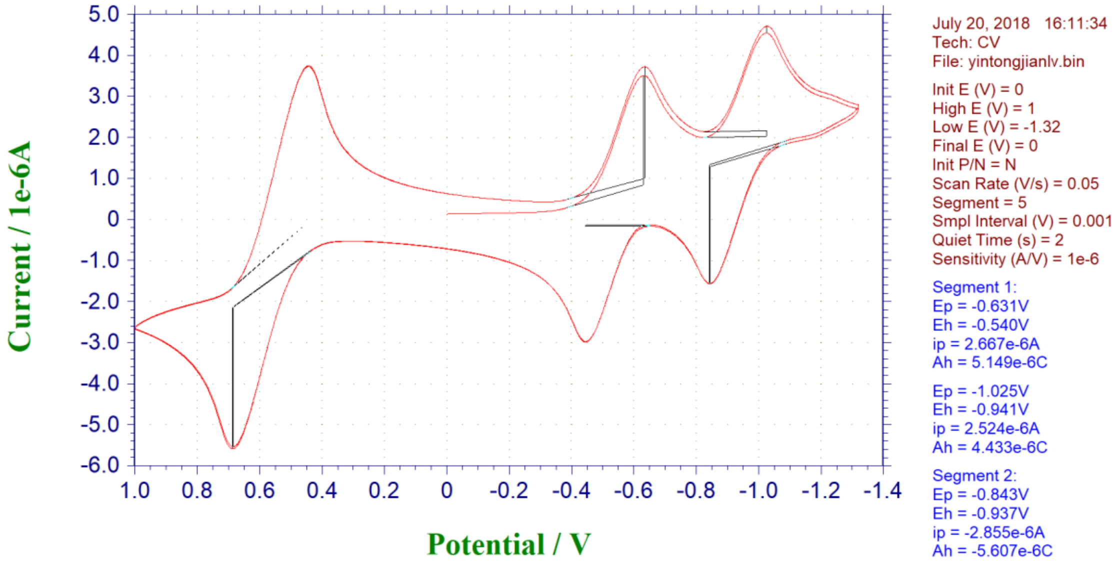

Figure S53. Cyclic voltammogram of compound **2f** (scanning rate: 50 mV s^{-1}).

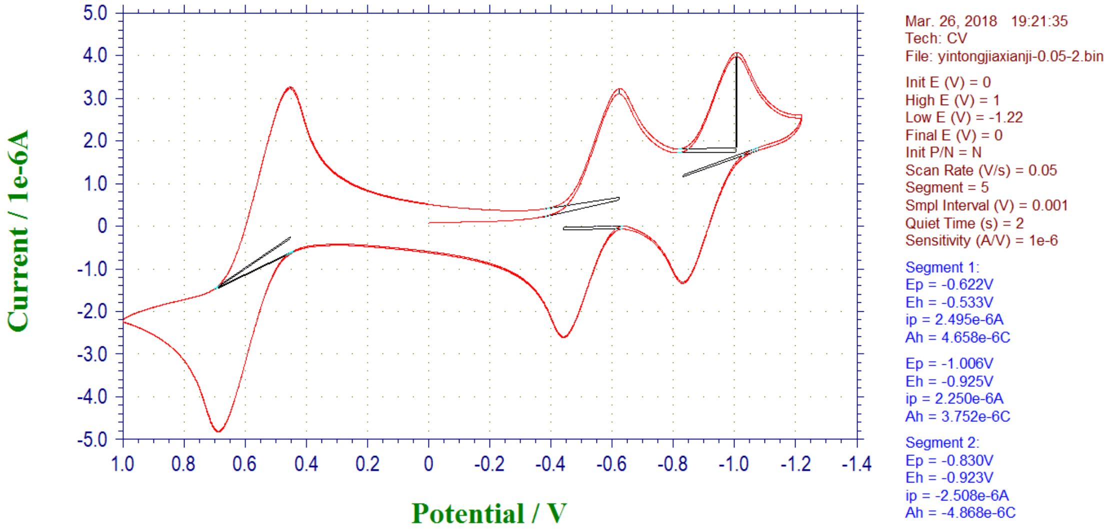

Figure S54. Cyclic voltammogram of compound **2g** (scanning rate: 50 mV s^{-1}).

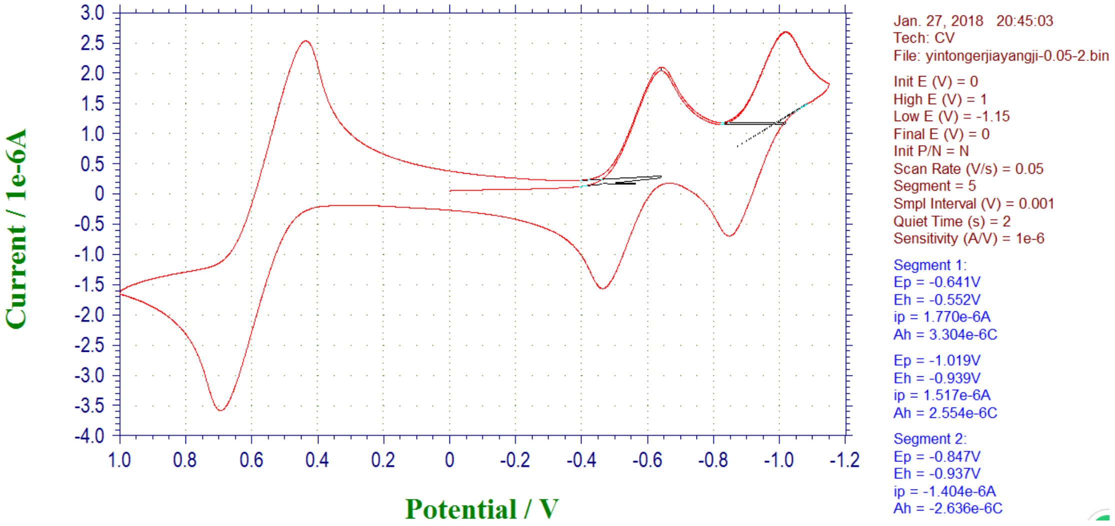

Figure S55. Cyclic voltammogram of compound **2h** (scanning rate: 50 mV s^{-1}).

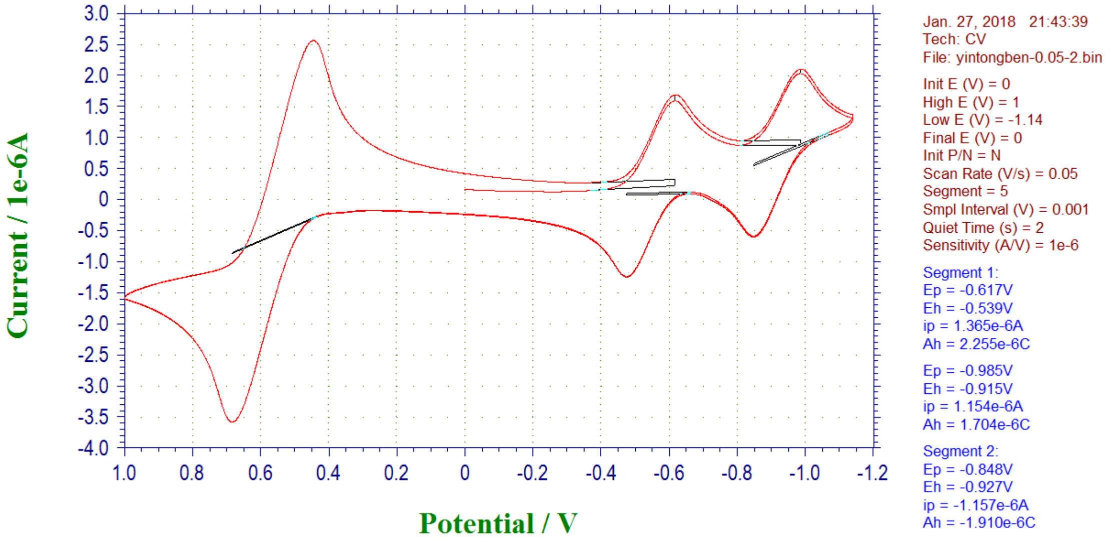

Figure S56. Cyclic voltammogram of compound **2i** (scanning rate: 50 mV s^{-1}).

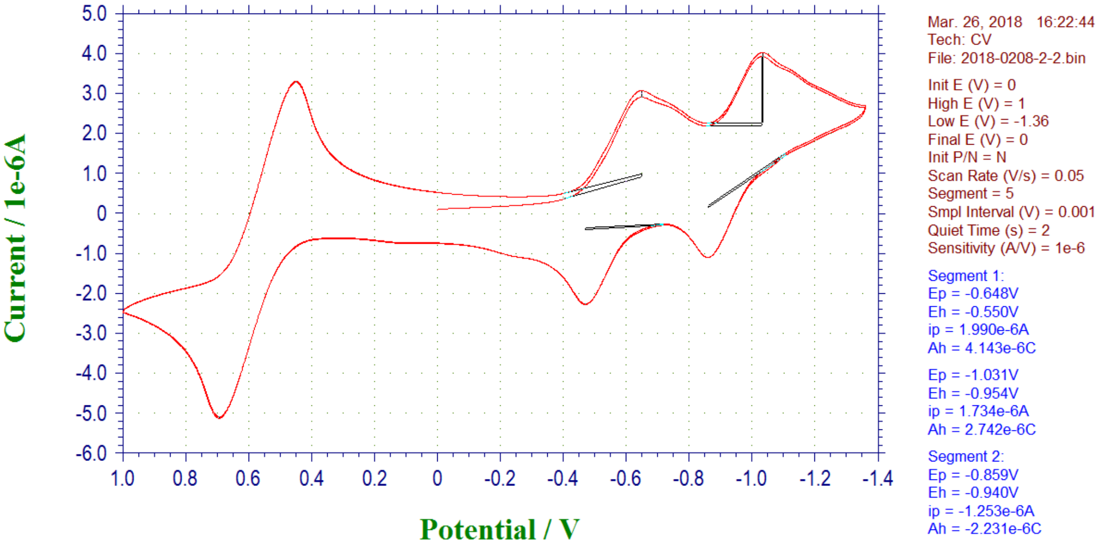

Figure S57. Cyclic voltammogram of compound **3a** (scanning rate: 50 mV s^{-1}).

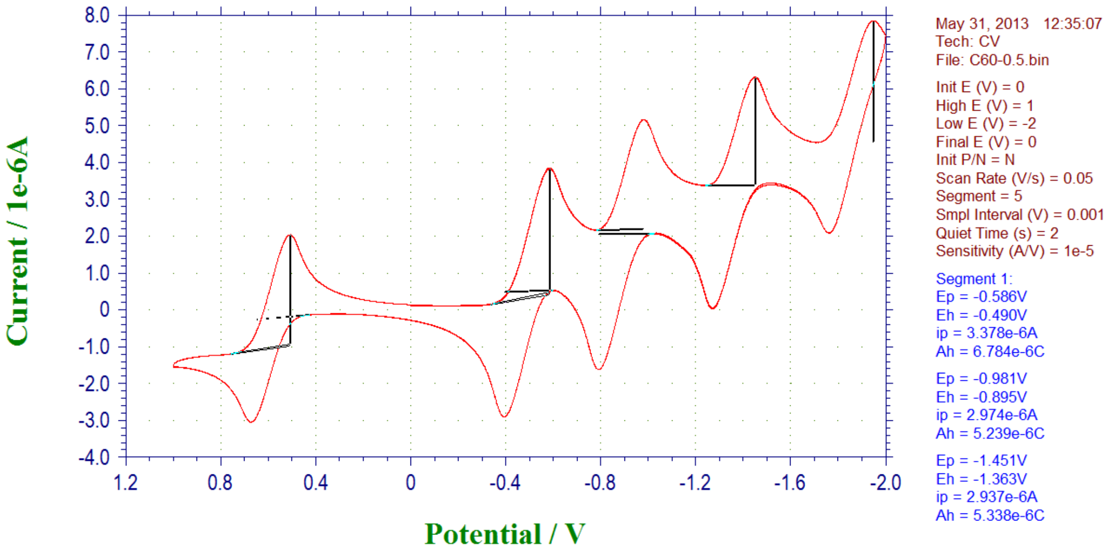

Figure S58. Cyclic voltammogram of compound C_{60} (scanning rate: 50 mV s^{-1}).

6. Reference

1 G.-W. Wang and B. Zhu, *Chem. Commun.*, 2009, 1769.
